# Discovery of new 6-ureido/amidocoumarins as highly potent and selective inhibitors for the tumour-relevant carbonic anhydrases IX and XII

**DOI:** 10.1080/14756366.2022.2154603

**Published:** 2023-02-02

**Authors:** Ashraf K. El-Damasy, Hyun Ji Kim, Alessio Nocentini, Seon Hee Seo, Wagdy M. Eldehna, Eun-Kyoung Bang, Claudiu T. Supuran, Gyochang Keum

**Affiliations:** a Center for Brain Technology, Brain Science Institute, Korea Institute of Science and Technology (KIST), Seoul, South Korea; b Department of Medicinal Chemistry, Faculty of Pharmacy, Mansoura University, Mansoura, Egypt; c Section of Pharmaceutical and Nutraceutical Sciences, Department of NEUROFARBA, University of Florence, Florence, Italy; d Laboratory of Molecular Modeling Cheminformatics & QSAR, Department of NEUROFARBA-Pharmaceutical and Nutraceutical Section, University of Firenze, Florence, Italy; e Department of Pharmaceutical Chemistry, Faculty of Pharmacy, Kafrelsheikh University, Kafr El Sheikh, Egypt; f Division of Bio-Medical Science & Technology, KIST School, Korea University of Science and Technology (UST), Seoul, South Korea

**Keywords:** Ureidocoumarins, carbonic anhydrase IX, carbonic anhydrase XII, SLC-0111, anticancer activity

## Abstract

A series of 6-ureido/amidocoumarins (**5a–p** and **7a–c**) has been designed and synthesised to develop potent and isoform- selective carbonic anhydrase *h*CA XI and XII inhibitors. All coumarin derivatives were investigated for their CA inhibitory effect against *h*CA I, II, IX, and XII. Interestingly, target coumarins potently inhibited both tumour-related isoforms *h*CA IX (*K*_I_s: 14.7–82.4 nM) and *h*CA XII (*K*_I_s: 5.9–95.1 nM), whereas the cytosolic off-target *h*CA I and II isoforms have not inhibited by all tested coumarins up to 100 μM. These findings granted the target coumarins an excellent selectivity profile towards both *h*CA IX and *h*CA XII isoforms, supporting their development as promising anticancer candidates. Moreover, all target molecules were evaluated for their anticancer activities against HCT-116 and MCF-7 cancer cells. The 3,5-bis-trifluoromethylphenyl ureidocoumarin **5i**, exerted the best anticancer activity. Overall, ureidocoumarins, particularly compound **5i,** could serve as a promising prototype for the development of potent anticancer CAIs.

## GRAPHICAL ABSTRACT

**Figure:**
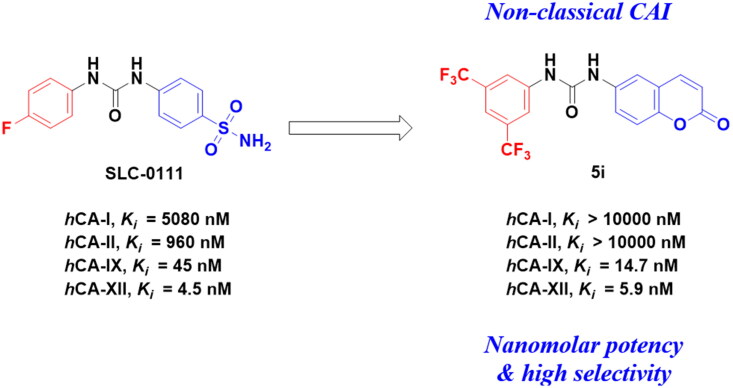

## Introduction

Carbonic anhydrases (CAs, EC 4.2.1.1) represent one of the most prevalent and well-explored metalloenzymes, usually containing zinc (II) ion at their active site^1^. CAs maintain pH homeostasis by catalysing the reversible hydration of carbon dioxide to bicarbonate and a proton^2^. Among the eight genetically distinct CA families, α-CAs are exclusively found in vertebrates^3^. With regard to human CAs (hCAs), 15 CA isozymes emerging from the α-family have been characterised. These isoforms differ in terms of their catalytic activity, protein structure, cellular localisation, and response to various types of modulators^4–6^.

hCA IX and XII are predominantly present in hypoxic cancers and contribute significantly to the metabolic and pH regulatory machine of tumour cells, supporting their proliferation^7–9^. CA IX is a transmembrane isoform, which is activated by the hypoxia-inducing factor-1α (HIF-1α) transcription factor under hypoxic circumstances. Compared to normal tissues, CA IX is highly overexpressed in various types of tumours, including breast and colorectal cancers^10^. CA XII, an extracellular facing membrane-bound CA, is upregulated in the hypoxic core of solid tumours as well as being colocated with P-glycoprotein (Pgp), the drug efflux protein, in a number of drug-resistant cancer cells^9^^,^^11^. Therefore, selective inhibition of hCA IX and/or XII over the ubiquitous cytosolic CA I and II isozymes has emerged as an effective approach for cancer therapy^12–15^.

Conventional CA inhibitors (CAIs) are mainly sulphonamide-based derivatives, where sulphonamide moiety serves as a zinc-binding group (ZBG). The major drawback observed for the majority of classical sulphonamide-based CAIs is the lack of selectivity among the various isoforms of CAs. However, adopting the so-called “tail approach”, where diverse chemical scaffolds were scouted as tail fragments attached to ZBG, proved its success in designing selective CA IX and XII inhibitors^16–19^. The most substantial example of the “tail” approach is the development of SLC-0111, a ureido-benzenesulfonamide selective CA IX inhibitor^20^^,^^21^, that is currently in Phase I/II clinical trials for the management of various metastatic hypoxic tumours^22^^,^^23^.

**Figure 1.**
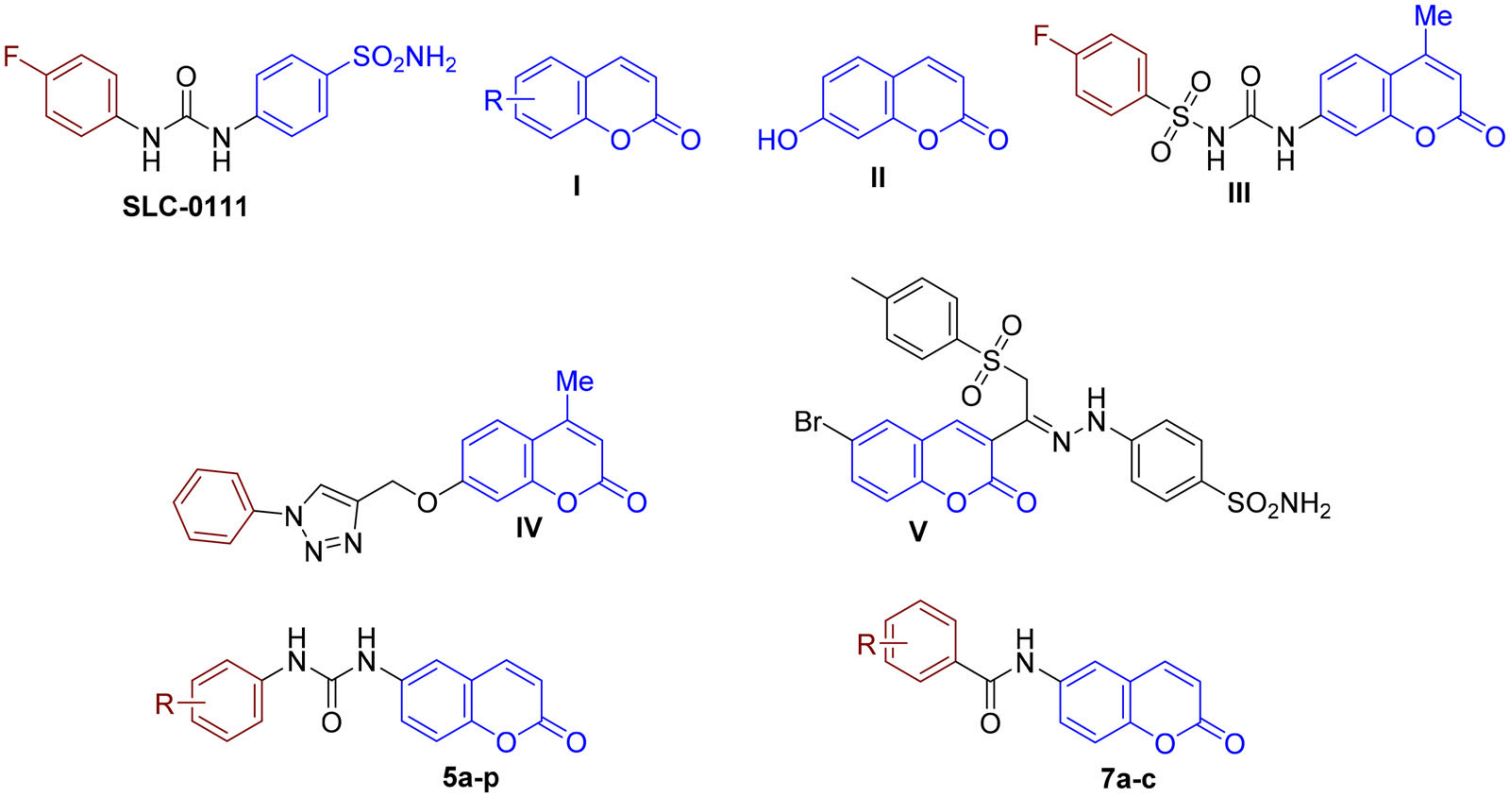
Chemical structure of SLC-0111, representative examples of reported coumarins as CAIs, and the designed compounds (**5a–p** and **7a–c**).

On the other hand, several coumarin derivatives, exemplified by compounds **I** and **II** (Figure 1), have been discovered as a privileged class of “non-classical CAIs”^24^^,^^25^. As demonstrated by Supuran’s group, *cis*-2-hydroxy-cinnamic acid, the product of coumarin hydrolysis, binds to the CA active site^24^. Interestingly, these identified coumarin-based CAIs displayed highly selective inhibitory effects towards the cancer-related isozymes (hCA IX and XII) rather than the ubiquitous CA I and II isozymes, which stem from the binding of the coumarin hydrolysis product at the entry gate for the active site cavity; the unique region which significantly varies amongst the different hCAs^9^^,^^26^. The emergence of coumarin as a promising CAI scaffold attracted the attention of medicinal chemists to develop a growing arsenal of structurally diverse coumarin-based CAIs with better selective inhibitory profiles against the tumour-relevant isozymes IX and XII, such as compounds **III–V** (Figure 1)^27–29^.

In the current study, we aimed at the development of potent and selective coumarin-based CAIs. Our design concept was relied mainly on replacing the typical sufonamide ZBG found in aromatic/heterocyclic/aliphatic/sugar sulphonamides with the privileged coumarin as a non-classical ZBG. In addition, various substitution patterns (*m*-/*p*-monosubstitution, 3,4/3,5/2,4/2,6-disubstitution, 2,4,6-trisubstitution) were installed on the aryl moiety tail to provide a lipophilic environment, which could be appropriate for the hydrophobic nature of the hCA IX active site, and to construct a reliable structure-activity relationships (SAR) (Figure 1). In view of the significance of the ureido linker for establishing crucial hydrogen bonds with certain backbone amino acids of CA IX, and hence favourable CA inhibition^30–35^, both coumarin and substituted aryl moiety were tethered through urea (**5a–p**). Moreover, the common urea spacer in coumarins derivatives (**5a–p**) was changed into amide (**7a–c)** to investigate the impact of such modification on CA inhibition. It is noteworthy mentioning that the designed molecules in this study intersect with those thiourediocoumarins recently reported with Thacker et al.^36^, which showed favourable CA IX and XIII inhibitory action. However, herein we focussed our efforts on introducing urea linker at C6 of coumarin instead of thiourea, believing that the bioisosteric replacement of thiourea with urea moiety might result in enhancement of ligand affinities for both hCA IX and XII as reported by Akgul et al.^37^. Moreover, we extensively explored a diverse set of substitution patterns on the aryl ring to identify the optimal hydrophobic appendage for achieving potent CA inhibitory activity.

## Results and discussion

### Chemistry

**Scheme 1.**
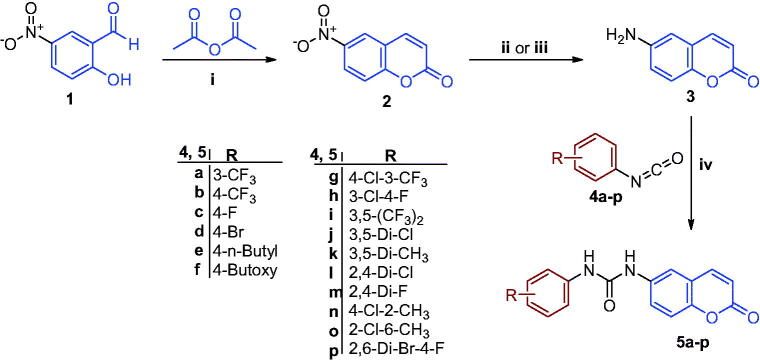
Reagents and reaction conditions: (i) Polyphosphoric acid, DMF, 145 °C, 6 h, 65%; (ii) SnCl_2_·2H_2_O, ethanol, reflux, 2 h, 71%; (iii) Fe powder, AcOH:EtOH:water (1:3:2 v/v), 40 °C, 1 h, 83%; (iv) Acetonitrile, rt, 2–18 h, DCM, rt, 18 h, 65–95%.

**Scheme 2.**
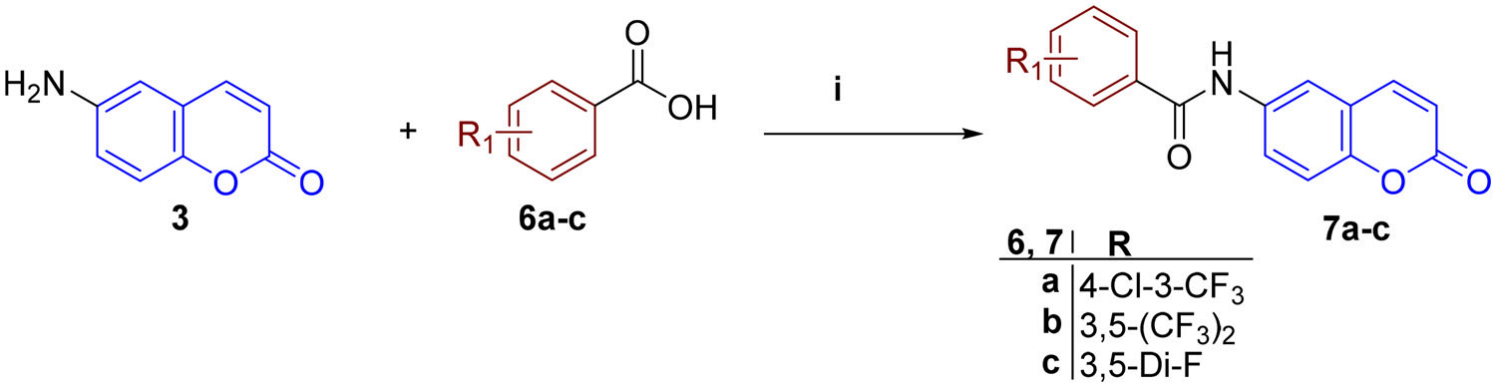
Reagents and reaction conditions: (i) Benzoic acid derivative, DIPEA, HATU, DMF, rt, 18 h, 25–80%.

As depicted in Scheme 1, the synthesis of the target ureidocoumarins **5a–p** was accomplished in a straightforward manner utilising 6-aminocoumarin **3** as the main building block. Treatment of 2-hydroxy-5-nitrobenzaldehyde **1** with acetic anhydride in a solution of polyphosphoric acid (PPA)/DMF at 145 °C yielded 6-nitrocoumarin **2**^38^. Reduction of **2** by either iron powder in AcOH:EtOH:water^36^, or SnCl_2_ dihydrate in ethanol^39^ afforded the corresponding amine **3** in good yield. Treatment of the amine with the appropriate phenyl isocyanate **4a–p** in acetonitrile under argon atmosphere gave the 6-ureidocoumarins **5a–p**. On the other hand, coupling of amine **3** with the pertinent benzoic acids **6a–c** was achieved using *O*-(7-azabenzotriazol-1-yl)-*N*,*N*,*N*′,*N*′-tetramethyluronium hexafluorophosphate (HATU) and diisopropylethylamine (DIPEA, Hünig’s base) in DMF under anhydrous conditions to afford the target 6-amidocoumarins **7a–c** (Scheme 2).

### Biological evaluation

#### Carbonic anhydrase inhibition

The target coumarins **5a–p** and **7a–c** were examined for their CA inhibitory actions towards the ubiquitous CA I and II (cytosolic) as well as the tumour-associated CA IX and XII isozymes by the use of a stopped-flow CO_2_ hydrase assay, using acetazolamide (AZA) as a reference compound. Close inspection of the inhibition data listed in Table 1 enabled the construction of reliable structure–activity relationships (SARs).

**Table 1.** CA inhibitory activity of compounds **5a–p**, **7a–c** against hCA isoforms I, II, IX, and XIII using AAZ as a standard CAI.

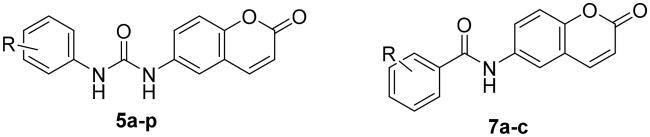					
Compound No.	R	Ki (nM)^a,b^			
		hCA I	hCA II	hCA IX	hCA XII
**5a**	3-CF_3_	>100 μM	>100 μM	45.9	26.2
**5b**	4-CF_3_	>100 μM	>100 μM	71.4	38.5
**5c**	4-F	>100 μM	>100 μM	41.5	40.2
**5d**	4-Br	>100 μM	>100 μM	70.6	51.1
**5e**	4-*n*-Butyl	>100 μM	>100 μM	54.7	95.1
**5f**	4-Butoxy	>100 μM	>100 μM	32.5	81.8
**5g**	4-Cl-3-CF_3_	>100 μM	>100 μM	66.4	29.4
**5h**	3-Cl-4-F	>100 μM	>100 μM	24.1	10.3
**5i**	3,5-(CF_3_)_2_	>100 μM	>100 μM	14.7	5.9
**5j**	3,5-Di-Cl	>100 μM	>100 μM	19.9	23.4
**5k**	3,5-Di-CH_3_	>100 μM	>100 μM	42.9	27.4
**5l**	2,4-Di-Cl	>100 μM	>100 μM	50.6	56.7
**5m**	2,4-Di-F	>100 μM	>100 μM	25.6	7.2
**5n**	4-Cl-2-CH_3_	>100 μM	>100 μM	28.4	43.1
**5o**	2-Cl-6-CH_3_	>100 μM	>100 μM	19.2	13.8
**5p**	2,6-Di-Br-4-F	>100 μM	>100 μM	72.8	50.4
**7a**	4-Cl-3-CF_3_	>100 μM	>100 μM	82.4	42.8
**7b**	3,5-(CF_3_)_2_	>100 μM	>100 μM	55.6	37.5
**7c**	3,5-Di-F	>100 μM	>100 μM	61.0	40.5
SLC-0111		5080	960.0	45.0	4.5
AAZ	–	250	12.5	25.0	5.7

^a^The presented values are the mean of three different experiments, and the errors were in the range of ±5–10% of the reported values.

^b^Incubation time of 6 h.

Results reported in Table 1 revealed that both the cytosolic off-target *h*CA I and II isoforms haven’t been inhibited by all herein reported coumarin-ureides **5a–p**, and coumarin-amides **7a–c** up to 100 μM concentration in the stopped-flow assay. Such diminished inhibitory activity against *h*CA I and *h*CA II granted the target coumarins excellent selectivity profile towards both tumour-related *h*CA IX and *h*CA XII isoforms that supports their development as promising anticancer candidates.

The main antitumor target *h*CA IX isoform was efficiently inhibited by all coumarin-ureides **5a–p** and coumarin-amides **7a–c** herein prepared. Noteworthy, the inhibition profile was found to be rather flat, since the measured *K*_I_s ranged from 14.7 to 66.4 nM, aside from coumarins **5b**, **5d**, **5p**, and **7a** whose efficacy raised at slightly higher concentrations (*K*_I_s = 71.4, 70.6, 72.8, and 82.4 nM, respectively). In particular, coumarin-ureides **5i**, **5j**, and **5o** stood out as the most effective *h*CA IX inhibitors in this study with *K*_I_s = 14.7, 19.9, and 19.2 nM, respectively (Table 1). In addition, coumarin-ureides **5a**, **5c**, **5f**, **5h**, **5k**, **5m**, and **5n** exerted excellent *h*CA IX inhibitory activity (*K*_I_s = 45.9, 41.5, 32.5, 24.1, 42.9, 25.6, and 28.4 nM, respectively) which is more or equipotent to that of SLC-0111 (*K*_I_ = 45 nM). It is noteworthy mentioning that all ureidocoumarins derivatives **5a–p** exerted superior *h*CA IX inhibitory potency (*K*_I_s = 14.7–72.8 nM) over the previously reported thioureidocoumarins (*K*_I_s = 78.5–741 nM)^36^, which reveal that ureido linker is favourable for achieving optimal inhibition of *h*CA IX than its corresponding thiourea counterpart.

It is interesting to note that the best *h*CA IX inhibitors reported in this study (**5h–j** and **5m–o**) possessed di-substituted phenyl group, which highlighted that di-substitution of the pendant phenyl moiety is advantageous for the *h*CA IX inhibitory activity. Di-substitution was best observed for 3,5-(CF_3_)_2_ (**5i**; *K*_I_ = 14.7 nM) > 2-Cl-6-CH_3_ (**5o**; *K*_I_ = 19.2 nM) > 3,5-(Cl)_2_ (**5j**; *K*_I_ = 19.9 nM) > 3-Cl-4-F (**5h**; *K*_I_ = 24.1 nM) > 2,4-(F)_2_ (**5m**; *K*_I_ = 25.6 nM) > 4-Cl-2-CH_3_ (**5n**; *K*_I_ = 28.4 nM). On the other hand, and regarding the mono-substituted counterparts (**5a-f**), it was found that grafting *para*-butoxy and *para*-fluoro substituents resulted in the most effective mono-substituted *h*CA IX inhibitors discovered here **5f** and **5c** with *K*_I_s = 32.5 and 41.5 nM, respectively (Table 1).

The data displayed in Table 1 ascribed to the all prepared coumarins **5a–p** and **7a–c** high efficacy in inhibiting the second examined tumour-related isoform *h*CA XII with *K*_I_s ranging in the nanomolar range between 5.9 and 95.1 nM. Two coumarin-ureide derivatives inhibited *h*CA XII in a single-digit nanomolar range, that are the di-substituted 3,5-(CF_3_)_2_ compound **5i** (*K*_I_ = 5.9 nM) and the di-substituted 2,4-(F)_2_ compound **5m** (*K*_I_ = 7.2 nM). Moreover, coumarins **5a**, **5g**, **5h**, **5j**, **5k**, and **5o** showed potent inhibitory action towards *h*CA XII with *K*_I_s spanned between 10.3 and 29.4 nM.

Further analysis of the obtained results pointed out that incorporation of the urea linker showed a generally improved inhibitory profile against *h*CA IX and *h*CA XII isoforms than utilisation of the amide one. Coumarin-ureides **5g** and **5i** displayed more enhanced inhibitory activities than their amide analogues **7a** and **7b** against *h*CA IX (*K*_I_s = 66.4 and 14.7 nM for **5g** and **5i**, and *K*_I_s = 82.4 and 55.6 nM for **7a** and **7b**, respectively) and *h*CA XII (*K*_I_s = 29.4 and 5.9 nM for **5g** and **5i**, and *K*_I_s = 42.8 and 37.5 nM for **7a** and **7b**, respectively) isoforms (Table 1).

#### Antiproliferative activity against HCT-116 and breast MCF-7 cell lines

Encouraged by the favourable selectivity profile of ureidocoumarins **5a–p** and amidocoumarins **7a–c** towards hCA IX and hCA XII, they were further examined for their prospective antiproliferative activity against human colorectal (HCT-116) and breast (MCF-7) cancer cell lines, adopting the MTT assay. The assay results have been expressed as percentage growth inhibition (%GI) at 100 and 10 µM and are listed in Table 2.

**Table 2.** *In vitro* antiproliferative activity of the target compounds against HCT116 and MCF7 human cancer cell lines and their CLogP values.

Compound No.	% Growth inhibition^a,b^				CLogP value^c^
	HCT116		MCF7		
	100 µM	10 µM	100 µM	10 µM	
**5a**	21.9 ± 4.6	8.0 ± 4.8	34.7 ± 4.4	3.7 ± 1.9	4.12
**5b**	**77.0 ± 4.0**	7.6 ± 2.8	**77.8 ± 5.1**	4.2 ± 1.4	4.12
**5c**	31.3 ± 4.6	10.9 ± 1.6	13.5 ± 4.3	9.5 ± 4.2	3.36
**5d**	31.7 ± 3.5	25.4 ± 2.7	18.5 ± 4.5	18.2 ± 3.2	4.08
**5e**	**65.0 ± 6.0**	20.1 ± 2.5	46.4 ± 4.9	38.2 ± 2.5	5.26
**5f**	**67.7 ± 0.6**	15.0 ± 5.2	46.9 ± 3.4	32.3 ± 2.6	4.70
**5g**	**90.5 ± 1.4**	15.2 ± 4.4	**92.3 ± 0.6**	10.0 ± 2.4	4.65
**5h**	**92.3 ± 1.2**	11.9 ± 1.5	**91.2 ± 1.0**	16.9 ± 5.3	4.08
**5i**	**94.2 ± 1.1**	**70.4 ± 2.6**	**93.9 ± 0.4**	51.5 ± 3.1	5.03
**5j**	**91.9 ± 1.1**	19.2 ± 3.8	**91.1 ± 0.6**	6.1 ± 2.0	4.65
**5k**	**87.4 ± 1.8**	14.5 ± 2.7	**66.7 ± 5.0**	33.2 ± 3.2	4.17
**5l**	6.7 ± 1.7	2.6 ± 1.8	6.6 ± 2.5	5.9 ± 2.7	4.09
**5m**	26.1 ± 4.0	25.7 ± 2.3	22.7 ± 3.3	15.2 ± 1.4	3.06
**5n**	20.5 ± 3.5	4.9 ± 2.5	19.9 ± 1.2	16.7 ± 4.9	3.86
**5o**	**78.6 ± 3.7**	9.7 ± 4.0	**72.8 ± 5.5**	30.3 ± 1.5	3.30
**5p**	**75.2 ± 5.8**	14.1 ± 3.0	**79.5 ± 2.4**	21.4 ± 3.0	3.98
**7a**	42.2 ± 2.5	14.0 ± 2.4	19.0 ± 2.1	9.9 ± 3.4	4.13
**7b**	**76.0 ± 2.5**	19.6 ± 2.4	62.9 ± 6.4	11.3 ± 4.0	4.51
**7c**	52.0 ± 5.1	21.8 ± 3.0	21.4 ± 4.7	21.1 ± 3.3	3.00

^a^The presented values are the mean of three different experiments with standard error of the mean (SEM).

^b^Bold figures refer to strong growth inhibition (GI > 65%).

^c^CLogP values were calculated by ChemDraw Professional 15.0 software.

Investigating the MTT assay results unveiled that the substitution pattern of the aryl moiety, along with the spacer tethering of both aryl and coumarin scaffold are the major contributing factors for controlling the anticancer activity of this new chemotypes of coumarins. The ureidocoumarins **5g** and **5i** showed superior antiproliferative activity to their corresponding amidocoumarins **7a** and **7b**. Such finding points out the significant nature of urea spacer for achieving favourable anticancer activity. Among urea-containing coumarins, the 3-trifluoromethyl-4-chlorophenyl ureidocoumarin **5g** and urea members **5h–k** with 3,5-disubstitutedphenyl moiety possess the best tumour growth inhibitory activity (HCT-116; %GI = 87.4–94.2, MCF-7; %GI = 66.7–93.9) at 100 µM. In particular, 3,5-bis (trifluoromethyl)phenyl ureidocoumarin **5i**, with the highest lipophilic character (CLogP), elicited remarkable antiproliferative activity at both 10 µM (%GI > 50) and 100 µM (%GI > 90) against HCT-116 and MCF-7 cell lines. In contrast, the 2,4-disubstituted coumarins **5l–n** exerted modest cytostatic activity (%GI < 30) over the tested cell lines even at 100 µM dose. Compound **5o**, the positional isomer of **5n**, elicited better tumour growth inhibitory activity at the tested doses. Referring to the monosubstituted ureidocoumarins **5a–f**, the best antiproliferative effect was noticed for the *p*-trifluoromethylphenyl member **5b** as well as the butyl substituted ureides **5e** and **5f**. Overall, compound **5i** stood out as the most potent anticancer derivative among the tested compounds.

## Conclusions

In this study, a new series of 6-ureido/amidocoumarins, featuring chemically variegate substituents on the aryl ring, has been designed and synthesised as potential selective inhibitors of the tumour-related CA IX and XII. Our SAR study underscored that all target coumarins possess high inhibitory potency and selectivity towards both tumour-relevant isoforms *h*CA IX and *h*CA XII with nanomolar *K*_I_s values. The urea linker as well as disubstituted phenyl group were found as prerequisite structural features for optimal *h*CA IX/XII inhibition. In addition, the target compounds were investigated for their anticancer activities against HCT-116 and MCF-7 cancer cell lines. The most lipophilic ureidocoumarin **5i** bearing 3,5-bis-trifluoromethylphenyl group elicited the best anticancer activity. In view of the obtained findings, ureidocoumarin derivatives, particularly compound **5i** might serve as promising CAIs, which could be further optimised to develop more potent anticancer candidates.

## Experimental

### General

All reactions and manipulations were conducted utilising standard Schlenk techniques. All solvents and reagents were obtained from commercial suppliers and were used without further purification. The reaction progress was monitored on TLC plate (Merck, silica gel 60F_254_). Flash column chromatography was carried out using silica gel (Merck, 230–400 mesh) and the mobile phase solvents are indicated as a mixed solvent with either given volume-to-volume ratios or as a percentage. Melting points were measured using OptiMelt MPA100 melting point apparatus and were uncorrected. ^1^H and ^13^C NMR spectra were recorded on a Bruker Avance 400 MHz spectrometer, using appropriate deuterated solvents, as noted. Chemical shifts (*δ*) are given in parts per million (ppm) upfield from tetramethylsilane (TMS) as internal standard, and s, d, t, and m are designated as singlet, doublet, triplet, and multiplet, respectively. Coupling constants (*J*) are reported in hertz (Hz). High resolution mass spectra (HRMS) were recorded on JMS 700 (Jeol, Japan) mass spectrometer, with magnetic sector-electric sector double focussing mass analyser, and FAB^+^ ion mode. The purity of all final compounds was >95%, as determined by NMR. Compounds **2**^38^ and **3**^36^^,^^39^ was prepared adopting the reported procedure.

### General procedure for synthesis of compounds 5a–p

A solution of the appropriate phenyl isocyanate (0.68 mmol) in anhydrous acetonitrile (2 ml) was added drop wise to a stirred solution of compound **3** (0.1 g, 0.62 mmol) in acetonitrile (2 ml). The reaction mixture was stirred at rt for 4–12 h under an argon atmosphere. The resulting solid was collected by filtration, washed with dichloromethane (DCM), and dried to afford the target compounds in pure form.

#### 1-(2-Oxo-2H-chromen-6-yl)-3-(3-(trifluoromethyl)phenyl)urea (5a)

White solid; yield 81.0%; m.p. 252–254 °C; ^1^H NMR (400 MHz, DMSO-*d_6_*) *δ* 9.11 (s, 1H), 9.01 (s, 1H), 8.10–8.07 (m, 2H), 7.95 (d, *J* = 2.4 Hz, 1H), 7.59–7.56 (m, 2H), 7.52 (t, *J* = 8.0 Hz, 1H), 7.36 (d, *J* = 9.2 Hz, 1H), 7.32 (d, *J* = 7.6 Hz, 1H), 6.49 (d, *J* = 9.6 Hz, 1H); ^13^C NMR (100 MHz, DMSO-*d_6_*) *δ* 160.55, 135.05, 149.29, 144.80, 140.93, 136.28, 130.36, 130.02 (d, *J* = 31 Hz), 126.03, 123.34, 122.37, 119.31, 118.63, 117.47, 117.10, 116.95, 114.70 (d, *J* = 4.1 Hz); HRMS (EI) *m/z* calcd. for C_17_H_11_F_3_N_2_O_3_ [M]^+^: 348.0722, found 348.0719.

#### 1-(2-Oxo-2H-chromen-6-yl)-4-(3-(trifluoromethyl)phenyl)urea (5b)

White solid; yield 77.0%; m.p. 276–278 °C; ^1^H NMR (400 MHz, DMSO-*d_6_*) *δ* 9.17 (s, 1H), 9.02 (s, 1H), 8.08 (d, *J* = 9.6 Hz, 1H), 7.92 (d, *J* = 2.4 Hz, 1H), 7.67 (q, *J* = 9.3 Hz, 4H), 7.60 (dd, *J* = 8.8, 2.4 Hz, 1H), 7.37 (d, *J* = 9.2 Hz, 1H), 6.49 (d, *J* = 9.2 Hz, 1H); ^13^C NMR (100 MHz, DMSO-*d_6_*) *δ* 160.54, 152.84, 149.33, 144.78, 143.81, 136.23, 126.54 (d, *J* = 4 Hz), 126.51 (d, *J* = 269 Hz), 123.34, 122.37 (q, *J* = 32 Hz), 119.31, 118.42, 117.45, 117.14, 116.98; HRMS (EI) *m/z* calcd. for C_17_H_11_F_3_N_2_O_3_ [M]^+^: 348.0722, found 348.0719.

#### 1-(4-Fluorophenyl)-3-(2-oxo-2H-chromen-6-yl)urea (5c)

Yellowish white solid; yield 92.5%; m.p. 249–250 °C; ^1^H NMR (400 MHz, DMSO-*d_6_*) *δ* 8.87 (s, 1H), 8.77 (s, 1H), 8.07 (d, *J* = 9.6 Hz, 1H), 7.89 (d, *J* = 2.4 Hz, 1H), 7.58 (dd, *J* = 8.8, 2.4 Hz, 1H), 7.49 (dd, *J* = 9.2, 4.8 Hz, 2H), 7.35 (d, *J* = 8.8 Hz, 1H), 7.13 (t, *J* = 9.0 Hz, 2H), 6.48 (d, *J* = 9.6 Hz, 1H); ^13^C NMR (100 MHz, DMSO-*d_6_*) *δ* 160.56, 157.90 (d, *J* = 237 Hz), 153.16, 149.11, 144.80, 136.64, 136.36 (d, *J* = 2.2 Hz), 123.14, 120.57 (d, *J* = 7.6 Hz), 119.28, 117.10 (d, *J* = 7.9 Hz), 116.90, 115.85, 115.63; HRMS (EI) *m/z* calcd. for C_16_H_11_FN_2_O_3_ [M]^+^: 298.0754, found 298.0753.

#### 1-(4-Bromophenyl)-3-(2-oxo-2H-chromen-6-yl)urea (5d)

White solid; yield 92.0%; m.p. 257–258 °C; ^1^H NMR (400 MHz, DMSO-*d_6_*) *δ* 8.93 (s, 1H), 8.89 (s, 1H), 8.08 (d, *J* = 9.6 Hz, 1H), 7.90 (d, *J* = 2.4 Hz, 1H), 7.58 (dd, *J* = 8.8, 2.4 Hz, 1H), 7.47 (s, 4H), 7.36 (d, *J* = 8.8 Hz, 1H), 6.49 (d, *J* = 9.6 Hz, 1H); ^13^C NMR (100 MHz, DMSO-*d_6_*) *δ* 160.55, 152.94, 149.20, 144.81, 139.49, 136.46, 131.99, 123.22, 120.71, 119.30, 117.27, 117.10, 116.95, 113.84; HRMS (EI) *m/z* calcd. for C_16_H_11_BrN_2_O_3_ [M]^+^: 357.9953, found 357.9955.

#### 1-(4-Butylphenyl)-3-(2-oxo-2H-chromen-6-yl)urea (5e)

Greyish white solid; yield 70.5%; m.p. 223–224 °C; ^1^H NMR (400 MHz, DMSO-*d_6_*) *δ* 8.84 (s, 1H), 8.63 (s, 1H), 8.07 (d, *J* = 9.6 Hz, 1H), 7.91 (d, *J* = 2.4 Hz, 1H), 7.58 (dd, *J* = 9.2, 2.4 Hz, 1H), 7.39–7.34 (m, 3H), 7.10 (d, *J* = 8.0 Hz, 2H), 6.48 (d, *J* = 9.2 Hz, 1H), 2.52 (t, *J* = 7.6 Hz, 2H), 1.53 (quint, *J* = 7.5 Hz, 2H), 1.30 (sext, *J* = 7.4 Hz, 2H), 0.90 (t, *J* = 7.2 Hz, 3H); ^13^C NMR (100 MHz, DMSO-*d_6_*) *δ* 160.57, 153.09, 149.03, 144.84, 137.63, 136.79, 136.36, 128.98, 123.01, 119.29, 118.90, 117.05, 116.95, 116.88, 34.63, 33.74, 22.17, 14.24; HRMS (EI) *m/z* calcd. for C_20_H_20_N_2_O_3_ [M]^+^: 336.1474, found 336.1472.

#### 1-(4-Butoxyphenyl)-3-(2-oxo-2H-chromen-6-yl)urea (5f)

Gray solid; yield 73.3%; m.p. 234–235 °C; ^1^H NMR (400 MHz, DMSO-*d_6_*) *δ* 8.80 (s, 1H), 8.53 (s, 1H), 8.07 (d, *J* = 9.6 Hz, 1H), 7.89 (d, *J* = 2.8 Hz, 1H), 7.58 (dd, *J* = 9.0, 2.6 Hz, 1H), 7.36 (t, *J* = 8.6 Hz, 3H), 6.87 (d, *J* = 8.8 Hz, 2H), 6.48 (d, *J* = 9.6 Hz, 1H), 3.92 (t, *J* = 6.4 Hz, 2H), 1.68 (quint, *J* = 7.0 Hz, 2H), 1.44 (sext, *J* = 7.4 Hz, 2H), 0.94 (t, *J* = 7.4 Hz, 3H); ^13^C NMR (100 MHz, DMSO-*d_6_*) *δ* 160.58, 154.49, 153.24, 148.97, 144.85, 136.90, 132.92, 122.99, 120.63, 119.28, 117.04, 116.91, 116.88, 115.08, 67.76, 31.32, 19.23, 14.18; HRMS (EI) *m/z* calcd. for C_20_H_20_N_2_O_4_ [M]^+^: 352.1423, found 352.1419.

#### 1-(4-Chloro-3-(trifluoromethyl)phenyl)-3-(2-oxo-2H-chromen-6-yl)urea (5g)

White solid; yield 80.0%; m.p. 253–255 °C; ^1^H NMR (400 MHz, DMSO-*d_6_*) *δ* 9.25 (s, 1H), 9.08 (s, 1H), 8.16 (s, 1H), 8.10 (d, *J* = 9.6 Hz, 1H), 7.94 (s, 1H), 7.64 (s, 2H), 7.58 (dd, *J* = 8.2, 1.2 Hz, 1H), 7.37 (d, *J* = 9.2 Hz, 1H), 6.50 (d, *J* = 9.6 Hz, 1H); ^13^C NMR (100 MHz, DMSO-*d_6_*) *δ* 160.51, 152.94, 149.38, 144.75, 139.71, 136.13, 132.42, 127.21 (d, *J* = 31 Hz), 124.64, 123.51(d, *J* = 12 Hz), 122.93, 121.93, 119.30, 117.61, 117.30 (d, *J* = 6 Hz), 117.09, 116.96; HRMS (EI) *m/z* calcd. for C_17_H_10_ClF_3_N_2_O_3_ [M]^+^: 382.0332, found 382.0329.

#### 1-(3-Chloro-4-fluorophenyl)-3-(2-oxo-2H-chromen-6-yl)urea (5h)

White solid; yield 85.4%; m.p. 232–233 °C; ^1^H NMR (400 MHz, DMSO-*d_6_*) *δ* 8.98 (s, 1H), 8.95 (s, 1H), 8.08 (d, *J* = 9.2 Hz, 1H), 7.91 (d, *J* = 2.8 Hz, 1H), 7.83 (dd, *J* = 6.6, 1.8 Hz, 1H), 7.58 (dd, *J* = 8.8, 2.8 Hz, 1H), 7.38–7.33 (m, 3H), 6.49 (d, *J* = 9.6 Hz, 1H); ^13^C NMR (100 MHz, DMSO-*d_6_*) *δ* 160.54, 152.90 (d, *J* = 240 Hz), 153.01, 149.24, 144.76, 137.33 (d, *J* = 2.8 Hz), 136.34, 123.29, 120.13, 119.63 (d, *J* = 18 Hz), 119.27, 119.08 (d, *J* = 6.7 Hz), 117.39, 117.19, 117.07, 116.93; HRMS (EI) *m/z* calcd. for C_16_H_10_ClFN_2_O_3_ [M]^+^: 332.0364, found 332.0361.

#### 1-(3,5-Bis(trifluoromethyl)phenyl)-3-(2-oxo-2H-chromen-6-yl)urea (5i)

White solid; yield 75.2%; m.p. decomposition at 289 °C; ^1^H NMR (400 MHz, DMSO-*d_6_*) *δ* 9.45 (s, 1H), 9.18 (s, 1H), 8.15 (s, 2H), 8.08 (d, *J* = 9.6 Hz, 1H), 7.96 (d, *J* = 2.4 Hz, 1H), 7.62 (s, 1H), 7.59 (dd, *J* = 8.8, 2.4 Hz, 1H), 7.35 (d, *J* = 8.8 Hz, 1H), 6.48 (d, *J* = 9.6 Hz, 1H); ^13^C NMR (100 MHz, DMSO-*d_6_*) *δ* 160.50, 152.95, 149.49, 144.73, 142.21, 135.93, 131.17 (q, *J* = 32 Hz), 123.77 (q, *J* = 271 Hz), 123.64, 119.28, 118.51, 117.89, 117.07, 116.97, 114.87; HRMS (EI) *m/z* calcd. for C_18_H_10_F_6_N_2_O_3_ [M]^+^: 416.0596, found 416.0595.

#### 1-(3,5-Dichlorophenyl)-3-(2-oxo-2H-chromen-6-yl)urea (5j)

Beige solid; yield 91.3%; m.p. 252–253 °C; ^1^H NMR (400 MHz, DMSO-*d_6_*) *δ* 9.08 (d, *J* = 11.2 Hz, 2H), 8.06 (d, *J* = 9.6 Hz, 1H), 7.90 (d, *J* = 2.4 Hz, 1H), 7.58–7.54 (m, 3H), 7.35 (d, *J* = 9.2 Hz, 1H), 7.14 (s, 1H), 6.48 (d, *J* = 9.2 Hz, 1H); ^13^C NMR (100 MHz, DMSO-*d_6_*) *δ* 160.51, 152.75, 149.38, 144.73, 142.61, 136.07, 134.54, 123.43, 121.44, 119.28, 117.61, 117.08, 116.97, 116.87; HRMS (EI) *m/z* calcd. for C_16_H_10_Cl_2_N_2_O_3_ [M]^+^: 348.0068, found 348.0064.

#### 1-(3,5-Dimethylphenyl)-3-(2-oxo-2H-chromen-6-yl)urea (5k)

White solid; yield 84.2%; m.p. 238–239 °C; ^1^H NMR (400 MHz, DMSO-*d_6_*) *δ* 8.84 (s, 1H), 8.57 (s, 1H), 8.07 (d, *J* = 9.6 Hz, 1H), 7.93 (d, *J* = 2.4 Hz, 1H), 7.56 (dd, *J* = 8.8, 2.4 Hz, 1H), 7.35 (d, *J* = 8.8 Hz, 1H), 7.10 (s, 2H), 6.63 (s, 1H), 6.49 (d, *J* = 9.6 Hz, 1H), 2.25 (s, 6H); ^13^C NMR (100 MHz, DMSO-*d_6_*) *δ* 160.57, 153.01, 149.04, 144.85, 139.85, 138.21, 136.73, 124.06, 123.00, 119.30, 117.06, 116.98, 116.91, 116.53, 21.59; HRMS (EI) *m/z* calcd. for C_18_H_16_N_2_O_3_ [M]^+^: 308.1161, found 308.1158.

#### 1-(2,4-Dichlorophenyl)-3-(2-oxo-2H-chromen-6-yl)urea (5l)

White solid; yield 67.0%; m.p. 291–292 °C; ^1^H NMR (400 MHz, DMSO-*d_6_*) *δ* 9.63 (s, 1H), 8.43 (s, 1H), 8.22 (d, *J* = 8.8 Hz, 1H), 8.08 (d, *J* = 9.6 Hz, 1H), 7.90 (d, *J* = 2.0 Hz, 1H), 7.61 (d, *J* = 2.4 Hz, 1H), 7.57 (dd, *J* = 9.0, 2.2 Hz, 1H), 6.49 (d, *J* = 9.2 Hz, 1H); ^13^C NMR (100 MHz, DMSO-*d_6_*) *δ* 160.51, 152.51, 149.32, 144.76, 136.17, 135.56, 129.02, 128.09, 126.71, 123.18, 123.00, 122.57, 119.36, 117.22, 117.12, 116.99; HRMS (EI) *m/z* calcd. for C_16_H_10_Cl_2_N_2_O_3_ [M]^+^: 348.0068, found 348.0065.

#### 1-(2,4-Difluorophenyl)-3-(2-oxo-2H-chromen-6-yl)urea (5m)

Yellow solid; yield 51.0%; m.p. decomposition at 302 °C; ^1^H NMR (400 MHz, DMSO-*d_6_*) *δ* 9.22 (s, 1H), 8.56 (s, 1H), 8.12–8.07 (m, 2H), 7.89 (d, *J* = 2.0 Hz, 1H), 7.57 (dd, *J* = 8.8, 2.4 Hz, 1H), 7.37–7.29 (m, 2H), 7.06 (t, *J* = 7.6 Hz, 1H), 6.49 (d, *J* = 9.6 Hz, 1H); ^13^C NMR (100 MHz, DMSO-*d_6_*) *δ* 160.53, 152.84, 149.22, 144.78, 136.35, 124.44, 122.96, 122.56 (d, *J* = 9.0 Hz), 119.34, 117.17, 116.98, 111.60, 111.38, 104.52, 104.28, 104.01; HRMS (EI) *m/z* calcd. for C_16_H_10_F_2_N_2_O_3_ [M]^+^: 316.0659, found 316.0660.

#### 1-(4-Chloro-2-methylphenyl)-3-(2-oxo-2H-chromen-6-yl)urea (5n)

Yellowish white solid; yield 81.0%; m.p. decomposition at 300 °C; ^1^H NMR (400 MHz, DMSO-*d_6_*) *δ* 9.26 (s, 1H), 8.10–8.06 (m, 2H), 7.91–7.89 (m, 2H), 7.58 (dd, *J* = 9.0, 2.6 Hz, 1H), 7.36 (d, *J* = 8.8 Hz, 1H), 7.29 (d, *J* = 2.4 Hz, 1H), 7.22 (dd, *J* = 8.8, 2.4 Hz, 1H), 6.49 (d, *J* = 9.6 Hz, 1H), 2.27 (s, 3H); ^13^C NMR (100 MHz, DMSO-*d_6_*) *δ* 160.55, 153.05, 149.11, 144.84, 136.82, 136.63, 130.46, 130.13, 126.79, 126.38, 122.93, 122.80, 119.34, 117.16, 116.94, 18.09; HRMS (EI) *m/z* calcd. for C_17_H_13_ClN_2_O_3_ [M]^+^: 328.0615, found 328.0613.

#### 1-(2-Chloro-6-methylphenyl)-3-(2-oxo-2H-chromen-6-yl)urea (5o)

White solid; yield 87.3%; m.p. decomposition at 305 °C; ^1^H NMR (400 MHz, DMSO-*d_6_*) *δ* 9.12 (s, 1H), 8.06–8.04 (m, 2H), 7.89 (d, *J* = 2.8 Hz, 1H), 7.61 (dd, *J* = 8.8, 2.4 Hz, 1H), 7.36 (t, *J* = 6.9 Hz, 2H), 7.26 (d, *J* = 6.4 Hz, 1H), 7.20 (t, *J* = 7.8 Hz, 1H), 6.48 (d, *J* = 9.6 Hz, 1H), 2.29 (s, 3H); ^13^C NMR (100 MHz, DMSO-*d_6_*) *δ* 160.59, 153.33, 149.00, 144.87, 139.10, 136.98, 134.34, 132.41, 129.54, 127.81, 127.31, 122.98, 119.24, 117.05, 116.91, 116.87, 19.04; HRMS (EI) *m/z* calcd. for C_17_H_13_ClN_2_O_3_ [M]^+^: 328.0615, found 328.0616.

#### 1-(2,6-Dibromo-4-fluorophenyl)-3-(2-oxo-2H-chromen-6-yl)urea (5p)

Beige solid; yield 85.0%; m.p. decomposition at 313 °C; ^1^H NMR (400 MHz, DMSO-*d_6_*) *δ* 9.15 (br s, 1H), 8.24 (s, 1H), 8.05 (d, *J* = 9.6 Hz, 1H), 7.89 (d, *J* = 2.4 Hz, 1H), 7.77 (d, *J* = 8.4 Hz, 2H), 7.61 (dd, *J* = 9.0, 2.6 Hz, 1H), 7.34 (d, *J* = 9.2 Hz, 1H), 6.47 (d, *J* = 9.6 Hz, 1H); ^13^C NMR (100 MHz, DMSO-*d_6_*) *δ* 160.57, 158.97, 153.03, 149.11, 144.86, 136.77, 133.30, 125.87 (d, *J* = 11 Hz), 123.12, 120.02, 119.77, 119.23, 117.05, 116.90; HRMS (EI) *m/z* calcd. for C_16_H_9_Br_2_FN_2_O_3_ [M]^+^: 453.8964, found 453.8963.

### General procedure for synthesis of compounds 7a–c

*N*,*N*-diisoprpoylethylamine (DIPEA) (0.331 ml, 1.86 mmol) and HATU (0.306 g, 0.81 mmol) were added to a mixture of compound **3** (0.1 g, 0.62 mmol) and the appropriate aryl carboxylic acid (0.81 mmol) in anhydrous DMF (2 ml). The reaction mixture was degassed under an argon atmosphere, stirred at rt for 18 h, and then quenched with water (30 ml). The aqueous layer was extracted with ethyl acetate (3 × 30 ml), and the combined organic layer was washed with brine, dried over anhydrous Na_2_SO_4_, and filtered. The solvent was distilled off under vacuum, and the obtained residue was purified by flash column chromatography, utilising the appropriate elution system to yield the titled compounds in pure form.

#### 4-Chloro-N-(2-oxo-2H-chromen-6-yl)-3-(trifluoromethyl)benzamide (7a)

The compound was purified by flash column chromatography using a mixture of hexane and ethyl acetate (3:1 v/v). White solid; yield 27%; m.p. 272–273 °C; ^1^H NMR (400 MHz, DMSO-*d_6_*) *δ* 10.71 (s, 1H), 8.40 (s, 1H), 8.27 (d, J = 8.0 Hz, 1H), 8.18 (s, 1H), 8.12 (d, J = 9.6 Hz, 1H), 7.94 (d, J = 8.4 Hz, 1H), 7.87 (d, J = 8.8 Hz, 1H), 7.44 (d, J = 9.2 Hz, 1H), 6.51 (d, J = 9.6 Hz, 1H); ^13^C NMR (100 MHz, DMSO-*d_6_*) *δ* 163.15, 159.90, 149.91, 144.25, 134.96, 133.76, 133.35, 131.98, 127.03, 124.64, 119.33, 118.64, 116.61; HRMS (EI) *m/z* calcd. for C_17_H_9_ClF_3_NO_3_ [M]^+^: 367.0223, found 367.0225.

#### N-(2-Oxo-2H-chromen-6-yl)-3,5-bis(trifluoromethyl)benzamide (7b)

The compound was purified by flash column chromatography using a mixture of hexane and ethyl acetate (1:1 v/v). Yellow solid; yield 60.0%; m.p. 240–241 °C; ^1^H NMR (400 MHz, DMSO-*d_6_*) *δ* 10.87 (s, 1H), 8.62 (s, 2H), 8.38 (s, 1H), 8.18 (d, J = 2.4 Hz, 1H), 8.13 (d, J = 9.6 Hz, 1H), 7.90 (dd, J = 8.9, 2.4 Hz, 1H), 7.45 (d, J = 9.2 Hz, 1H), 6.52 (d, J = 9.6 Hz, 1H); ^13^C NMR (100 MHz, DMSO-*d_6_*) *δ* 162.56, 159.89, 150.03, 144.22, 136.75, 134.79, 130.65, 130.32, 128.54, 124.74, 124.43, 121.72, 119.49, 118.67, 116.66; HRMS (EI) *m/z* calcd. for C_18_H_9_F_6_NO_3_ [M]^+^: 401.0487, found 401.0490.

#### 3,5-Difluoro-N-(2-oxo-2H-chromen-6-yl)benzamide (7c)

The compound was purified by flash column chromatography using a mixture of hexane and ethyl acetate (2:1 v/v). White solid; yield 68.2%; m.p. 301–303 °C; ^1^H NMR (400 MHz, DMSO-*d_6_*) *δ* 10.56 (s, 1H), 8.20 (d, *J* = 2.4 Hz, 1H), 8.12 (d, *J* = 9.6 Hz, 1H), 7.87 (dd, *J* = 9.0, 2.2 Hz, 1H), 7.70 (d, *J* = 6.4 Hz, 2H), 7.55 (t, *J* = 9.0 Hz, 1H), 7.44 (d, *J* = 9.2 Hz, 1H), 6.52 (d, *J* = 9.6 Hz, 1H); ^13^C NMR (100 MHz, DMSO-*d_6_*) *δ* 164.00 (d, *J* = 17 Hz), 163.40, 161.26 (d, *J* = 25 Hz), 160.43, 150.42, 144.79, 138.45, 135.44, 125.09, 119.74, 119.14, 117.12 (d, *J* = 4.0 Hz), 111.63 (d, *J* = 27 Hz), 107.70; HRMS (EI) *m/z* calcd. for C_16_H_9_F_2_NO_3_ [M]^+^: 301.0550, found 301.0552.

### In vitro *evaluation of CA inhibitory activity*

The experimental procedures utilised for CA inhibitory assay of the target compounds were carried out as described earlier^40^, and presented in the Supplementary Materials.

### Cell based investigation of anticancer activity

The evaluation of anticancer activity of the target compounds was conducted by MTT assay adopting the literature procedure^41^.

## Supplementary Material

Supplemental Material
