# Supplementary material for: Discovery of new 6-ureido/amidocoumarins as highly potent and selective inhibitors for the tumour-relevant carbonic anhydrases IX and XII
Source: J Enzyme Inhib Med Chem. 2023 Feb 2;38(1):2154603. doi: 10.1080/14756366.2022.2154603 (PMC9897768; doi:10.1080/14756366.2022.2154603)

Supporting information

Discovery of new 6-ureido/amidocoumarins as highly potent and selective inhibitors for the tumor-relevant carbonic anhydrases IX and XII

Ashraf K. El-Damasy[a,b,*], Hyun Ji Kim[a], Alessio Nocentini[c,d], Seon Hee Seo[a], Wagdy M. Eldehna[e], Eun-Kyoung Bang[a], Claudiu T. Supuran[c,*], Gyochang Keum[a,f*]

[a] *Center for Brain Technology, Brain Science Institute, Korea Institute of Science and Technology (KIST), Seoul 02792, Republic of Korea*
[b]*Department of Medicinal Chemistry, Faculty of Pharmacy, Mansoura University, Mansoura 35516, Egypt*
[c]*Department of NEUROFARBA, Section of Pharmaceutical and Nutraceutical Sciences, University of Florence, Polo Scientifico, Via U. Schiff 6, 50019, Sesto Fiorentino, Firenze, Italy*
[d]*Department of NEUROFARBA-Pharmaceutical and Nutraceutical Section, Laboratory of Molecular Modeling Cheminformatics & QSAR, University of Firenze, Via U. Schiff 6, 50019, Sesto Fiorentino, Firenze, Italy*
[e] *Department of Pharmaceutical Chemistry, Faculty of Pharmacy, Kafrelsheikh University, Kafrelsheikh 33516, Egypt*
[f] *Division of Bio-Medical Science & Technology, KIST School, Korea University of Science and Technology (UST), Seoul, 02792, Republic of Korea*

Corresponding author:
Ashraf K. El-Damasy, E-mail: ashraf.el-damasy@kist.re.kr
Gyochang Keum, E-mail: gkeum@kist.re.kr
Claudiu T. Supuran, E-mail: claudiu.supuran@unifi.it

**Contents**	**Pages**
1) ^{1}H NMR and ^{13}C NMR spectra	2–20
2) HRMS charts	21–39

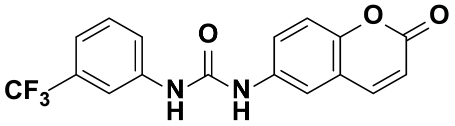

5a

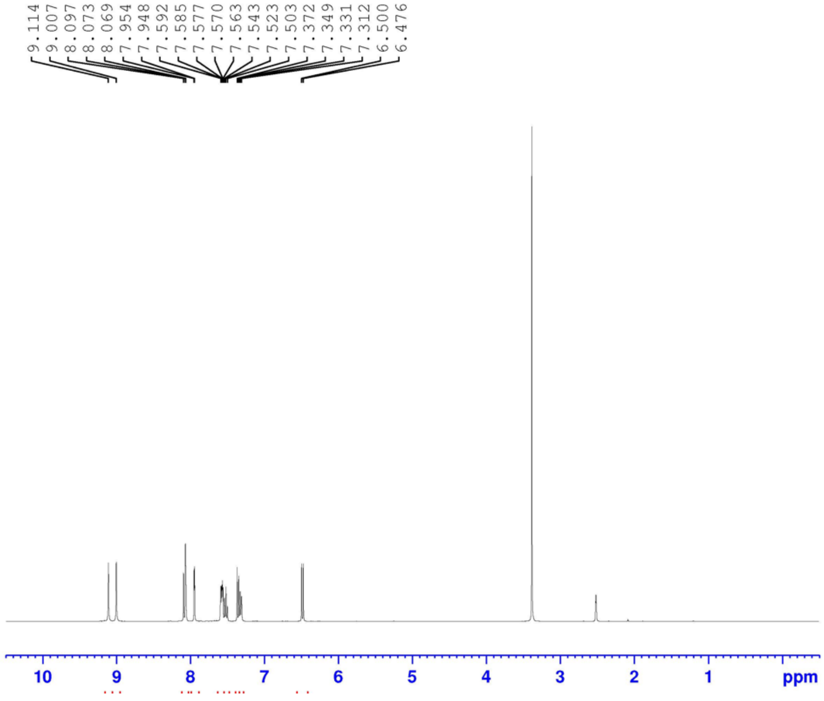

Current Data Parameters
NAME AKA-VII-81
EXPNO 1
PROCNO 1

F2 - Acquisition Parameters
Date_ 20201008
Time 18.04
INSTRUM spect
PROBHD 5 mm PABBO BB/
PULPROG zg30
TD 65536
SOLVENT DMSO
NS 16
DS 2
SWH 8012.820 Hz
FIDRES 0.122266 Hz
AQ 4.0894465 sec
RG 103.95
DW 62.400 usec
DE 6.50 usec
TE 297.9 K
D1 1.00000000 sec
TD0 1

======== CHANNEL f1 ========
SFO1 400.2124715 MHz
NUC1 1H
P1 15.54 usec
PLW1 12.00000000 W

F2 - Processing parameters
SI 65536
SF 400.2100000 MHz
WDW EM
SSB 0
LB 0.30 Hz
GB 0
PC 1.00

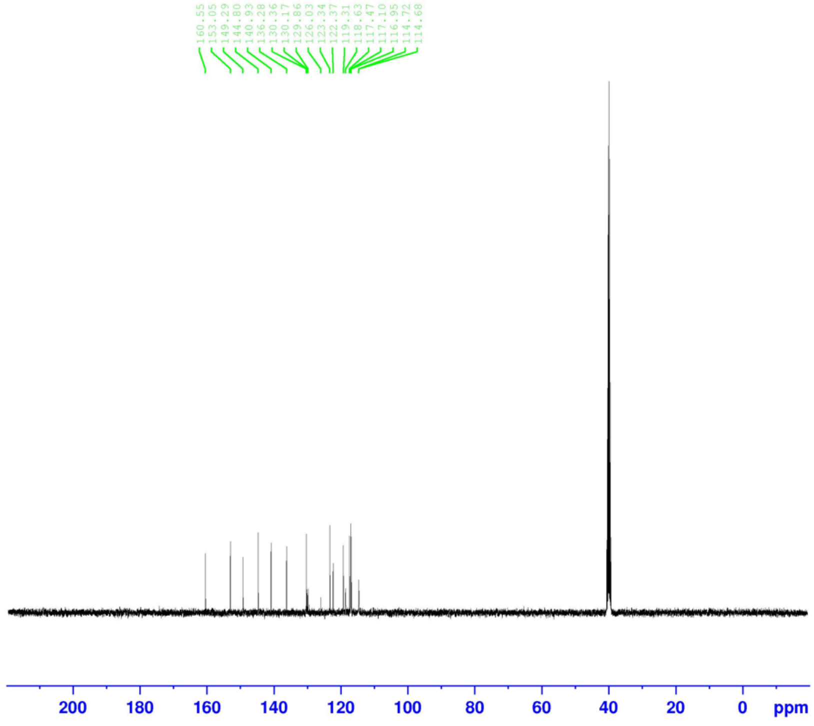

Current Data Parameters
NAME AKA-VII-81
EXPNO 2
PROCNO 1

F2 - Acquisition Parameters
Date_ 20201008
Time 18.11
INSTRUM spect
PROBHD 5 mm PABBO BB/
PULPROG zgpg30
TD 65536
SOLVENT DMSO
NS 100
DS 4
SWH 24038.461 Hz
FIDRES 0.366798 Hz
AQ 1.3631488 sec
RG 190.62
DW 20.800 usec
DE 6.50 usec
TE 298.5 K
D1 2.00000000 sec
D11 0.03000000 sec
TD0 1

======== CHANNEL f1 ========
SFO1 100.6429474 MHz
NUC1 13C
P1 10.00 usec
PLW1 52.00000000 W

======== CHANNEL f2 ========
SFO2 400.2116008 MHz
NUC2 1H
CPDPRG[2 waltz16
PCPD2 90.00 usec
PLW2 12.00000000 W
PLW12 0.35777000 W
PLW13 0.28979000 W

F2 - Processing parameters
SI 32768
SF 100.6328850 MHz
WDW EM
SSB 0
LB 1.00 Hz
GB 0
PC 1.40

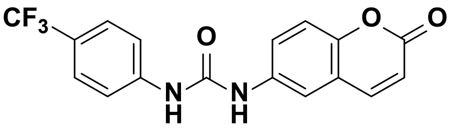
CF3
O
N
H
N
H
O
O

5b

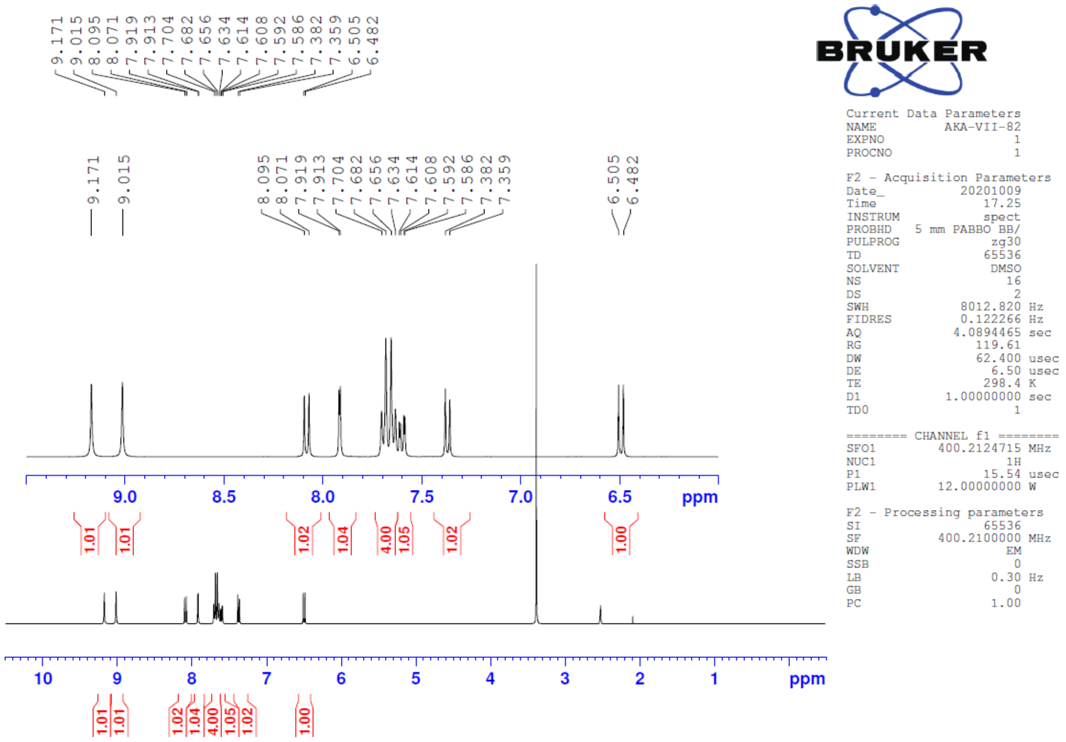
9.171
9.015
8.095
8.071
7.919
7.913
7.704
7.682
7.656
7.634
7.614
7.608
7.592
7.586
7.382
7.359
6.505
6.482
9.0
8.5
8.0
7.5
7.0
6.5
ppm
1.01
1.01
1.02
1.04
4.00
1.05
1.02
1.00
10
9
8
7
6
5
4
3
2
1
ppm
BRUKER
Current Data Parameters
NAME AKA-VII-82
EXPNO 1
PROCNO 1
F2 - Acquisition Parameters
Date_ 20201009
Time 17.25
INSTRUM spect
PROBHD 5 mm PABBO BB/
PULPROG zg30
TD 65536
SOLVENT DMSO
NS 16
DS 2
SWH 8012.820 Hz
FIDRES 0.122266 Hz
AQ 4.0894465 sec
RG 119.61
DW 62.400 usec
DE 6.50 usec
TE 298.4 K
D1 1.00000000 sec
TD0 1
======== CHANNEL f1 ========
SFO1 400.2124715 MHz
NUC1 1H
P1 15.54 usec
PLW1 12.00000000 W
F2 - Processing parameters
SI 65536
SF 400.2100000 MHz
WDW EM
SSB 0
LB 0.30 Hz
GB 0
PC 1.00

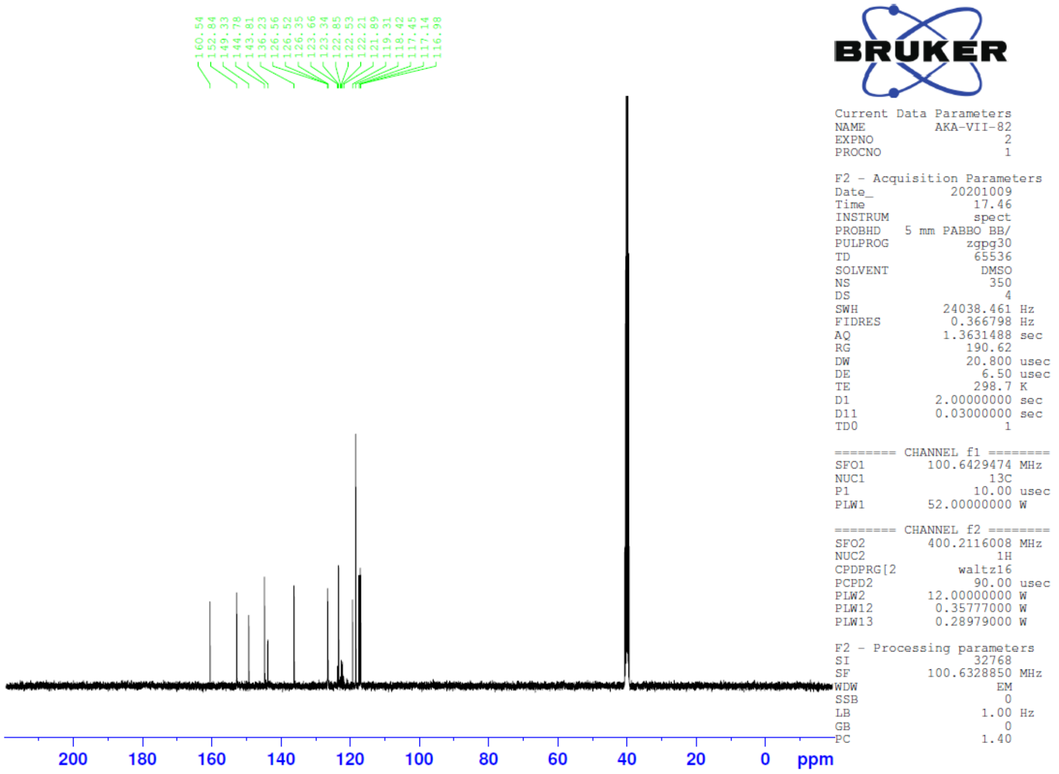
160.54
152.84
149.33
144.78
143.81
136.23
126.56
126.52
126.35
123.66
123.34
122.85
122.53
122.21
121.89
119.31
118.42
117.45
117.14
116.98
200
180
160
140
120
100
80
60
40
20
0
ppm
BRUKER
Current Data Parameters
NAME AKA-VII-82
EXPNO 2
PROCNO 1
F2 - Acquisition Parameters
Date_ 20201009
Time 17.46
INSTRUM spect
PROBHD 5 mm PABBO BB/
PULPROG zgpg30
TD 65536
SOLVENT DMSO
NS 350
DS 4
SWH 24038.461 Hz
FIDRES 0.366798 Hz
AQ 1.3631488 sec
RG 190.62
DW 20.800 usec
DE 6.50 usec
TE 298.7 K
D1 2.00000000 sec
D11 0.03000000 sec
TD0 1
======== CHANNEL f1 ========
SFO1 100.6429474 MHz
NUC1 13C
P1 10.00 usec
PLW1 52.00000000 W
======== CHANNEL f2 ========
SFO2 400.2116008 MHz
NUC2 1H
CPDPRG[2 waltz16
PCPD2 90.00 usec
PLW2 12.00000000 W
PLW12 0.35777000 W
PLW13 0.28979000 W
F2 - Processing parameters
SI 32768
SF 100.6328850 MHz
WDW EM
SSB 0
LB 1.00 Hz
GB 0
PC 1.40

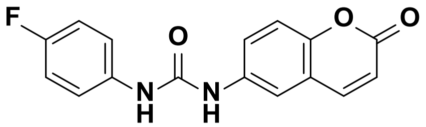

F
O
N
H
N
H
O
O

5c

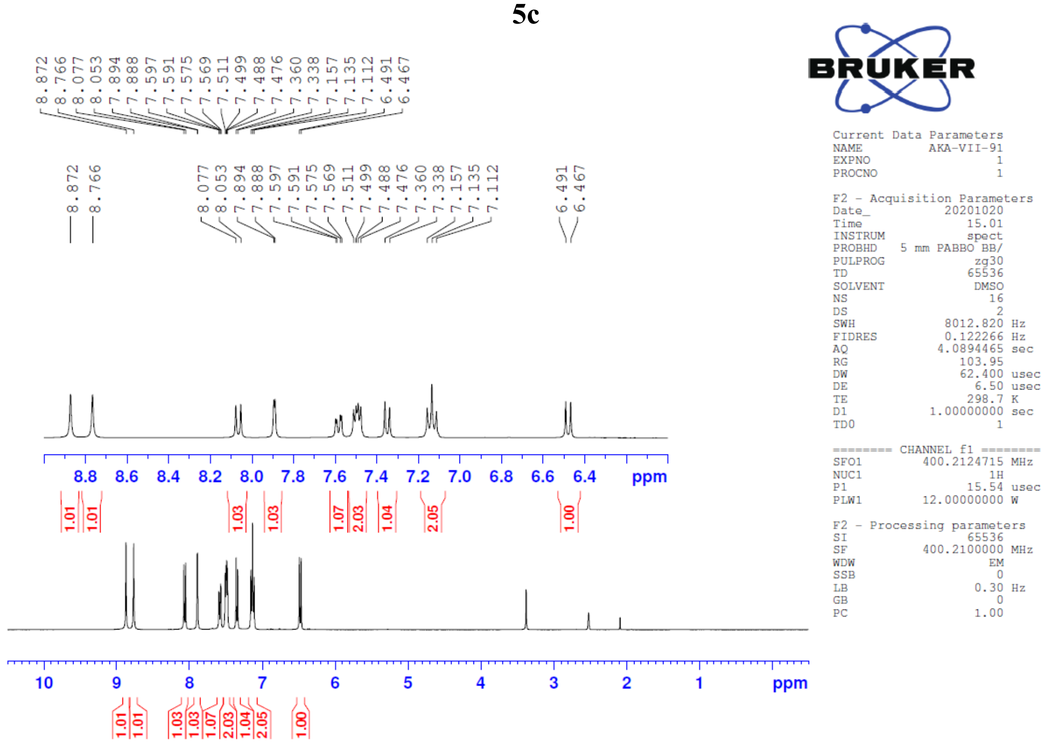

8.872
8.766
8.077
8.053
7.894
7.888
7.597
7.591
7.575
7.569
7.511
7.499
7.488
7.476
7.360
7.338
7.157
7.135
7.112
6.491
6.467
BRUKER
Current Data Parameters
NAME AKA-VII-91
EXPNO 1
PROCNO 1
F2 - Acquisition Parameters
Date_ 20201020
Time 15.01
INSTRUM spect
PROBHD 5 mm PABBO BB/
PULPROG zg30
TD 65536
SOLVENT DMSO
NS 16
DS 2
SWH 8012.820 Hz
FIDRES 0.122266 Hz
AQ 4.0894465 sec
RG 103.95
DW 62.400 usec
DE 6.50 usec
TE 298.7 K
D1 1.00000000 sec
TD0 1
======== CHANNEL f1 ========
SFO1 400.2124715 MHz
NUC1 1H
P1 15.54 usec
PLW1 12.00000000 W
F2 - Processing parameters
SI 65536
SF 400.2100000 MHz
WDW EM
SSB 0
LB 0.30 Hz
GB 0
PC 1.00
1.01 1.01 1.03 1.03 1.07 2.03 1.04 2.05 1.00
ppm

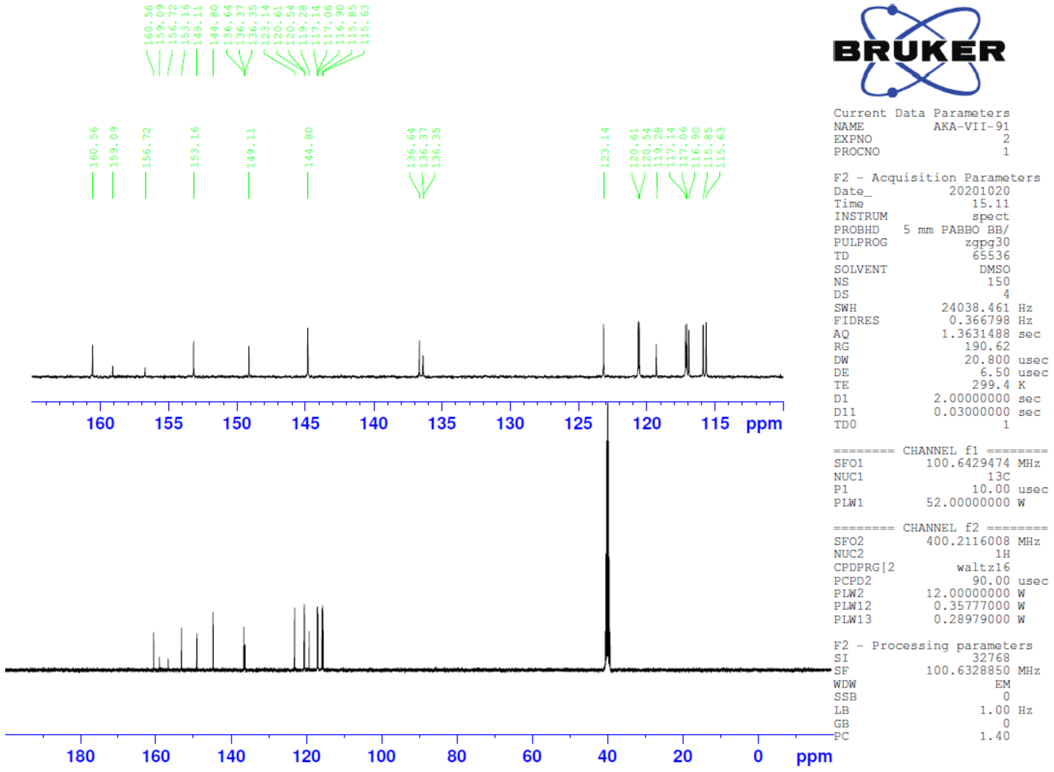

160.56
159.09
156.72
153.16
149.11
144.80
136.64
136.37
136.35
123.14
120.61
120.54
119.28
117.14
117.06
116.90
115.85
115.63
BRUKER
Current Data Parameters
NAME AKA-VII-91
EXPNO 2
PROCNO 1
F2 - Acquisition Parameters
Date_ 20201020
Time 15.11
INSTRUM spect
PROBHD 5 mm PABBO BB/
PULPROG zgpg30
TD 65536
SOLVENT DMSO
NS 150
DS 4
SWH 24038.461 Hz
FIDRES 0.366798 Hz
AQ 1.3631488 sec
RG 190.62
DW 20.800 usec
DE 6.50 usec
TE 299.4 K
D1 2.00000000 sec
D11 0.03000000 sec
TD0 1
======== CHANNEL f1 ========
SFO1 100.6429474 MHz
NUC1 13C
P1 10.00 usec
PLW1 52.00000000 W
======== CHANNEL f2 ========
SFO2 400.2116008 MHz
NUC2 1H
CPDPRG[2 waltz16
PCPD2 90.00 usec
PLW2 12.00000000 W
PLW12 0.35777000 W
PLW13 0.28979000 W
F2 - Processing parameters
SI 32768
SF 100.6328850 MHz
WDW EM
SSB 0
LB 1.00 Hz
GB 0
PC 1.40
ppm

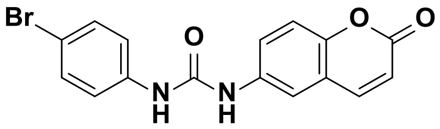
Br
O
N
H
N
H
O
O

5d

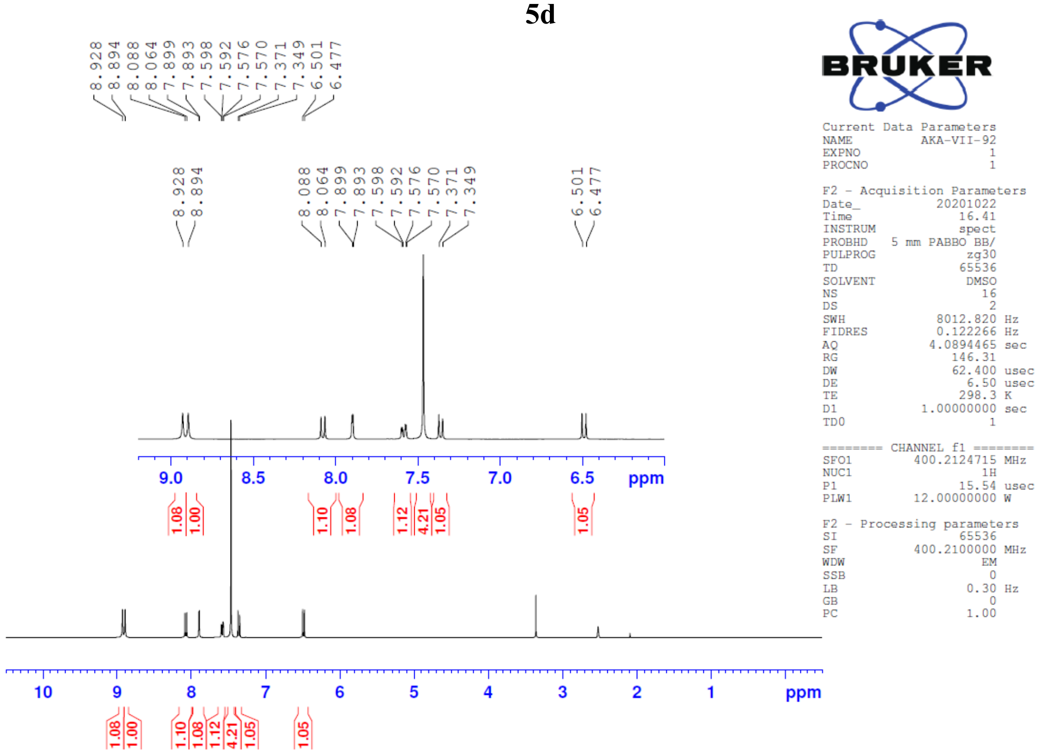
8.928
8.894
8.088
8.064
7.899
7.893
7.598
7.592
7.576
7.570
7.371
7.349
6.501
6.477
9.0
8.5
8.0
7.5
7.0
6.5
ppm
1.08
1.00
1.10
1.08
1.12
4.21
1.05
1.05
10
9
8
7
6
5
4
3
2
1
ppm
Current Data Parameters
NAME AKA-VII-92
EXPNO 1
PROCNO 1
F2 - Acquisition Parameters
Date_ 20201022
Time 16.41
INSTRUM spect
PROBHD 5 mm PABBO BB/
PULPROG zg30
TD 65536
SOLVENT DMSO
NS 16
DS 2
SWH 8012.820 Hz
FIDRES 0.122266 Hz
AQ 4.0894465 sec
RG 146.31
DW 62.400 usec
DE 6.50 usec
TE 298.3 K
D1 1.00000000 sec
TD0 1
======== CHANNEL f1 ========
SFO1 400.2124715 MHz
NUC1 1H
P1 15.54 usec
PLW1 12.00000000 W
F2 - Processing parameters
SI 65536
SF 400.2100000 MHz
WDW EM
SSB 0
LB 0.30 Hz
GB 0
PC 1.00

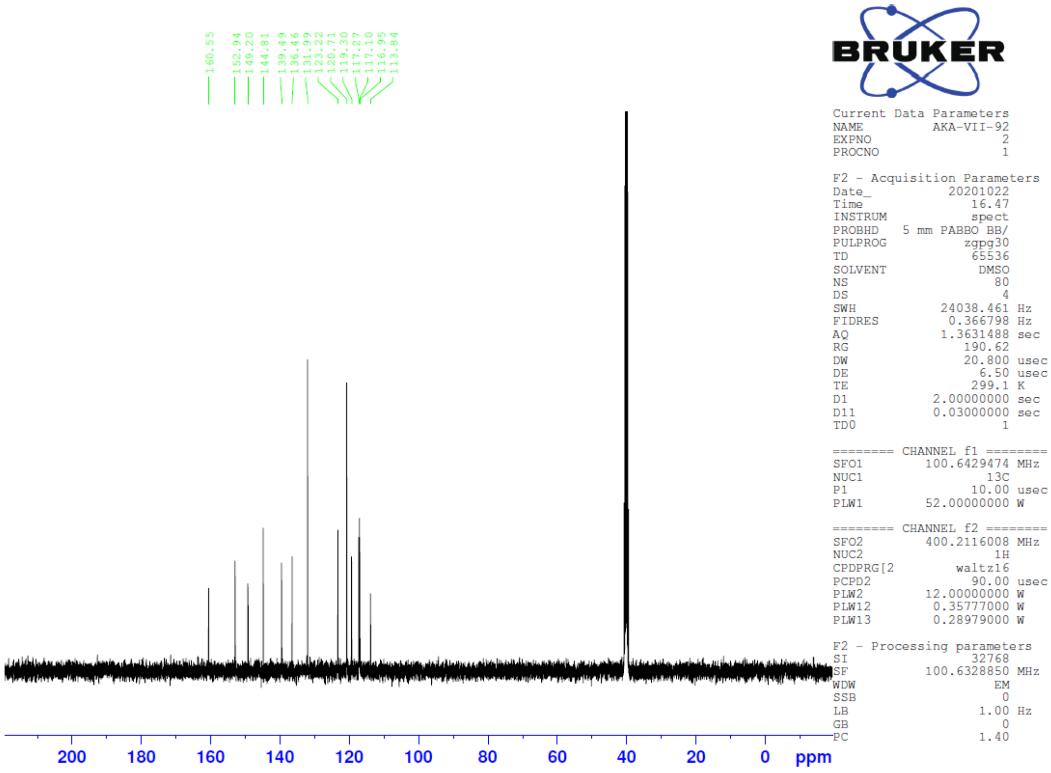
160.55
152.94
149.20
144.81
139.49
136.46
131.99
123.22
120.71
119.30
117.27
117.10
116.95
113.84
200
180
160
140
120
100
80
60
40
20
0
ppm
Current Data Parameters
NAME AKA-VII-92
EXPNO 2
PROCNO 1
F2 - Acquisition Parameters
Date_ 20201022
Time 16.47
INSTRUM spect
PROBHD 5 mm PABBO BB/
PULPROG zgpg30
TD 65536
SOLVENT DMSO
NS 80
DS 4
SWH 24038.461 Hz
FIDRES 0.366798 Hz
AQ 1.3631488 sec
RG 190.62
DW 20.800 usec
DE 6.50 usec
TE 299.1 K
D1 2.00000000 sec
D11 0.03000000 sec
TD0 1
======== CHANNEL f1 ========
SFO1 100.6429474 MHz
NUC1 13C
P1 10.00 usec
PLW1 52.00000000 W
======== CHANNEL f2 ========
SFO2 400.2116008 MHz
NUC2 1H
CPDPRG[2 waltz16
PCPD2 90.00 usec
PLW2 12.00000000 W
PLW12 0.35777000 W
PLW13 0.28979000 W
F2 - Processing parameters
SI 32768
SF 100.6328850 MHz
WDW EM
SSB 0
LB 1.00 Hz
GB 0
PC 1.40

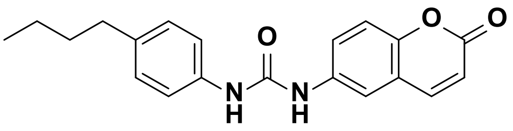
O
O
O
N
H
N
H

5e

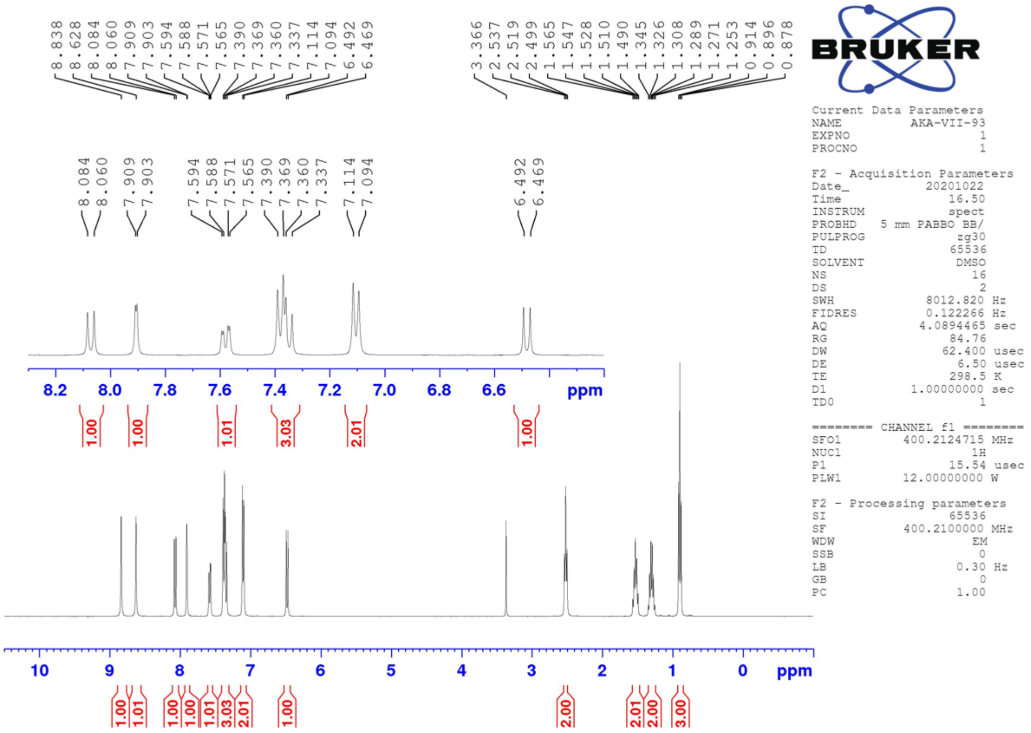
8.838
8.628
8.084
8.060
7.909
7.903
7.594
7.588
7.571
7.565
7.390
7.369
7.360
7.337
7.114
7.094
6.492
6.469
3.366
2.537
2.519
2.499
1.565
1.547
1.528
1.510
1.490
1.345
1.326
1.308
1.289
1.271
1.253
0.914
0.896
0.878
BRUKER
Current Data Parameters
NAME AKA-VII-93
EXPNO 1
PROCNO 1
F2 - Acquisition Parameters
Date_ 20201022
Time 16.50
INSTRUM spect
PROBHD 5 mm PABBO BB/
PULPROG zg30
TD 65536
SOLVENT DMSO
NS 16
DS 2
SWH 8012.820 Hz
FIDRES 0.122266 Hz
AQ 4.0894465 sec
RG 84.76
DW 62.400 usec
DE 6.50 usec
TE 298.5 K
D1 1.00000000 sec
TD0 1
======== CHANNEL f1 ========
SFO1 400.2124715 MHz
NUC1 1H
P1 15.54 usec
PLW1 12.00000000 W
F2 - Processing parameters
SI 65536
SF 400.2100000 MHz
WDW EM
SSB 0
LB 0.30 Hz
GB 0
PC 1.00
8.2 8.0 7.8 7.6 7.4 7.2 7.0 6.8 6.6 ppm
1.00 1.00 1.01 3.03 2.01 1.00
10 9 8 7 6 5 4 3 2 1 0 ppm
1.00 1.01 1.00 1.00 1.01 3.03 2.01 1.00 2.00 2.01 2.00 3.00

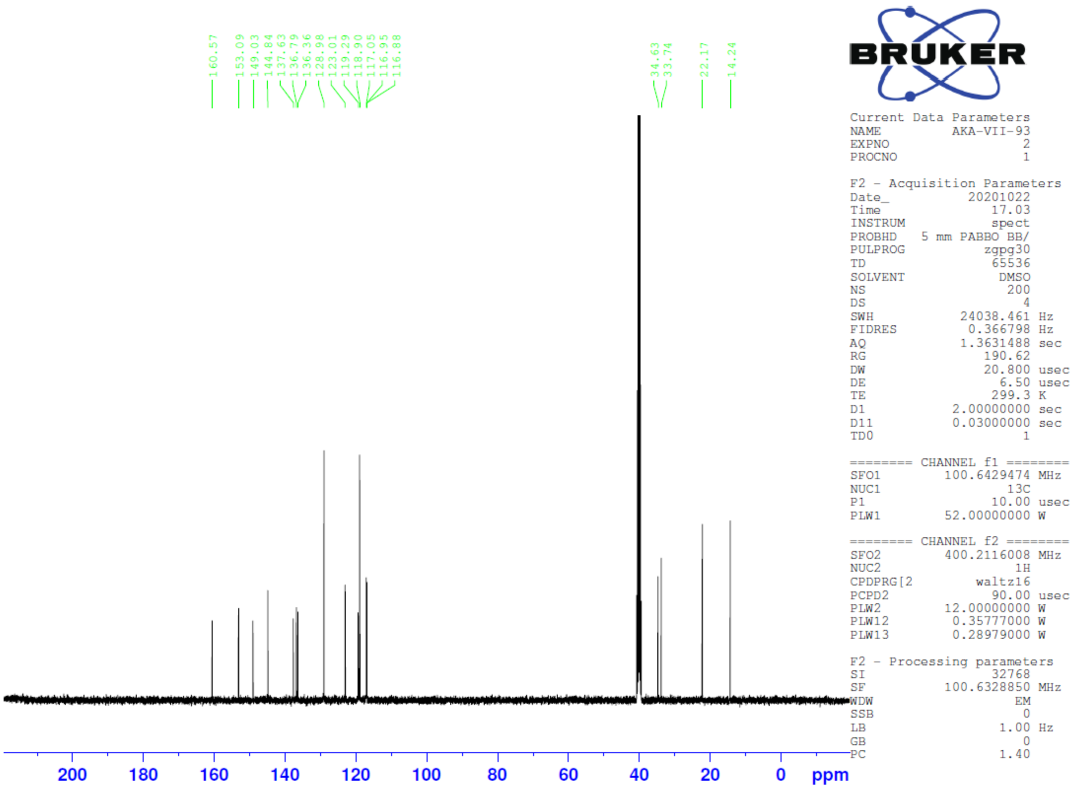
160.57
153.09
149.03
144.84
137.63
136.79
136.36
128.98
123.01
119.29
118.90
117.05
116.95
116.88
34.63
33.74
22.17
14.24
BRUKER
Current Data Parameters
NAME AKA-VII-93
EXPNO 2
PROCNO 1
F2 - Acquisition Parameters
Date_ 20201022
Time 17.03
INSTRUM spect
PROBHD 5 mm PABBO BB/
PULPROG zgpg30
TD 65536
SOLVENT DMSO
NS 200
DS 4
SWH 24038.461 Hz
FIDRES 0.366798 Hz
AQ 1.3631488 sec
RG 190.62
DW 20.800 usec
DE 6.50 usec
TE 299.3 K
D1 2.00000000 sec
D11 0.03000000 sec
TD0 1
======== CHANNEL f1 ========
SFO1 100.6429474 MHz
NUC1 13C
P1 10.00 usec
PLW1 52.00000000 W
======== CHANNEL f2 ========
SFO2 400.2116008 MHz
NUC2 1H
CPDPRG[2 waltz16
PCPD2 90.00 usec
PLW2 12.00000000 W
PLW12 0.35777000 W
PLW13 0.28979000 W
F2 - Processing parameters
SI 32768
SF 100.6328850 MHz
WDW EM
SSB 0
LB 1.00 Hz
GB 0
PC 1.40
200 180 160 140 120 100 80 60 40 20 0 ppm

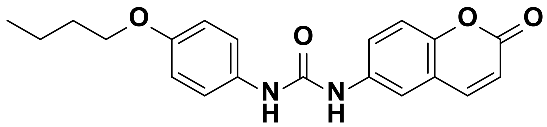
O
O
O
O
N
H
N
H

5f

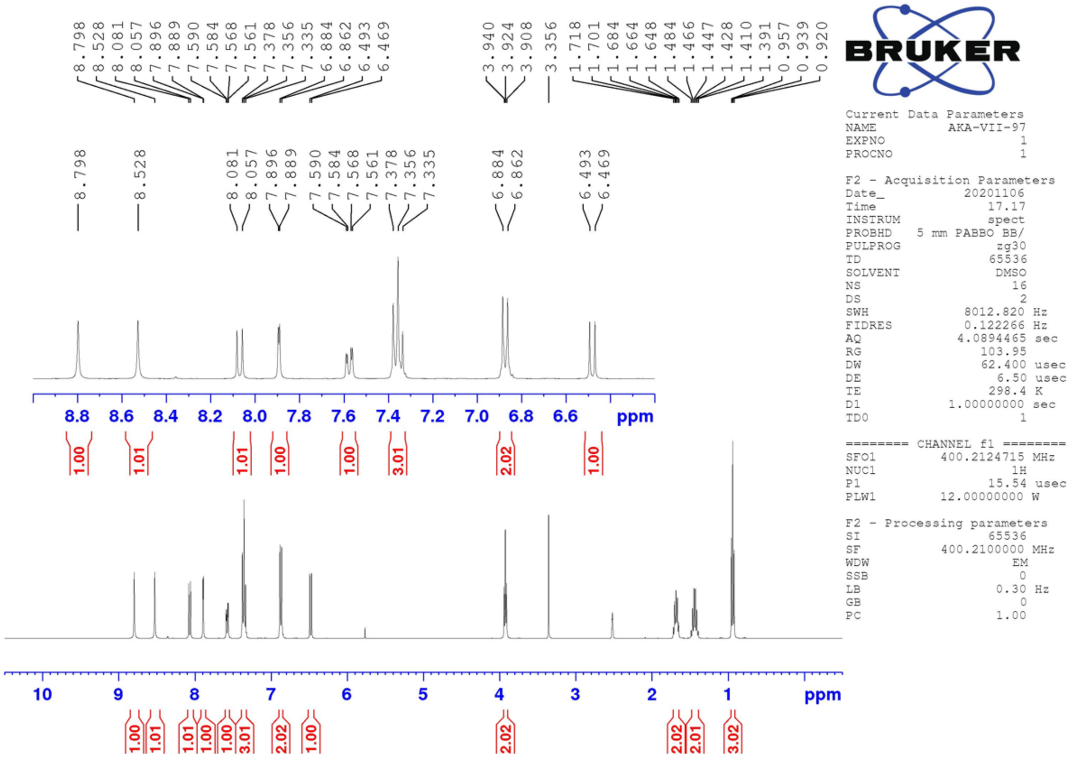
BRUKER
Current Data Parameters
NAME AKA-VII-97
EXPNO 1
PROCNO 1
F2 - Acquisition Parameters
Date_ 20201106
Time 17.17
INSTRUM spect
PROBHD 5 mm PABBO BB/
PULPROG zg30
TD 65536
SOLVENT DMSO
NS 16
DS 2
SWH 8012.820 Hz
FIDRES 0.122266 Hz
AQ 4.0894465 sec
RG 103.95
DW 62.400 usec
DE 6.50 usec
TE 298.4 K
D1 1.00000000 sec
TD0 1
======== CHANNEL f1 ========
SFO1 400.2124715 MHz
NUC1 1H
P1 15.54 usec
PLW1 12.00000000 W
F2 - Processing parameters
SI 65536
SF 400.2100000 MHz
WDW EM
SSB 0
LB 0.30 Hz
GB 0
PC 1.00
ppm

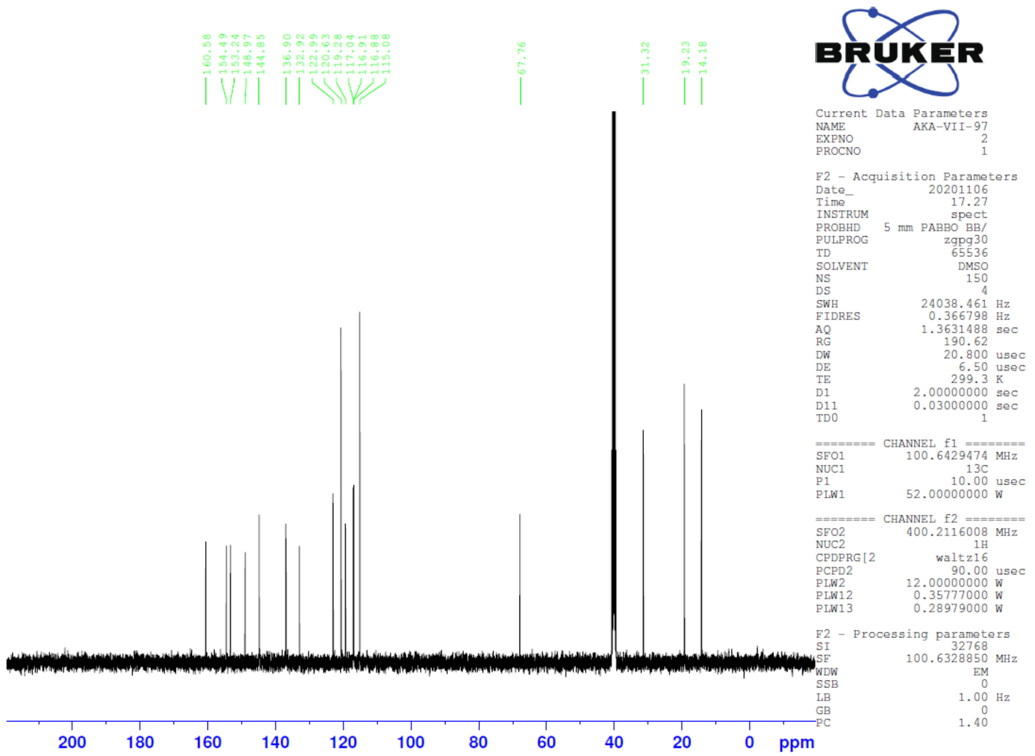
160.58
154.49
153.24
148.97
144.85
136.90
132.92
122.99
120.63
119.28
117.04
116.91
116.88
115.08
67.76
31.32
19.23
14.18
BRUKER
Current Data Parameters
NAME AKA-VII-97
EXPNO 2
PROCNO 1
F2 - Acquisition Parameters
Date_ 20201106
Time 17.27
INSTRUM spect
PROBHD 5 mm PABBO BB/
PULPROG zgpg30
TD 65536
SOLVENT DMSO
NS 150
DS 4
SWH 24038.461 Hz
FIDRES 0.366798 Hz
AQ 1.3631488 sec
RG 190.62
DW 20.800 usec
DE 6.50 usec
TE 299.3 K
D1 2.00000000 sec
D11 0.03000000 sec
TD0 1
======== CHANNEL f1 ========
SFO1 100.6429474 MHz
NUC1 13C
P1 10.00 usec
PLW1 52.00000000 W
======== CHANNEL f2 ========
SFO2 400.2116008 MHz
NUC2 1H
CPDPRG[2 waltz16
PCPD2 90.00 usec
PLW2 12.00000000 W
PLW12 0.35777000 W
PLW13 0.28979000 W
F2 - Processing parameters
SI 32768
SF 100.6328850 MHz
WDW EM
SSB 0
LB 1.00 Hz
GB 0
PC 1.40
ppm

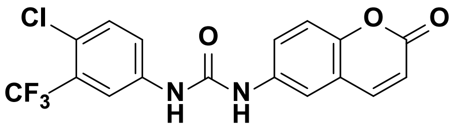
Cl
CF3
O
N
H
N
H
O
O

5g

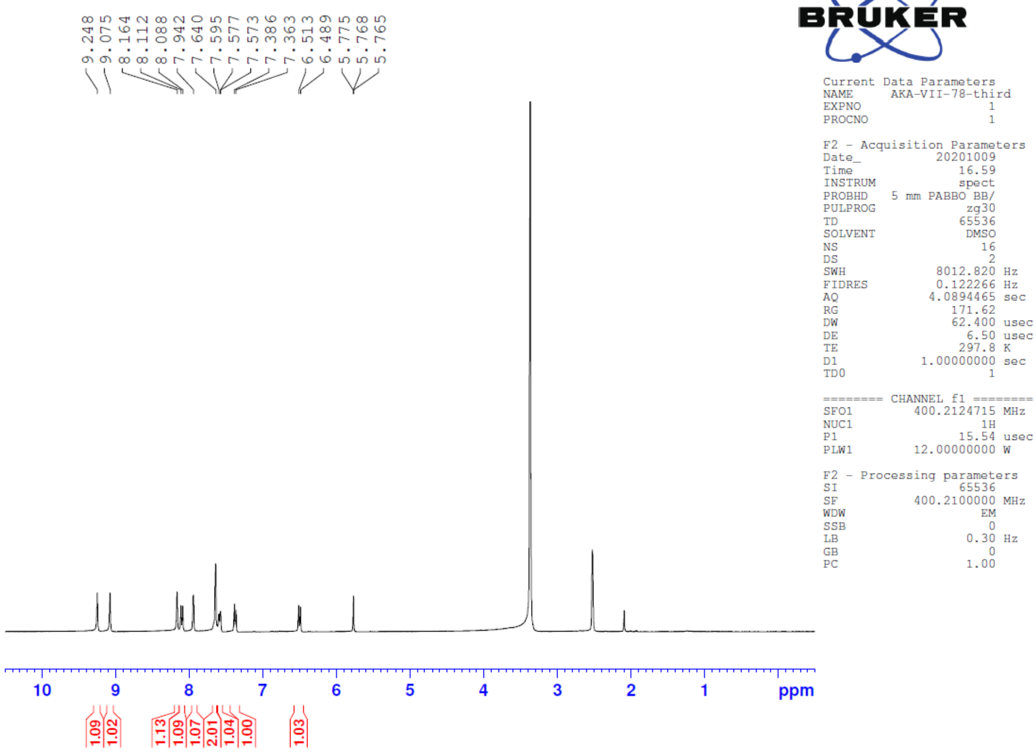
9.248
9.075
8.164
8.112
8.088
7.942
7.640
7.595
7.577
7.573
7.386
7.363
6.513
6.489
5.775
5.768
5.765
BRUKER
Current Data Parameters
NAME AKA-VII-78-third
EXPNO 1
PROCNO 1
F2 - Acquisition Parameters
Date_ 20201009
Time 16.59
INSTRUM spect
PROBHD 5 mm PABBO BB/
PULPROG zg30
TD 65536
SOLVENT DMSO
NS 16
DS 2
SWH 8012.820 Hz
FIDRES 0.122266 Hz
AQ 4.0894465 sec
RG 171.62
DW 62.400 usec
DE 6.50 usec
TE 297.8 K
D1 1.00000000 sec
TD0 1
======== CHANNEL f1 ========
SFO1 400.2124715 MHz
NUC1 1H
P1 15.54 usec
PLW1 12.00000000 W
F2 - Processing parameters
SI 65536
SF 400.2100000 MHz
WDW EM
SSB 0
LB 0.30 Hz
GB 0
PC 1.00
10 9 8 7 6 5 4 3 2 1 ppm
1.09
1.02
1.13
1.09
1.07
2.01
1.04
1.00
1.03

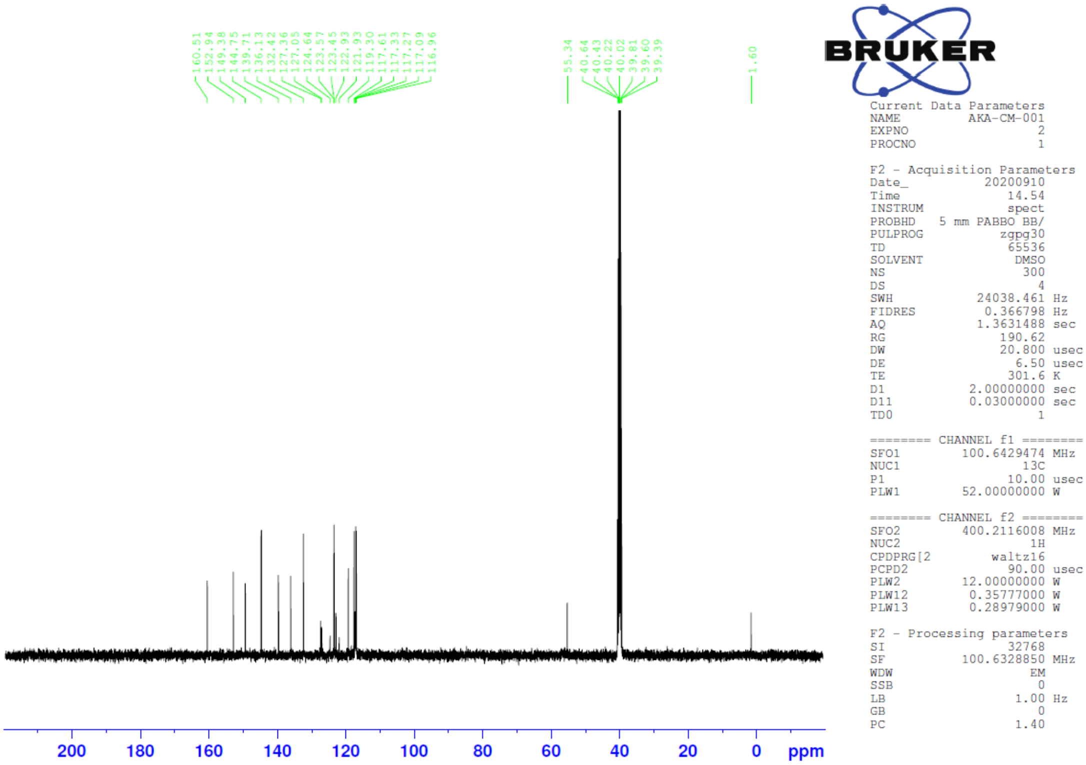
160.51
152.94
149.38
144.75
139.71
136.13
132.42
127.05
124.64
123.57
123.45
122.93
121.93
119.30
117.61
117.33
117.27
117.09
116.96
55.34
40.64
40.43
40.22
40.02
39.81
39.60
39.39
1.60
BRUKER
Current Data Parameters
NAME AKA-CM-001
EXPNO 2
PROCNO 1
F2 - Acquisition Parameters
Date_ 20200910
Time 14.54
INSTRUM spect
PROBHD 5 mm PABBO BB/
PULPROG zgpg30
TD 65536
SOLVENT DMSO
NS 300
DS 4
SWH 24038.461 Hz
FIDRES 0.366798 Hz
AQ 1.3631488 sec
RG 190.62
DW 20.800 usec
DE 6.50 usec
TE 301.6 K
D1 2.00000000 sec
D11 0.03000000 sec
TD0 1
======== CHANNEL f1 ========
SFO1 100.6429474 MHz
NUC1 13C
P1 10.00 usec
PLW1 52.00000000 W
======== CHANNEL f2 ========
SFO2 400.2116008 MHz
NUC2 1H
CPDPRG[2 waltz16
PCPD2 90.00 usec
PLW2 12.00000000 W
PLW12 0.35777000 W
PLW13 0.28979000 W
F2 - Processing parameters
SI 32768
SF 100.6328850 MHz
WDW EM
SSB 0
LB 1.00 Hz
GB 0
PC 1.40
200 180 160 140 120 100 80 60 40 20 0 ppm

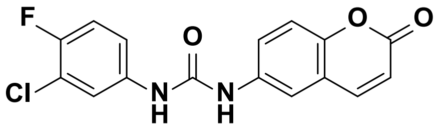
F
Cl
O
N
H
N
H
O
O

5h

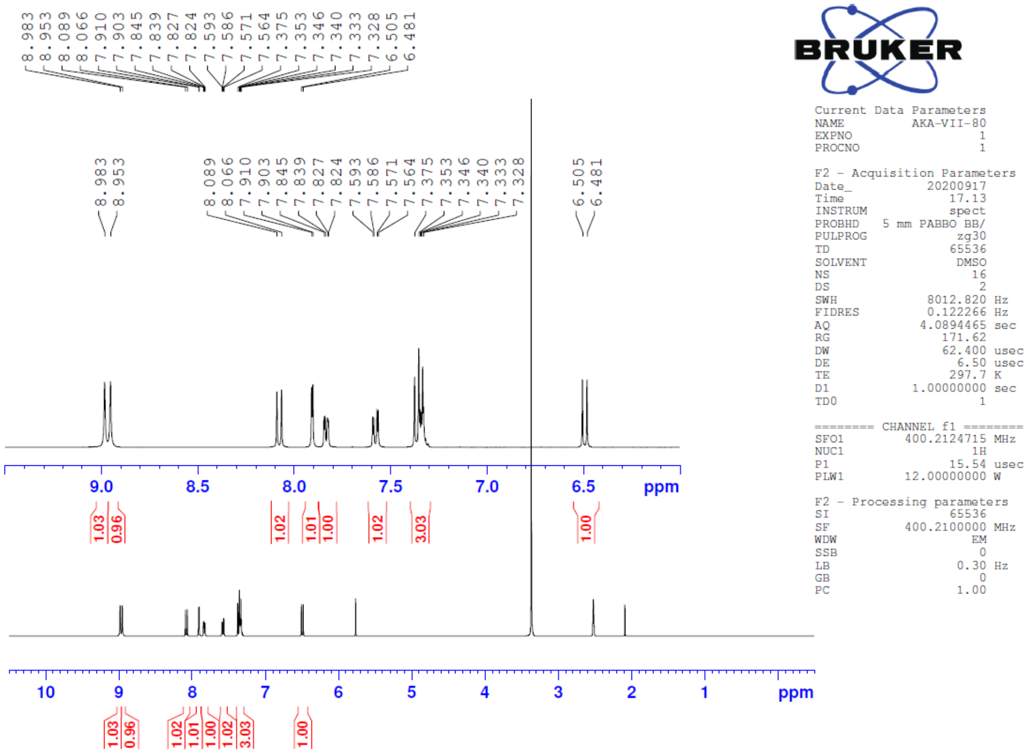
8.983
8.953
8.089
8.066
7.910
7.903
7.845
7.839
7.827
7.824
7.593
7.586
7.571
7.564
7.375
7.353
7.346
7.340
7.333
7.328
6.505
6.481
9.0
8.5
8.0
7.5
7.0
6.5
ppm
1.03
0.96
1.02
1.01
1.00
1.02
3.03
1.00
10
9
8
7
6
5
4
3
2
1
ppm
BRUKER
Current Data Parameters
NAME AKA-VII-80
EXPNO 1
PROCNO 1
F2 - Acquisition Parameters
Date_ 20200917
Time 17.13
INSTRUM spect
PROBHD 5 mm PABBO BB/
PULPROG zg30
TD 65536
SOLVENT DMSO
NS 16
DS 2
SWH 8012.820 Hz
FIDRES 0.122266 Hz
AQ 4.0894465 sec
RG 171.62
DW 62.400 usec
DE 6.50 usec
TE 297.7 K
D1 1.00000000 sec
TD0 1
======== CHANNEL f1 ========
SFO1 400.2124715 MHz
NUC1 1H
P1 15.54 usec
PLW1 12.00000000 W
F2 - Processing parameters
SI 65536
SF 400.2100000 MHz
WDW EM
SSB 0
LB 0.30 Hz
GB 0
PC 1.00

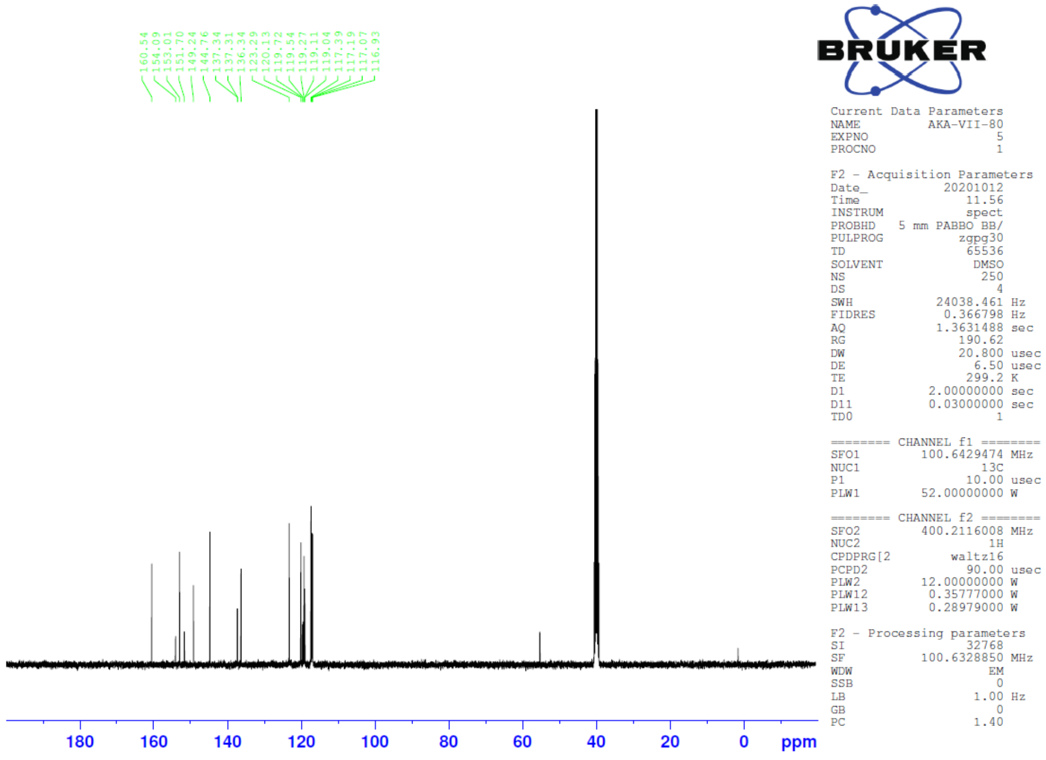
160.54
154.09
153.01
151.70
149.24
144.76
137.34
137.31
136.34
123.29
120.13
119.54
119.27
119.11
119.04
117.39
117.19
117.07
116.93
180
160
140
120
100
80
60
40
20
0
ppm
BRUKER
Current Data Parameters
NAME AKA-VII-80
EXPNO 5
PROCNO 1
F2 - Acquisition Parameters
Date_ 20201012
Time 11.56
INSTRUM spect
PROBHD 5 mm PABBO BB/
PULPROG zgpg30
TD 65536
SOLVENT DMSO
NS 250
DS 4
SWH 24038.461 Hz
FIDRES 0.366798 Hz
AQ 1.3631488 sec
RG 190.62
DW 20.800 usec
DE 6.50 usec
TE 299.2 K
D1 2.00000000 sec
D11 0.03000000 sec
TD0 1
======== CHANNEL f1 ========
SFO1 100.6429474 MHz
NUC1 13C
P1 10.00 usec
PLW1 52.00000000 W
======== CHANNEL f2 ========
SFO2 400.2116008 MHz
NUC2 1H
CPDPRG[2 waltz16
PCPD2 90.00 usec
PLW2 12.00000000 W
PLW12 0.35777000 W
PLW13 0.28979000 W
F2 - Processing parameters
SI 32768
SF 100.6328850 MHz
WDW EM
SSB 0
LB 1.00 Hz
GB 0
PC 1.40

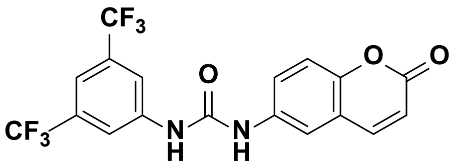
CF3
CF3
O
N
H
N
H
O
O

5i

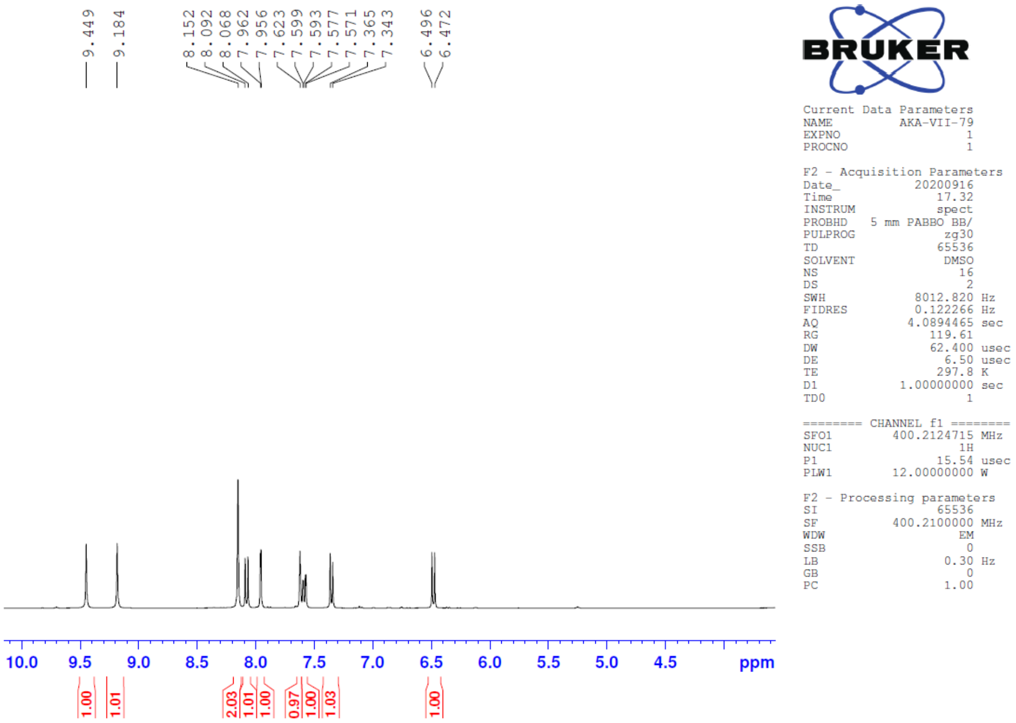
9.449
9.184
8.152
8.092
8.068
7.962
7.956
7.623
7.599
7.593
7.577
7.571
7.365
7.343
6.496
6.472
Current Data Parameters
NAME AKA-VII-79
EXPNO 1
PROCNO 1
F2 - Acquisition Parameters
Date_ 20200916
Time 17.32
INSTRUM spect
PROBHD 5 mm PABBO BB/
PULPROG zg30
TD 65536
SOLVENT DMSO
NS 16
DS 2
SWH 8012.820 Hz
FIDRES 0.122266 Hz
AQ 4.0894465 sec
RG 119.61
DW 62.400 usec
DE 6.50 usec
TE 297.8 K
D1 1.00000000 sec
TD0 1
======== CHANNEL f1 ========
SFO1 400.2124715 MHz
NUC1 1H
P1 15.54 usec
PLW1 12.00000000 W
F2 - Processing parameters
SI 65536
SF 400.2100000 MHz
WDW EM
SSB 0
LB 0.30 Hz
GB 0
PC 1.00
10.0
9.5
9.0
8.5
8.0
7.5
7.0
6.5
6.0
5.5
5.0
4.5
ppm
1.00
1.01
2.03
1.01
1.00
0.97
1.00
1.03
1.00

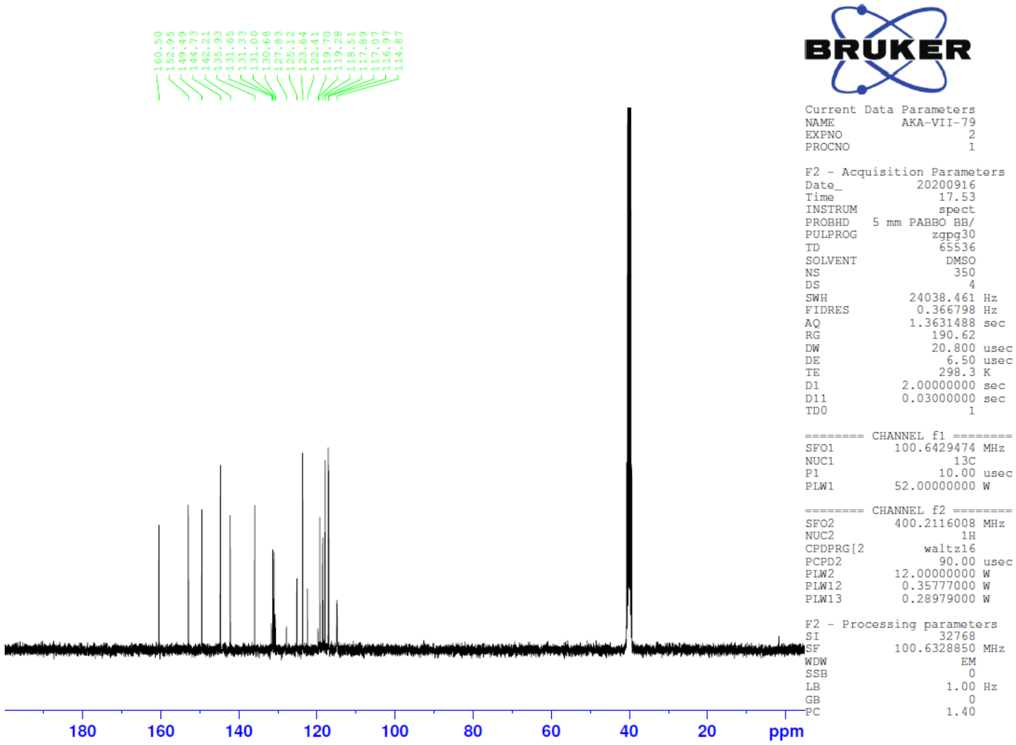
160.50
152.95
149.49
144.73
142.21
135.93
131.65
131.33
131.00
130.68
127.83
125.12
123.64
122.41
119.70
119.28
118.51
117.89
117.07
116.97
114.87
Current Data Parameters
NAME AKA-VII-79
EXPNO 2
PROCNO 1
F2 - Acquisition Parameters
Date_ 20200916
Time 17.53
INSTRUM spect
PROBHD 5 mm PABBO BB/
PULPROG zgpg30
TD 65536
SOLVENT DMSO
NS 350
DS 4
SWH 24038.461 Hz
FIDRES 0.366798 Hz
AQ 1.3631488 sec
RG 190.62
DW 20.800 usec
DE 6.50 usec
TE 298.3 K
D1 2.00000000 sec
D11 0.03000000 sec
TD0 1
======== CHANNEL f1 ========
SFO1 100.6429474 MHz
NUC1 13C
P1 10.00 usec
PLW1 52.00000000 W
======== CHANNEL f2 ========
SFO2 400.2116008 MHz
NUC2 1H
CPDPRG[2 waltz16
PCPD2 90.00 usec
PLW2 12.00000000 W
PLW12 0.35777000 W
PLW13 0.28979000 W
F2 - Processing parameters
SI 32768
SF 100.6328850 MHz
WDW EM
SSB 0
LB 1.00 Hz
GB 0
PC 1.40
180
160
140
120
100
80
60
40
20
ppm

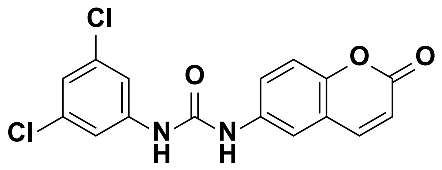

Cl
Cl
O
N
H
N
H
O
O

5j

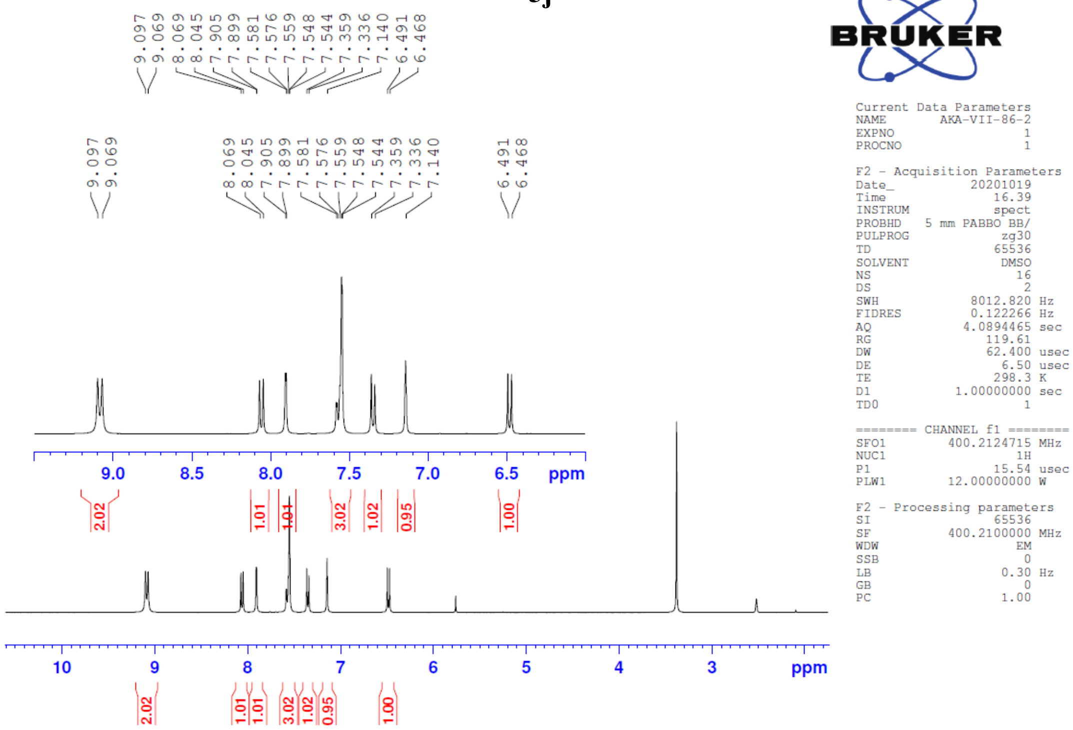

BRUKER
Current Data Parameters
NAME AKA-VII-86-2
EXPNO 1
PROCNO 1
F2 - Acquisition Parameters
Date_ 20201019
Time 16.39
INSTRUM spect
PROBHD 5 mm PABBO BB/
PULPROG zg30
TD 65536
SOLVENT DMSO
NS 16
DS 2
SWH 8012.820 Hz
FIDRES 0.122266 Hz
AQ 4.0894465 sec
RG 119.61
DW 62.400 usec
DE 6.50 usec
TE 298.3 K
D1 1.00000000 sec
TD0 1
======== CHANNEL f1 ========
SFO1 400.2124715 MHz
NUC1 1H
P1 15.54 usec
PLW1 12.00000000 W
F2 - Processing parameters
SI 65536
SF 400.2100000 MHz
WDW EM
SSB 0
LB 0.30 Hz
GB 0
PC 1.00
9.097
9.069
8.069
8.045
7.905
7.899
7.581
7.576
7.559
7.548
7.544
7.359
7.336
7.140
6.491
6.468
2.02
1.01
1.01
3.02
1.02
0.95
1.00
ppm

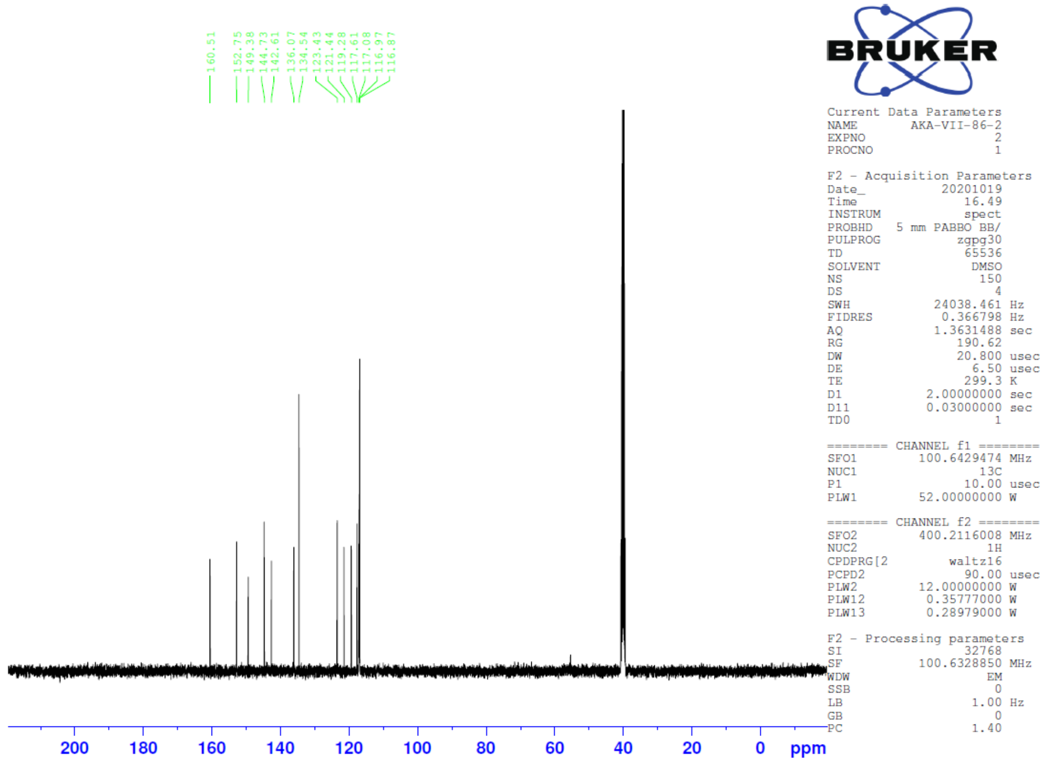

BRUKER
Current Data Parameters
NAME AKA-VII-86-2
EXPNO 2
PROCNO 1
F2 - Acquisition Parameters
Date_ 20201019
Time 16.49
INSTRUM spect
PROBHD 5 mm PABBO BB/
PULPROG zgpg30
TD 65536
SOLVENT DMSO
NS 150
DS 4
SWH 24038.461 Hz
FIDRES 0.366798 Hz
AQ 1.3631488 sec
RG 190.62
DW 20.800 usec
DE 6.50 usec
TE 299.3 K
D1 2.00000000 sec
D11 0.03000000 sec
TD0 1
======== CHANNEL f1 ========
SFO1 100.6429474 MHz
NUC1 13C
P1 10.00 usec
PLW1 52.00000000 W
======== CHANNEL f2 ========
SFO2 400.2116008 MHz
NUC2 1H
CPDPRG[2 waltz16
PCPD2 90.00 usec
PLW2 12.00000000 W
PLW12 0.35777000 W
PLW13 0.28979000 W
F2 - Processing parameters
SI 32768
SF 100.6328850 MHz
WDW EM
SSB 0
LB 1.00 Hz
GB 0
PC 1.40
160.51
152.75
149.38
144.73
142.61
136.07
134.54
123.43
121.44
119.28
117.61
117.08
116.97
116.87
ppm

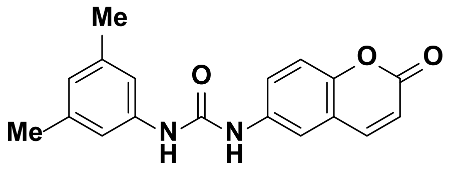
Me
Me
O
N
H
N
H
O
O

5k

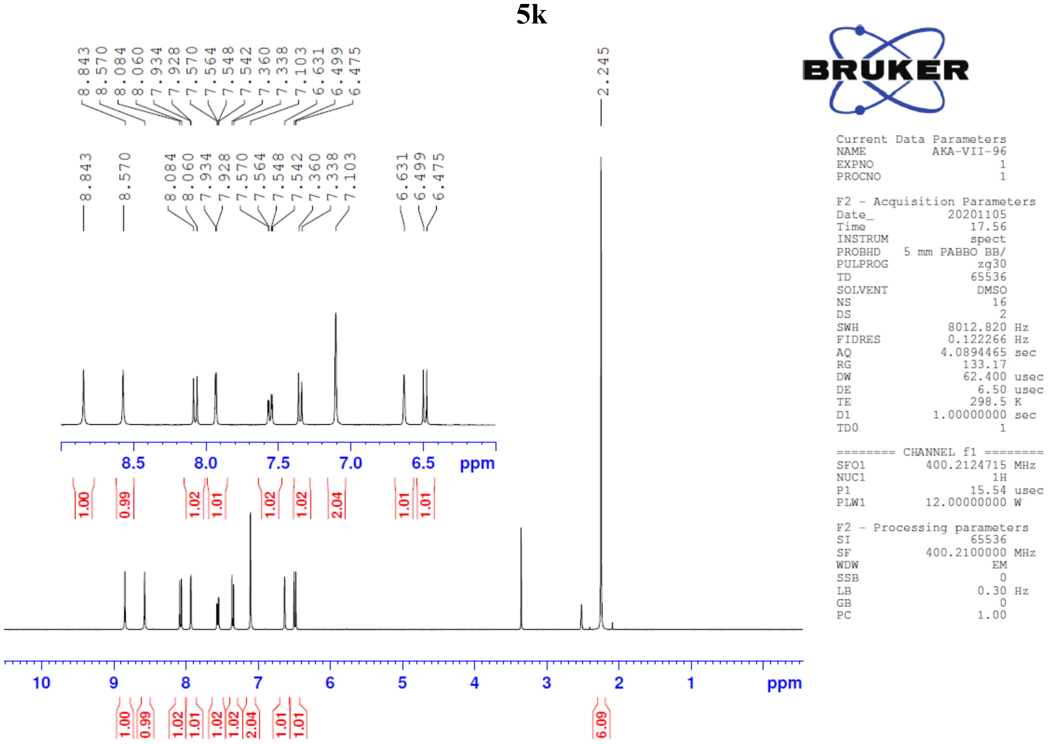
8.843
8.570
8.084
8.060
7.934
7.928
7.570
7.564
7.548
7.542
7.360
7.338
7.103
6.631
6.499
6.475
2.245
8.5
8.0
7.5
7.0
6.5
ppm
1.00
0.99
1.02
1.01
1.02
1.02
2.04
1.01
1.01
10
9
8
7
6
5
4
3
2
1
ppm
6.09
BRUKER
Current Data Parameters
NAME AKA-VII-96
EXPNO 1
PROCNO 1
F2 - Acquisition Parameters
Date_ 20201105
Time 17.56
INSTRUM spect
PROBHD 5 mm PABBO BB/
PULPROG zg30
TD 65536
SOLVENT DMSO
NS 16
DS 2
SWH 8012.820 Hz
FIDRES 0.122266 Hz
AQ 4.0894465 sec
RG 133.17
DW 62.400 usec
DE 6.50 usec
TE 298.5 K
D1 1.00000000 sec
TD0 1
======== CHANNEL f1 ========
SFO1 400.2124715 MHz
NUC1 1H
P1 15.54 usec
PLW1 12.00000000 W
F2 - Processing parameters
SI 65536
SF 400.2100000 MHz
WDW EM
SSB 0
LB 0.30 Hz
GB 0
PC 1.00

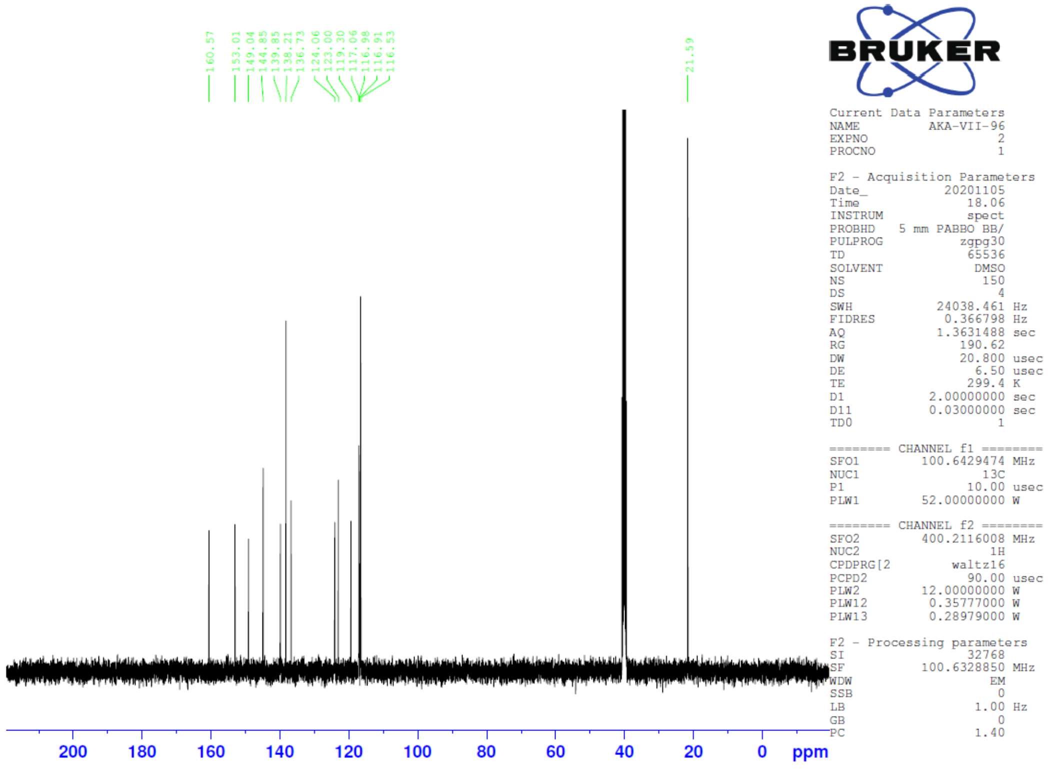
160.57
153.01
149.04
144.85
139.85
138.21
136.73
124.06
123.00
119.30
117.06
116.98
116.91
116.53
21.59
200
180
160
140
120
100
80
60
40
20
0
ppm
BRUKER
Current Data Parameters
NAME AKA-VII-96
EXPNO 2
PROCNO 1
F2 - Acquisition Parameters
Date_ 20201105
Time 18.06
INSTRUM spect
PROBHD 5 mm PABBO BB/
PULPROG zgpg30
TD 65536
SOLVENT DMSO
NS 150
DS 4
SWH 24038.461 Hz
FIDRES 0.366798 Hz
AQ 1.3631488 sec
RG 190.62
DW 20.800 usec
DE 6.50 usec
TE 299.4 K
D1 2.00000000 sec
D11 0.03000000 sec
TD0 1
======== CHANNEL f1 ========
SFO1 100.6429474 MHz
NUC1 13C
P1 10.00 usec
PLW1 52.00000000 W
======== CHANNEL f2 ========
SFO2 400.2116008 MHz
NUC2 1H
CPDPRG[2 waltz16
PCPD2 90.00 usec
PLW2 12.00000000 W
PLW12 0.35777000 W
PLW13 0.28979000 W
F2 - Processing parameters
SI 32768
SF 100.6328850 MHz
WDW EM
SSB 0
LB 1.00 Hz
GB 0
PC 1.40

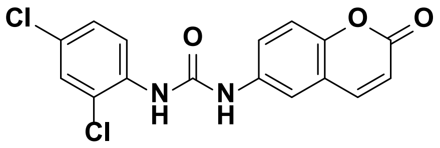
Cl
O
N
H
N
H
O
O
Cl

5l

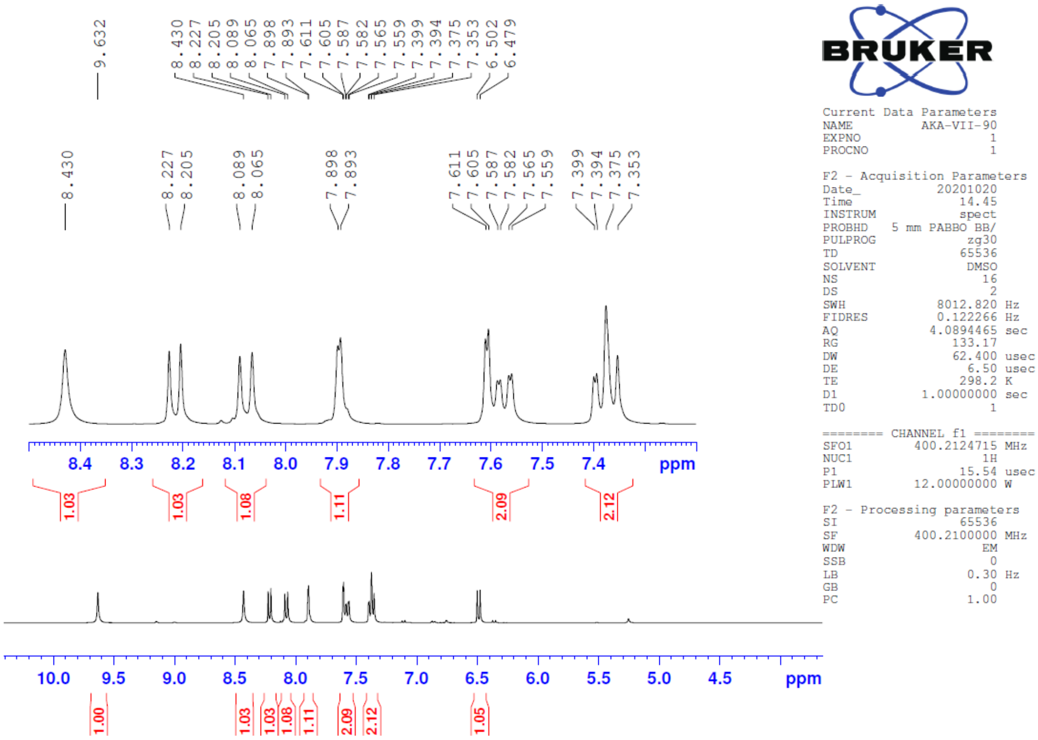
9.632
8.430
8.227
8.205
8.089
8.065
7.898
7.893
7.611
7.605
7.587
7.582
7.565
7.559
7.399
7.394
7.375
7.353
6.502
6.479
BRUKER
Current Data Parameters
NAME AKA-VII-90
EXPNO 1
PROCNO 1
F2 - Acquisition Parameters
Date_ 20201020
Time 14.45
INSTRUM spect
PROBHD 5 mm PABBO BB/
PULPROG zg30
TD 65536
SOLVENT DMSO
NS 16
DS 2
SWH 8012.820 Hz
FIDRES 0.122266 Hz
AQ 4.0894465 sec
RG 133.17
DW 62.400 usec
DE 6.50 usec
TE 298.2 K
D1 1.00000000 sec
TD0 1
======== CHANNEL f1 ========
SFO1 400.2124715 MHz
NUC1 1H
P1 15.54 usec
PLW1 12.00000000 W
F2 - Processing parameters
SI 65536
SF 400.2100000 MHz
WDW EM
SSB 0
LB 0.30 Hz
GB 0
PC 1.00
8.4
8.3
8.2
8.1
8.0
7.9
7.8
7.7
7.6
7.5
7.4
ppm
1.03
1.03
1.08
1.11
2.09
2.12
10.0
9.5
9.0
8.5
8.0
7.5
7.0
6.5
6.0
5.5
5.0
4.5
ppm
1.00
1.03
1.03
1.08
1.11
2.09
2.12
1.05

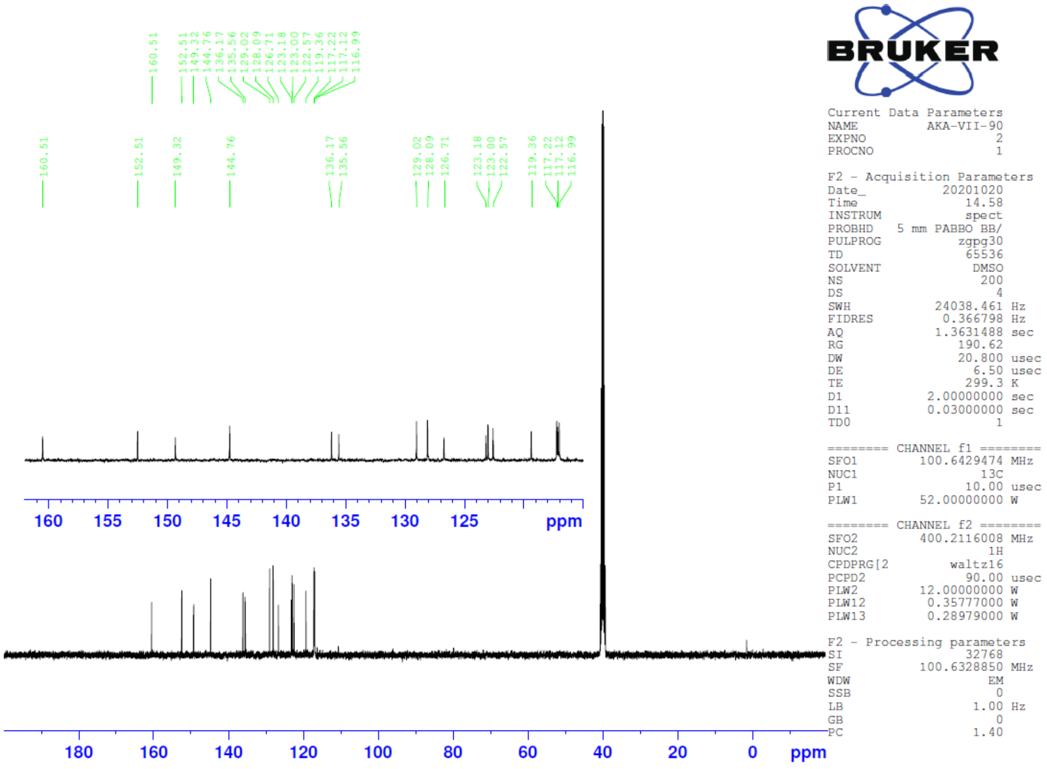
160.51
152.51
149.32
144.76
136.17
135.56
129.02
128.09
126.71
123.18
123.00
122.57
119.36
117.22
117.12
116.99
BRUKER
Current Data Parameters
NAME AKA-VII-90
EXPNO 2
PROCNO 1
F2 - Acquisition Parameters
Date_ 20201020
Time 14.58
INSTRUM spect
PROBHD 5 mm PABBO BB/
PULPROG zgpg30
TD 65536
SOLVENT DMSO
NS 200
DS 4
SWH 24038.461 Hz
FIDRES 0.366798 Hz
AQ 1.3631488 sec
RG 190.62
DW 20.800 usec
DE 6.50 usec
TE 299.3 K
D1 2.00000000 sec
D11 0.03000000 sec
TD0 1
======== CHANNEL f1 ========
SFO1 100.6429474 MHz
NUC1 13C
P1 10.00 usec
PLW1 52.00000000 W
======== CHANNEL f2 ========
SFO2 400.2116008 MHz
NUC2 1H
CPDPRG[2 waltz16
PCPD2 90.00 usec
PLW2 12.00000000 W
PLW12 0.35777000 W
PLW13 0.28979000 W
F2 - Processing parameters
SI 32768
SF 100.6328850 MHz
WDW EM
SSB 0
LB 1.00 Hz
GB 0
PC 1.40
160
155
150
145
140
135
130
125
ppm
180
160
140
120
100
80
60
40
20
0
ppm

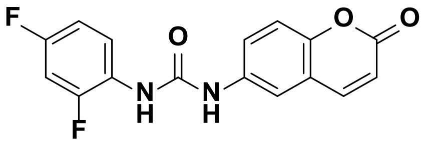
F
O
O
O
F
N
H
N
H

5m

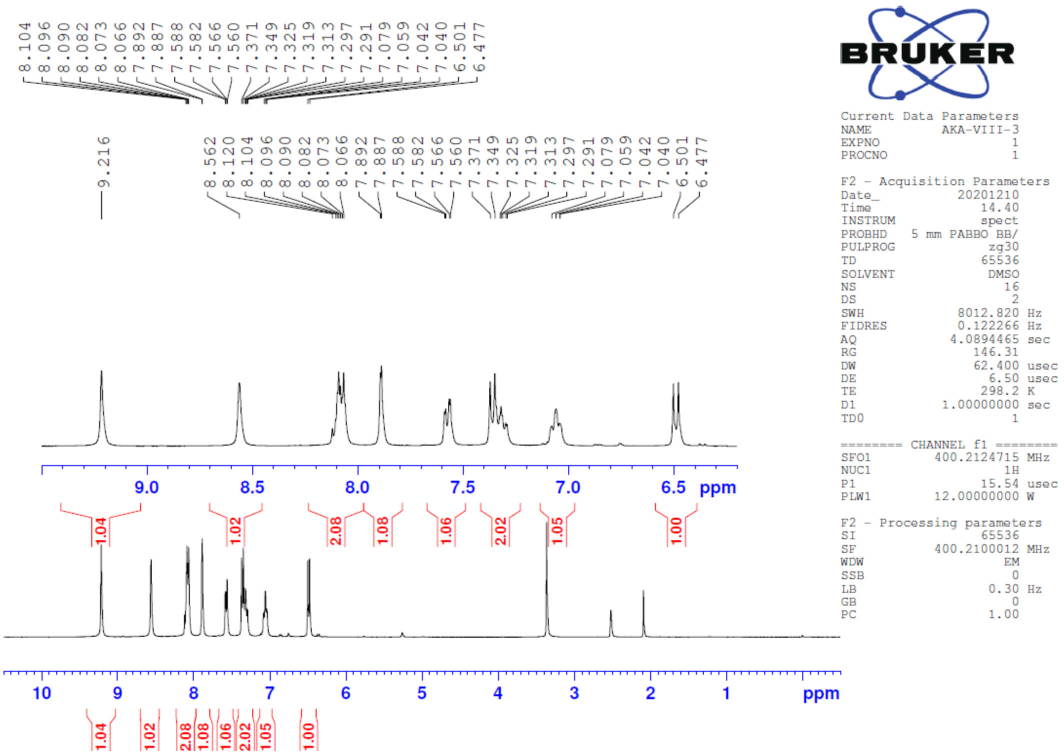
8.104
8.096
8.090
8.082
8.073
8.066
7.892
7.887
7.588
7.582
7.566
7.560
7.371
7.349
7.325
7.319
7.313
7.297
7.291
7.079
7.059
7.042
7.040
6.501
6.477
9.216
8.562
8.120
8.104
8.096
8.090
8.082
8.073
8.066
7.892
7.887
7.588
7.582
7.566
7.560
7.371
7.349
7.325
7.319
7.313
7.297
7.291
7.079
7.059
7.042
7.040
6.501
6.477
9.0
8.5
8.0
7.5
7.0
6.5
ppm
1.04
1.02
2.08
1.08
1.06
2.02
1.05
1.00
10
9
8
7
6
5
4
3
2
1
ppm
BRUKER
Current Data Parameters
NAME AKA-VIII-3
EXPNO 1
PROCNO 1
F2 - Acquisition Parameters
Date_ 20201210
Time 14.40
INSTRUM spect
PROBHD 5 mm PABBO BB/
PULPROG zg30
TD 65536
SOLVENT DMSO
NS 16
DS 2
SWH 8012.820 Hz
FIDRES 0.122266 Hz
AQ 4.0894465 sec
RG 146.31
DW 62.400 usec
DE 6.50 usec
TE 298.2 K
D1 1.00000000 sec
TD0 1
======== CHANNEL f1 ========
SFO1 400.2124715 MHz
NUC1 1H
P1 15.54 usec
PLW1 12.00000000 W
F2 - Processing parameters
SI 65536
SF 400.2100012 MHz
WDW EM
SSB 0
LB 0.30 Hz
GB 0
PC 1.00

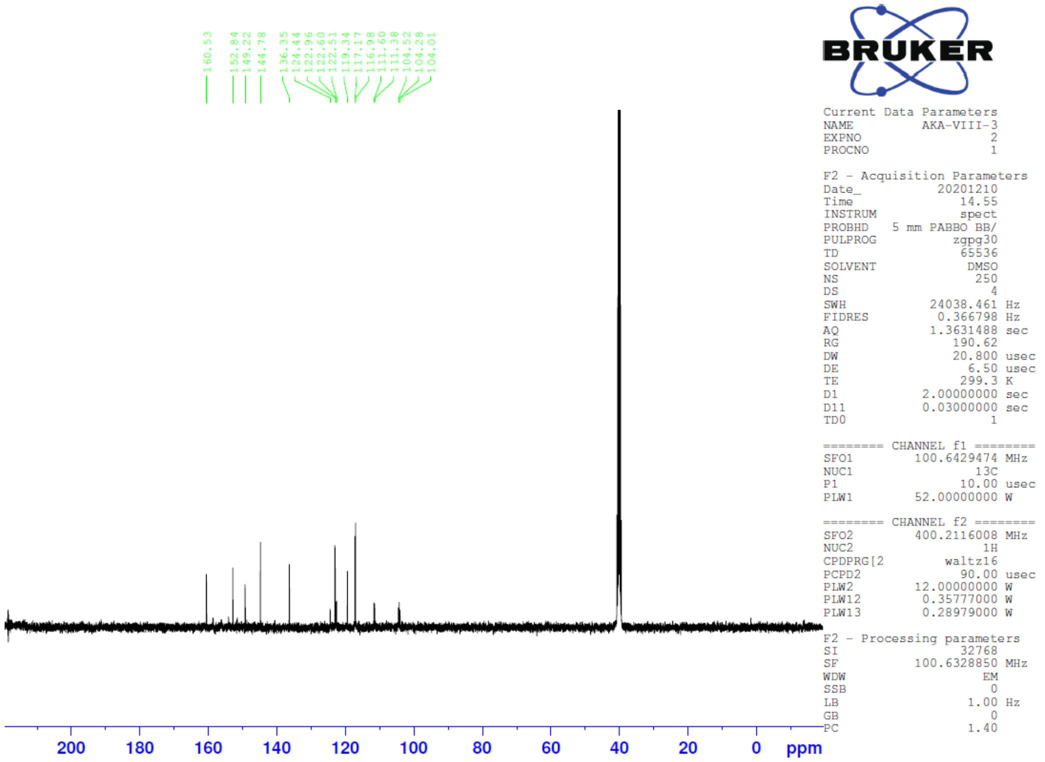
160.53
152.84
149.22
144.78
136.35
124.44
122.96
122.60
122.51
119.34
117.17
116.98
111.60
111.38
104.52
104.28
104.01
200
180
160
140
120
100
80
60
40
20
0
ppm
BRUKER
Current Data Parameters
NAME AKA-VIII-3
EXPNO 2
PROCNO 1
F2 - Acquisition Parameters
Date_ 20201210
Time 14.55
INSTRUM spect
PROBHD 5 mm PABBO BB/
PULPROG zgpg30
TD 65536
SOLVENT DMSO
NS 250
DS 4
SWH 24038.461 Hz
FIDRES 0.366798 Hz
AQ 1.3631488 sec
RG 190.62
DW 20.800 usec
DE 6.50 usec
TE 299.3 K
D1 2.00000000 sec
D11 0.03000000 sec
TD0 1
======== CHANNEL f1 ========
SFO1 100.6429474 MHz
NUC1 13C
P1 10.00 usec
PLW1 52.00000000 W
======== CHANNEL f2 ========
SFO2 400.2116008 MHz
NUC2 1H
CPDPRG[2 waltz16
PCPD2 90.00 usec
PLW2 12.00000000 W
PLW12 0.35777000 W
PLW13 0.28979000 W
F2 - Processing parameters
SI 32768
SF 100.6328850 MHz
WDW EM
SSB 0
LB 1.00 Hz
GB 0
PC 1.40

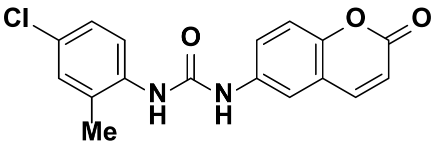

Cl
O
N
H
N
H
O
O
Me

5n

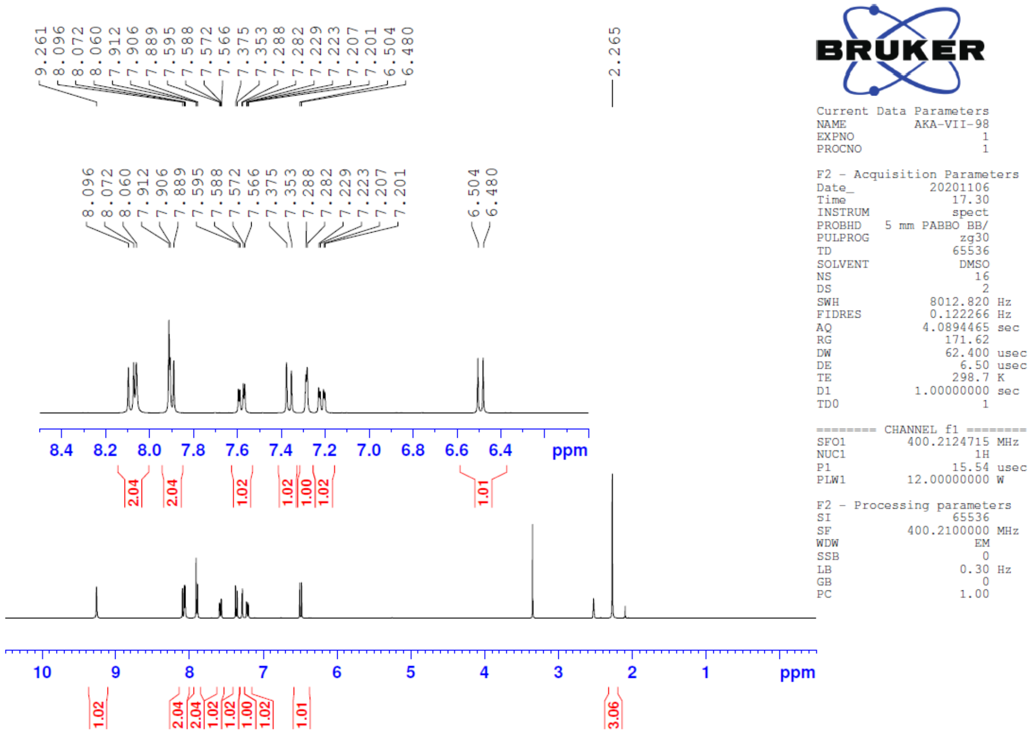

9.261 8.096 8.072 8.060 7.912 7.906 7.889 7.595 7.588 7.572 7.566 7.375 7.353 7.288 7.282 7.229 7.223 7.207 7.201 6.504 6.480
2.265
8.096 8.072 8.060 7.912 7.906 7.889 7.595 7.588 7.572 7.566 7.375 7.353 7.288 7.282 7.229 7.223 7.207 7.201
6.504 6.480
8.4 8.2 8.0 7.8 7.6 7.4 7.2 7.0 6.8 6.6 6.4 ppm
2.04 2.04 1.02 1.02 1.00 1.02 1.01
10 9 8 7 6 5 4 3 2 1 ppm
1.02 2.04 2.04 1.02 1.02 1.00 1.02 1.01 3.06
BRUKER
Current Data Parameters
NAME AKA-VII-98
EXPNO 1
PROCNO 1
F2 - Acquisition Parameters
Date_ 20201106
Time 17.30
INSTRUM spect
PROBHD 5 mm PABBO BB/
PULPROG zg30
TD 65536
SOLVENT DMSO
NS 16
DS 2
SWH 8012.820 Hz
FIDRES 0.122266 Hz
AQ 4.0894465 sec
RG 171.62
DW 62.400 usec
DE 6.50 usec
TE 298.7 K
D1 1.00000000 sec
TD0 1
======== CHANNEL f1 ========
SFO1 400.2124715 MHz
NUC1 1H
P1 15.54 usec
PLW1 12.00000000 W
F2 - Processing parameters
SI 65536
SF 400.2100000 MHz
WDW EM
SSB 0
LB 0.30 Hz
GB 0
PC 1.00

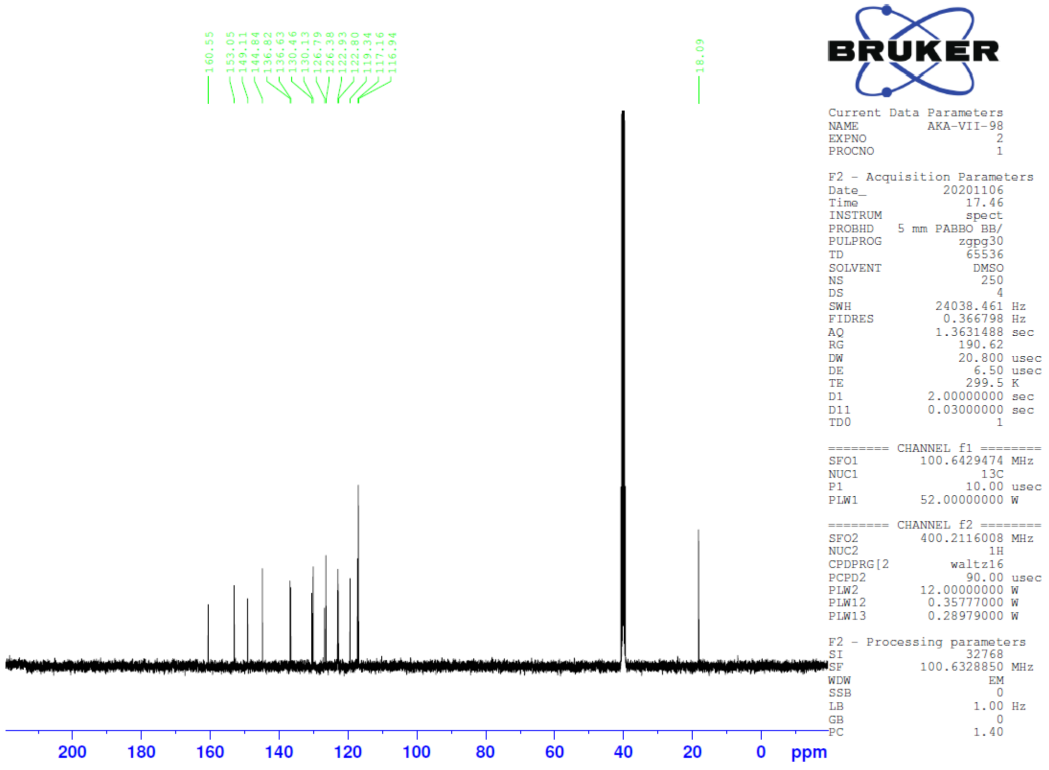

160.55 153.05 149.11 144.84 136.82 136.63 130.46 130.13 126.79 126.38 122.93 122.80 119.34 117.16 116.94
18.09
200 180 160 140 120 100 80 60 40 20 0 ppm
BRUKER
Current Data Parameters
NAME AKA-VII-98
EXPNO 2
PROCNO 1
F2 - Acquisition Parameters
Date_ 20201106
Time 17.46
INSTRUM spect
PROBHD 5 mm PABBO BB/
PULPROG zgpg30
TD 65536
SOLVENT DMSO
NS 250
DS 4
SWH 24038.461 Hz
FIDRES 0.366798 Hz
AQ 1.3631488 sec
RG 190.62
DW 20.800 usec
DE 6.50 usec
TE 299.5 K
D1 2.00000000 sec
D11 0.03000000 sec
TD0 1
======== CHANNEL f1 ========
SFO1 100.6429474 MHz
NUC1 13C
P1 10.00 usec
PLW1 52.00000000 W
======== CHANNEL f2 ========
SFO2 400.2116008 MHz
NUC2 1H
CPDPRG[2 waltz16
PCPD2 90.00 usec
PLW2 12.00000000 W
PLW12 0.35777000 W
PLW13 0.28979000 W
F2 - Processing parameters
SI 32768
SF 100.6328850 MHz
WDW EM
SSB 0
LB 1.00 Hz
GB 0
PC 1.40

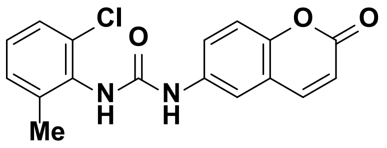

Cl
O
O
O
N
H
N
H
Me

5o

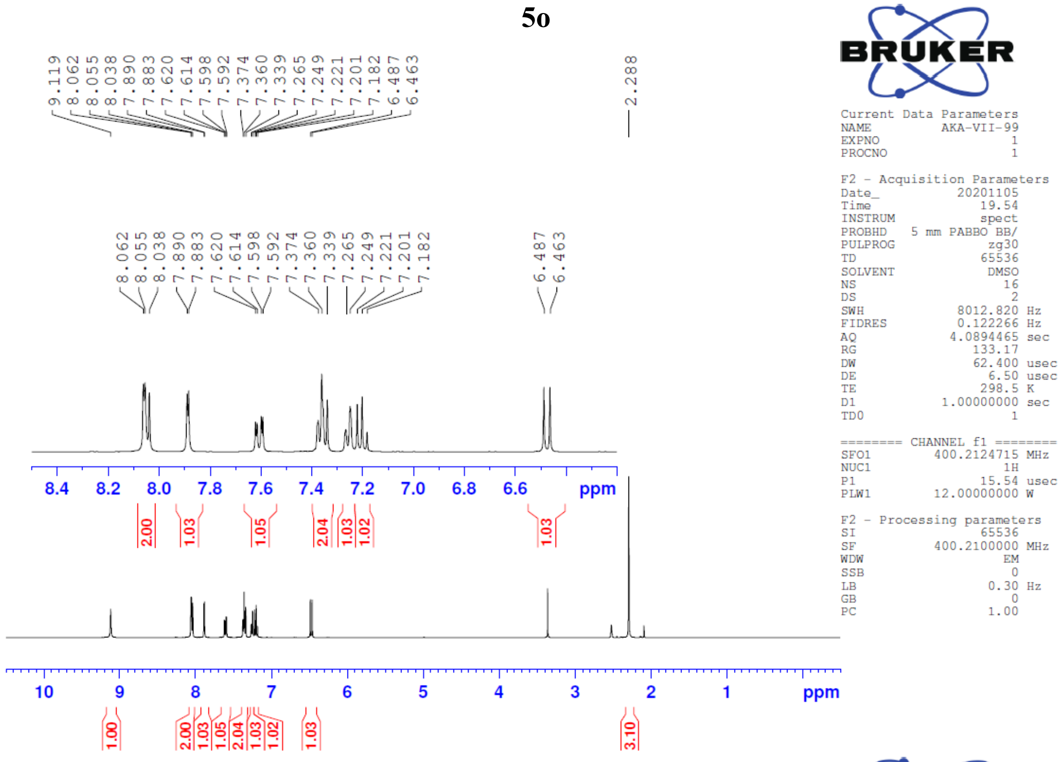

9.119
8.062
8.055
8.038
7.890
7.883
7.620
7.614
7.598
7.592
7.374
7.360
7.339
7.265
7.249
7.221
7.201
7.182
6.487
6.463
2.288
2.00
1.03
1.05
2.04
1.03
1.02
1.03
1.00
3.10
ppm
BRUKER
Current Data Parameters
NAME AKA-VII-99
EXPNO 1
PROCNO 1
F2 - Acquisition Parameters
Date_ 20201105
Time 19.54
INSTRUM spect
PROBHD 5 mm PABBO BB/
PULPROG zg30
TD 65536
SOLVENT DMSO
NS 16
DS 2
SWH 8012.820 Hz
FIDRES 0.122266 Hz
AQ 4.0894465 sec
RG 133.17
DW 62.400 usec
DE 6.50 usec
TE 298.5 K
D1 1.00000000 sec
TD0 1
======== CHANNEL f1 ========
SFO1 400.2124715 MHz
NUC1 1H
P1 15.54 usec
PLW1 12.00000000 W
F2 - Processing parameters
SI 65536
SF 400.2100000 MHz
WDW EM
SSB 0
LB 0.30 Hz
GB 0
PC 1.00

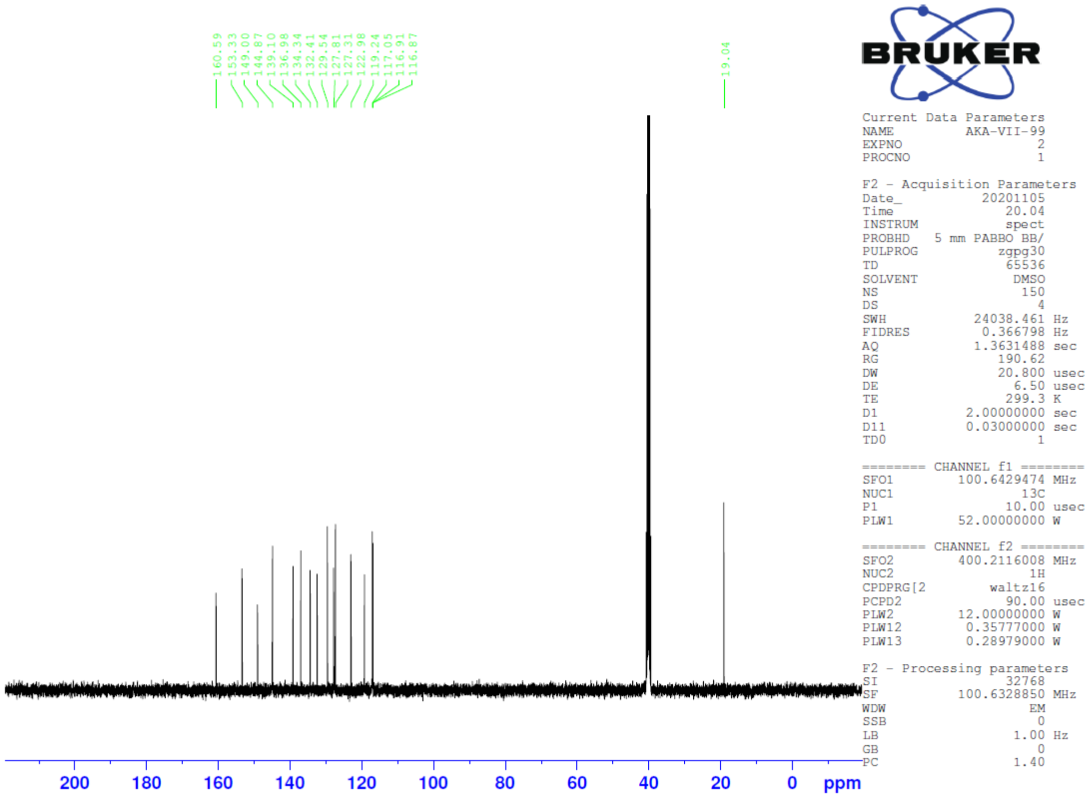

160.59
153.33
149.00
144.87
139.10
136.98
134.34
132.41
129.54
127.81
127.31
122.98
119.24
117.05
116.91
116.87
19.04
ppm
BRUKER
Current Data Parameters
NAME AKA-VII-99
EXPNO 2
PROCNO 1
F2 - Acquisition Parameters
Date_ 20201105
Time 20.04
INSTRUM spect
PROBHD 5 mm PABBO BB/
PULPROG zgpg30
TD 65536
SOLVENT DMSO
NS 150
DS 4
SWH 24038.461 Hz
FIDRES 0.366798 Hz
AQ 1.3631488 sec
RG 190.62
DW 20.800 usec
DE 6.50 usec
TE 299.3 K
D1 2.00000000 sec
D11 0.03000000 sec
TD0 1
======== CHANNEL f1 ========
SFO1 100.6429474 MHz
NUC1 13C
P1 10.00 usec
PLW1 52.00000000 W
======== CHANNEL f2 ========
SFO2 400.2116008 MHz
NUC2 1H
CPDPRG[2 waltz16
PCPD2 90.00 usec
PLW2 12.00000000 W
PLW12 0.35777000 W
PLW13 0.28979000 W
F2 - Processing parameters
SI 32768
SF 100.6328850 MHz
WDW EM
SSB 0
LB 1.00 Hz
GB 0
PC 1.40

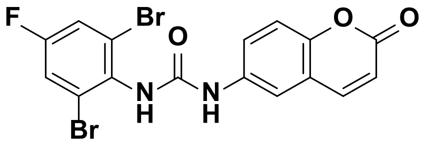
F
Br
O
N
H
N
H
Br
O
O

5p

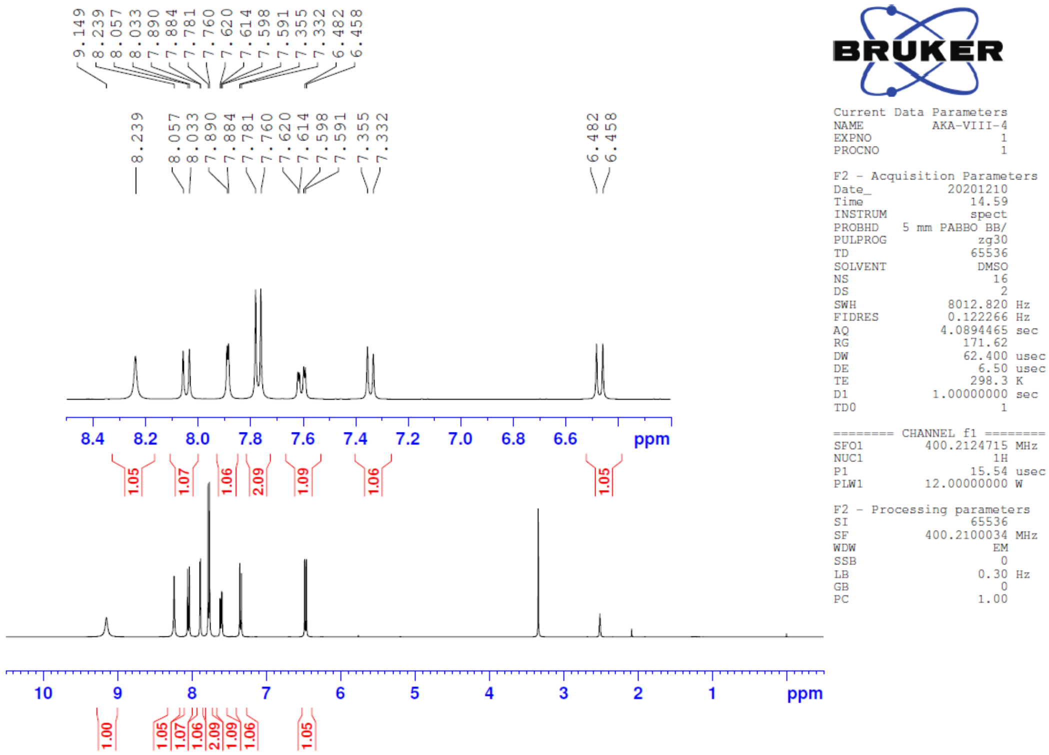
9.149
8.239
8.057
8.033
7.890
7.884
7.781
7.760
7.620
7.614
7.598
7.591
7.355
7.332
6.482
6.458
1.05
1.07
1.06
2.09
1.09
1.06
1.05
1.00
ppm
BRUKER
Current Data Parameters
NAME AKA-VIII-4
EXPNO 1
PROCNO 1
F2 - Acquisition Parameters
Date_ 20201210
Time 14.59
INSTRUM spect
PROBHD 5 mm PABBO BB/
PULPROG zg30
TD 65536
SOLVENT DMSO
NS 16
DS 2
SWH 8012.820 Hz
FIDRES 0.122266 Hz
AQ 4.0894465 sec
RG 171.62
DW 62.400 usec
DE 6.50 usec
TE 298.3 K
D1 1.00000000 sec
TD0 1
======== CHANNEL f1 ========
SFO1 400.2124715 MHz
NUC1 1H
P1 15.54 usec
PLW1 12.00000000 W
F2 - Processing parameters
SI 65536
SF 400.2100034 MHz
WDW EM
SSB 0
LB 0.30 Hz
GB 0
PC 1.00

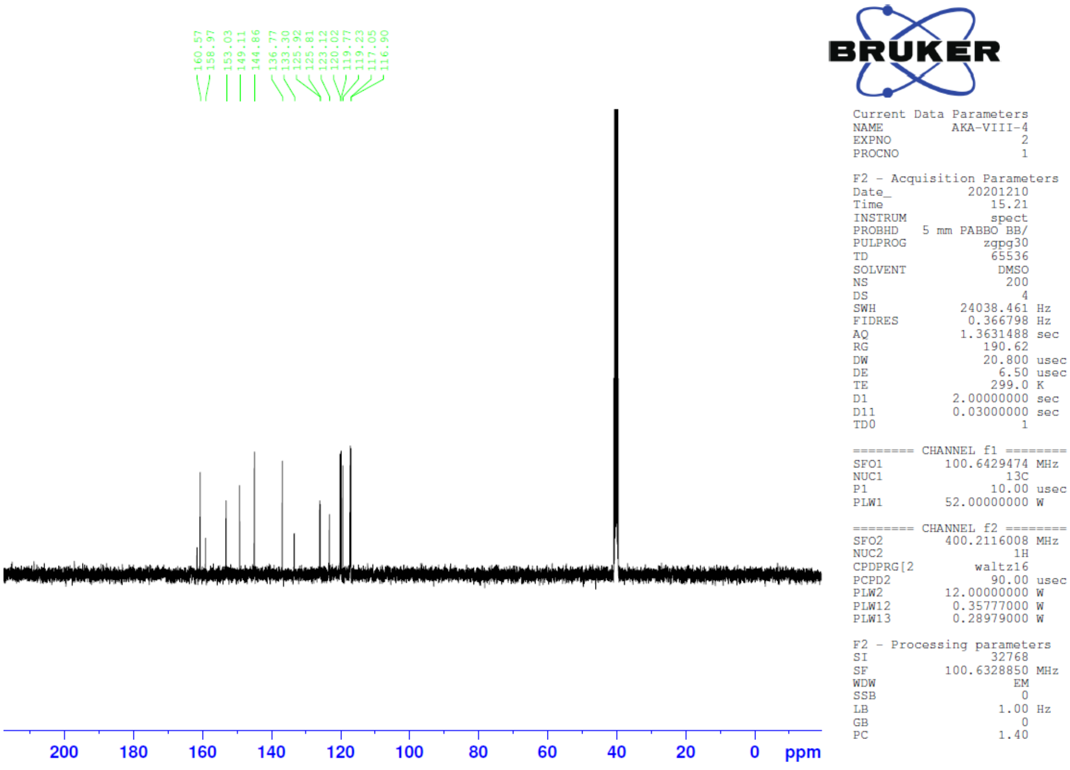
160.57
158.97
153.03
149.11
144.86
136.77
133.30
125.92
125.81
123.12
120.02
119.77
119.23
117.05
116.90
ppm
BRUKER
Current Data Parameters
NAME AKA-VIII-4
EXPNO 2
PROCNO 1
F2 - Acquisition Parameters
Date_ 20201210
Time 15.21
INSTRUM spect
PROBHD 5 mm PABBO BB/
PULPROG zgpg30
TD 65536
SOLVENT DMSO
NS 200
DS 4
SWH 24038.461 Hz
FIDRES 0.366798 Hz
AQ 1.3631488 sec
RG 190.62
DW 20.800 usec
DE 6.50 usec
TE 299.0 K
D1 2.00000000 sec
D11 0.03000000 sec
TD0 1
======== CHANNEL f1 ========
SFO1 100.6429474 MHz
NUC1 13C
P1 10.00 usec
PLW1 52.00000000 W
======== CHANNEL f2 ========
SFO2 400.2116008 MHz
NUC2 1H
CPDPRG[2 waltz16
PCPD2 90.00 usec
PLW2 12.00000000 W
PLW12 0.35777000 W
PLW13 0.28979000 W
F2 - Processing parameters
SI 32768
SF 100.6328850 MHz
WDW EM
SSB 0
LB 1.00 Hz
GB 0
PC 1.40

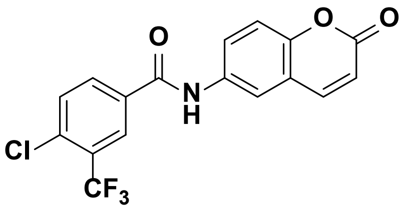
O
O
O
N
H
Cl
CF3

7a

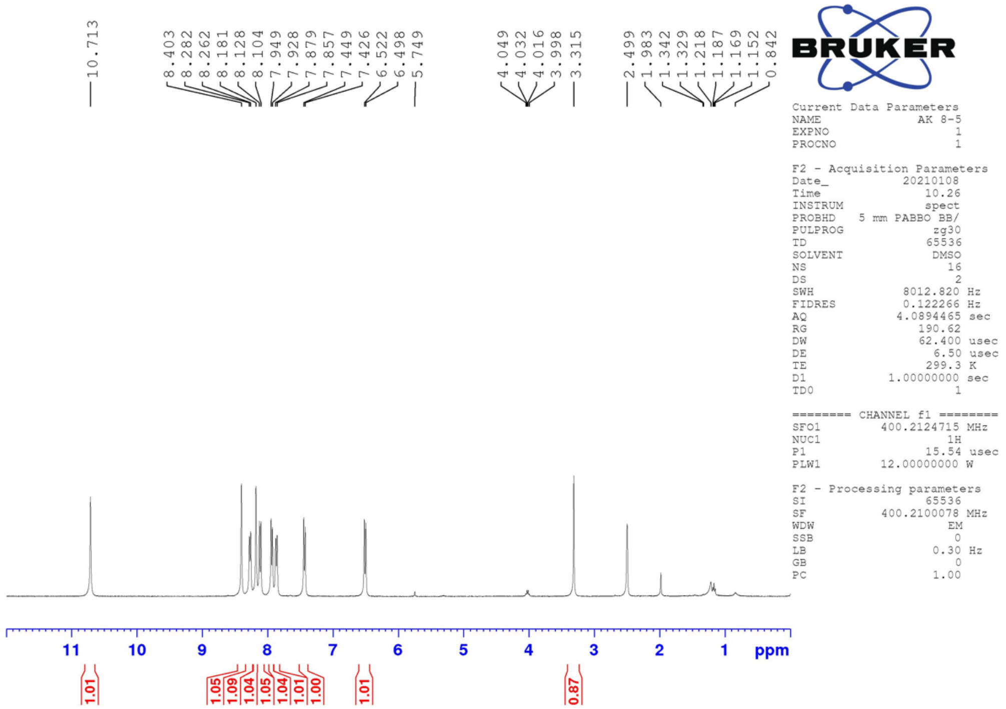
10.713
8.403
8.282
8.262
8.181
8.128
8.104
7.949
7.928
7.879
7.857
7.449
7.426
6.522
6.498
5.749
4.049
4.032
4.016
3.998
3.315
2.499
1.983
1.342
1.329
1.218
1.187
1.169
1.152
0.842
BRUKER
Current Data Parameters
NAME AK 8-5
EXPNO 1
PROCNO 1
F2 - Acquisition Parameters
Date_ 20210108
Time 10.26
INSTRUM spect
PROBHD 5 mm PABBO BB/
PULPROG zg30
TD 65536
SOLVENT DMSO
NS 16
DS 2
SWH 8012.820 Hz
FIDRES 0.122266 Hz
AQ 4.0894465 sec
RG 190.62
DW 62.400 usec
DE 6.50 usec
TE 299.3 K
D1 1.00000000 sec
TD0 1
======== CHANNEL f1 ========
SFO1 400.2124715 MHz
NUC1 1H
P1 15.54 usec
PLW1 12.00000000 W
F2 - Processing parameters
SI 65536
SF 400.2100078 MHz
WDW EM
SSB 0
LB 0.30 Hz
GB 0
PC 1.00
11 10 9 8 7 6 5 4 3 2 1 ppm
1.01
1.05
1.09
1.04
1.05
1.04
1.01
1.00
1.01
0.87

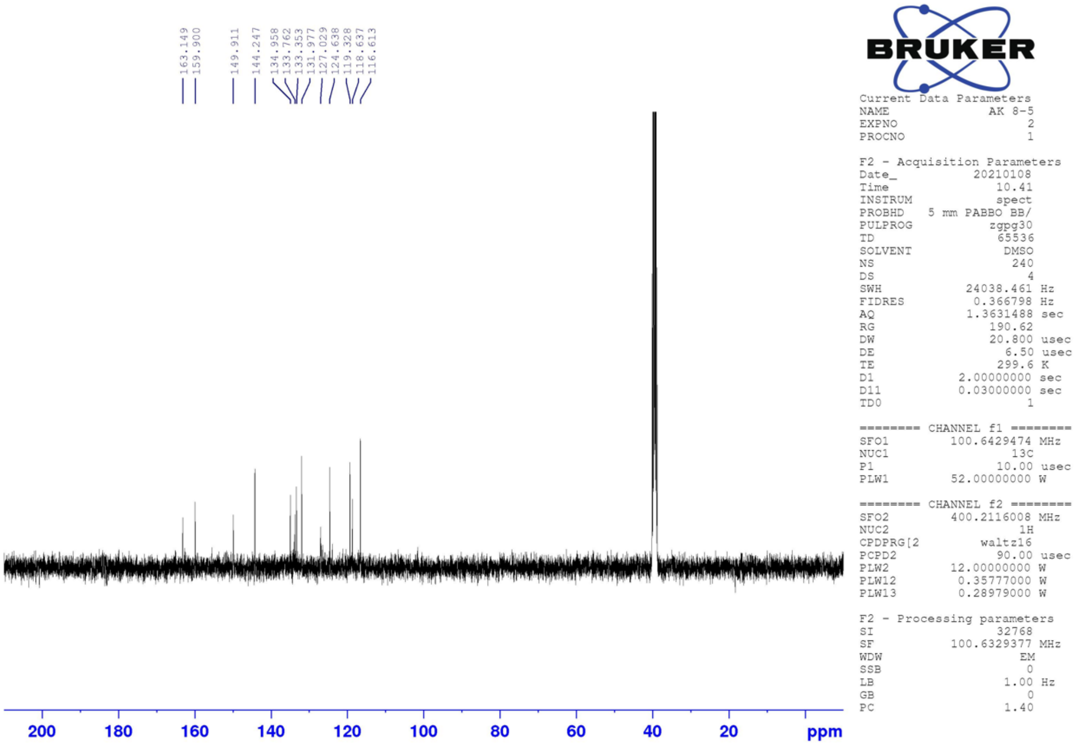
163.149
159.900
149.911
144.247
134.958
133.762
133.353
131.977
127.029
124.638
119.328
118.637
116.613
BRUKER
Current Data Parameters
NAME AK 8-5
EXPNO 2
PROCNO 1
F2 - Acquisition Parameters
Date_ 20210108
Time 10.41
INSTRUM spect
PROBHD 5 mm PABBO BB/
PULPROG zgpg30
TD 65536
SOLVENT DMSO
NS 240
DS 4
SWH 24038.461 Hz
FIDRES 0.366798 Hz
AQ 1.3631488 sec
RG 190.62
DW 20.800 usec
DE 6.50 usec
TE 299.6 K
D1 2.00000000 sec
D11 0.03000000 sec
TD0 1
======== CHANNEL f1 ========
SFO1 100.6429474 MHz
NUC1 13C
P1 10.00 usec
PLW1 52.00000000 W
======== CHANNEL f2 ========
SFO2 400.2116008 MHz
NUC2 1H
CPDPRG[2 waltz16
PCPD2 90.00 usec
PLW2 12.00000000 W
PLW12 0.35777000 W
PLW13 0.28979000 W
F2 - Processing parameters
SI 32768
SF 100.6329377 MHz
WDW EM
SSB 0
LB 1.00 Hz
GB 0
PC 1.40
200 180 160 140 120 100 80 60 40 20 ppm

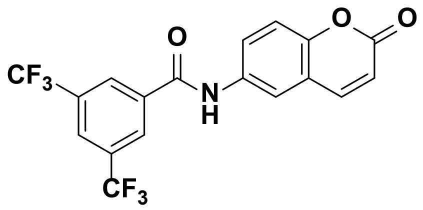

O
O
O
CF3
N
H
CF3

7b

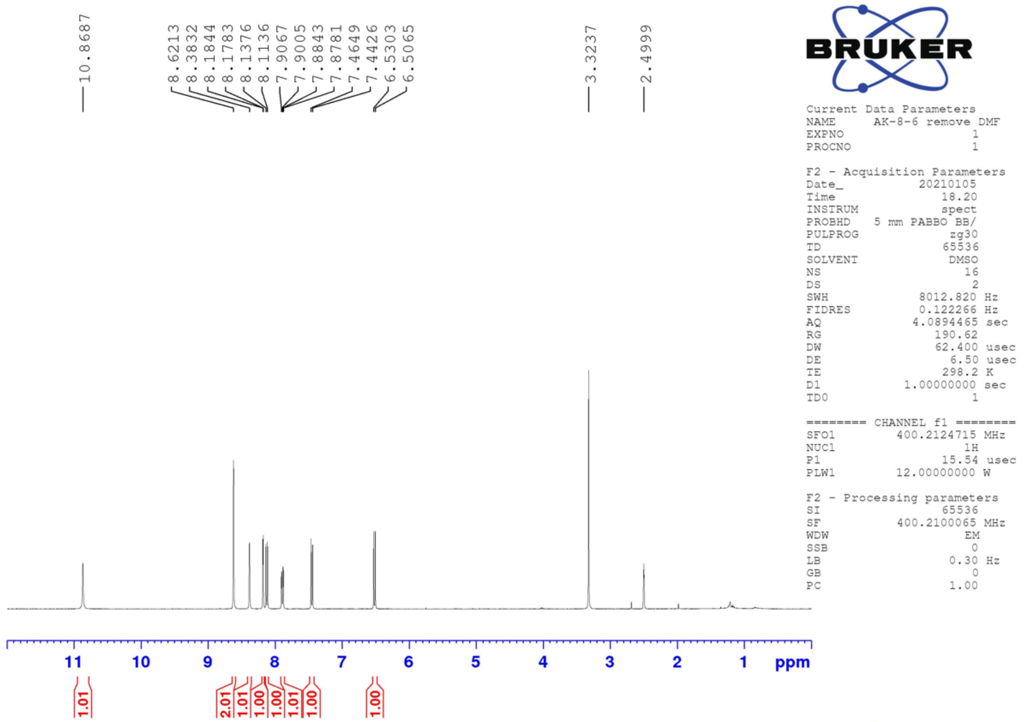

10.8687
8.6213
8.3832
8.1844
8.1783
8.1376
8.1136
7.9067
7.9005
7.8843
7.8781
7.4649
7.4426
6.5303
6.5065
3.3237
2.4999
BRUKER
Current Data Parameters
NAME AK-8-6 remove DMF
EXPNO 1
PROCNO 1
F2 - Acquisition Parameters
Date_ 20210105
Time 18.20
INSTRUM spect
PROBHD 5 mm PABBO BB/
PULPROG zg30
TD 65536
SOLVENT DMSO
NS 16
DS 2
SWH 8012.820 Hz
FIDRES 0.122266 Hz
AQ 4.0894465 sec
RG 190.62
DW 62.400 usec
DE 6.50 usec
TE 298.2 K
D1 1.00000000 sec
TD0 1
======== CHANNEL f1 ========
SFO1 400.2124715 MHz
NUC1 1H
P1 15.54 usec
PLW1 12.00000000 W
F2 - Processing parameters
SI 65536
SF 400.2100065 MHz
WDW EM
SSB 0
LB 0.30 Hz
GB 0
PC 1.00
11
10
9
8
7
6
5
4
3
2
1
ppm
1.01
2.01
1.01
1.00
1.00
1.01
1.00
1.00

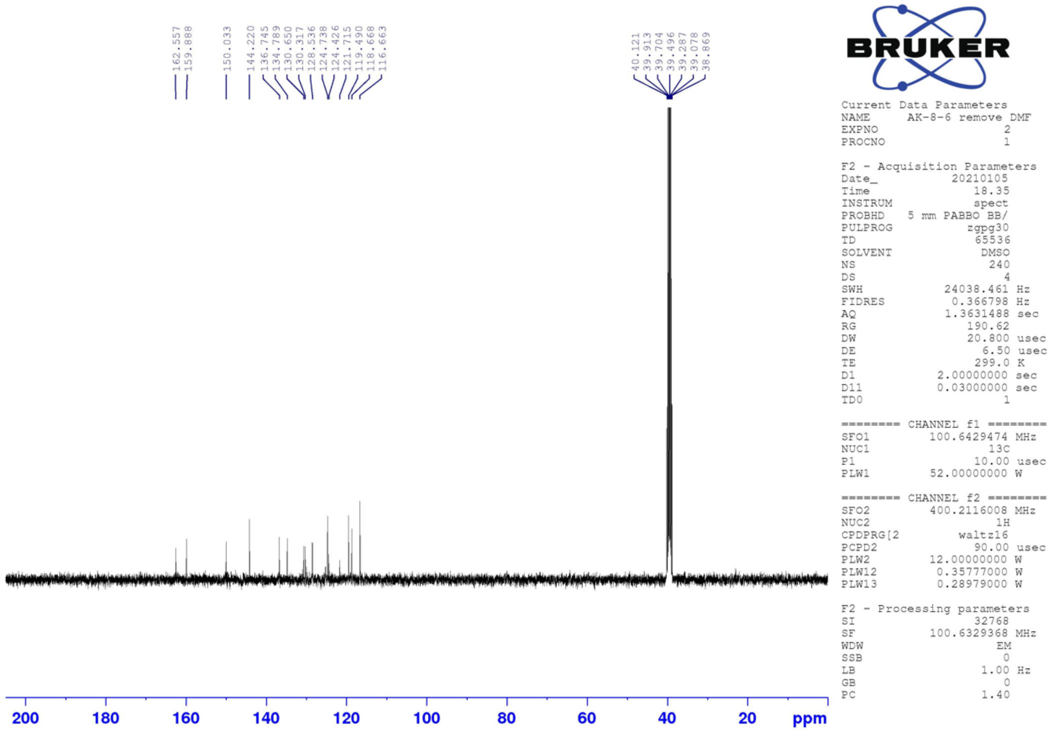

162.557
159.888
150.033
144.220
136.745
134.789
130.650
130.317
128.536
124.738
124.426
121.715
119.490
118.668
116.663
40.121
39.913
39.704
39.496
39.287
39.078
38.869
BRUKER
Current Data Parameters
NAME AK-8-6 remove DMF
EXPNO 2
PROCNO 1
F2 - Acquisition Parameters
Date_ 20210105
Time 18.35
INSTRUM spect
PROBHD 5 mm PABBO BB/
PULPROG zgpg30
TD 65536
SOLVENT DMSO
NS 240
DS 4
SWH 24038.461 Hz
FIDRES 0.366798 Hz
AQ 1.3631488 sec
RG 190.62
DW 20.800 usec
DE 6.50 usec
TE 299.0 K
D1 2.00000000 sec
D11 0.03000000 sec
TD0 1
======== CHANNEL f1 ========
SFO1 100.6429474 MHz
NUC1 13C
P1 10.00 usec
PLW1 52.00000000 W
======== CHANNEL f2 ========
SFO2 400.2116008 MHz
NUC2 1H
CPDPRG[2 waltz16
PCPD2 90.00 usec
PLW2 12.00000000 W
PLW12 0.35777000 W
PLW13 0.28979000 W
F2 - Processing parameters
SI 32768
SF 100.6329368 MHz
WDW EM
SSB 0
LB 1.00 Hz
GB 0
PC 1.40
200
180
160
140
120
100
80
60
40
20
ppm

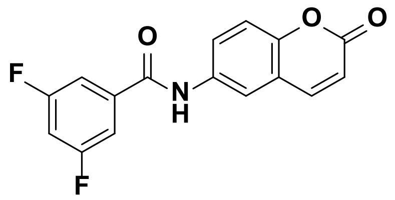

O
F
N
H
O
O
F

7c

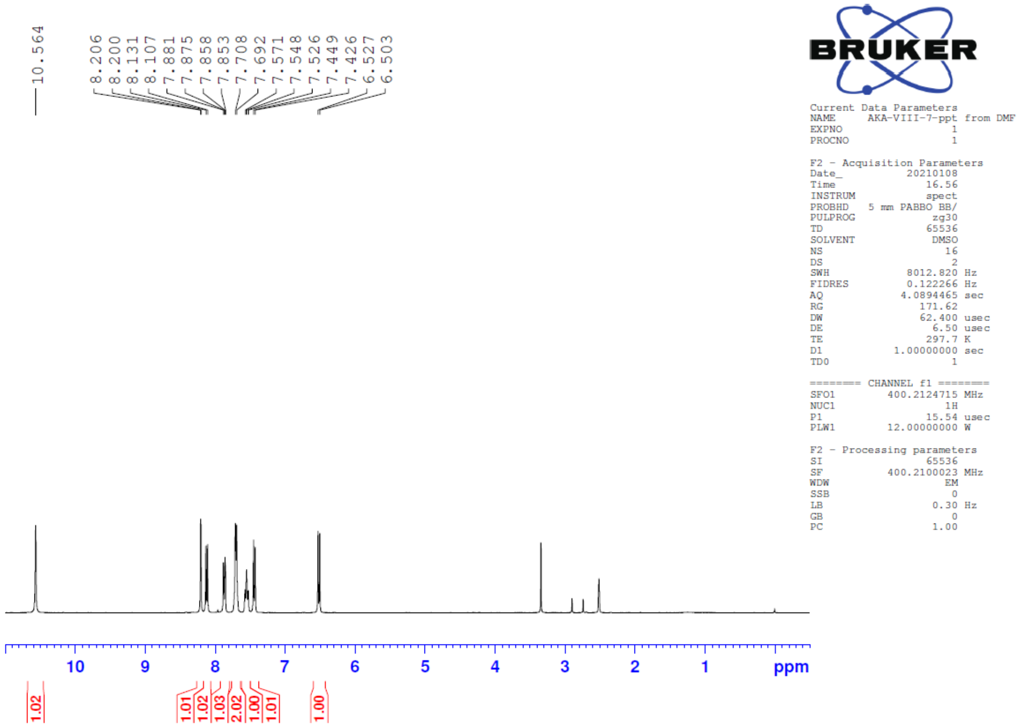

10.564
8.206
8.200
8.131
8.107
7.881
7.875
7.858
7.853
7.708
7.692
7.571
7.548
7.526
7.449
7.426
6.527
6.503
BRUKER
Current Data Parameters
NAME AKA-VIII-7-ppt from DMF
EXPNO 1
PROCNO 1
F2 - Acquisition Parameters
Date_ 20210108
Time 16.56
INSTRUM spect
PROBHD 5 mm PABBO BB/
PULPROG zg30
TD 65536
SOLVENT DMSO
NS 16
DS 2
SWH 8012.820 Hz
FIDRES 0.122266 Hz
AQ 4.0894465 sec
RG 171.62
DW 62.400 usec
DE 6.50 usec
TE 297.7 K
D1 1.00000000 sec
TD0 1
======= CHANNEL f1 =======
SFO1 400.2124715 MHz
NUC1 1H
P1 15.54 usec
PLW1 12.00000000 W
F2 - Processing parameters
SI 65536
SF 400.2100023 MHz
WDW EM
SSB 0
LB 0.30 Hz
GB 0
PC 1.00
10
9
8
7
6
5
4
3
2
1
ppm
1.02
1.01
1.02
1.03
2.02
1.00
1.01
1.00

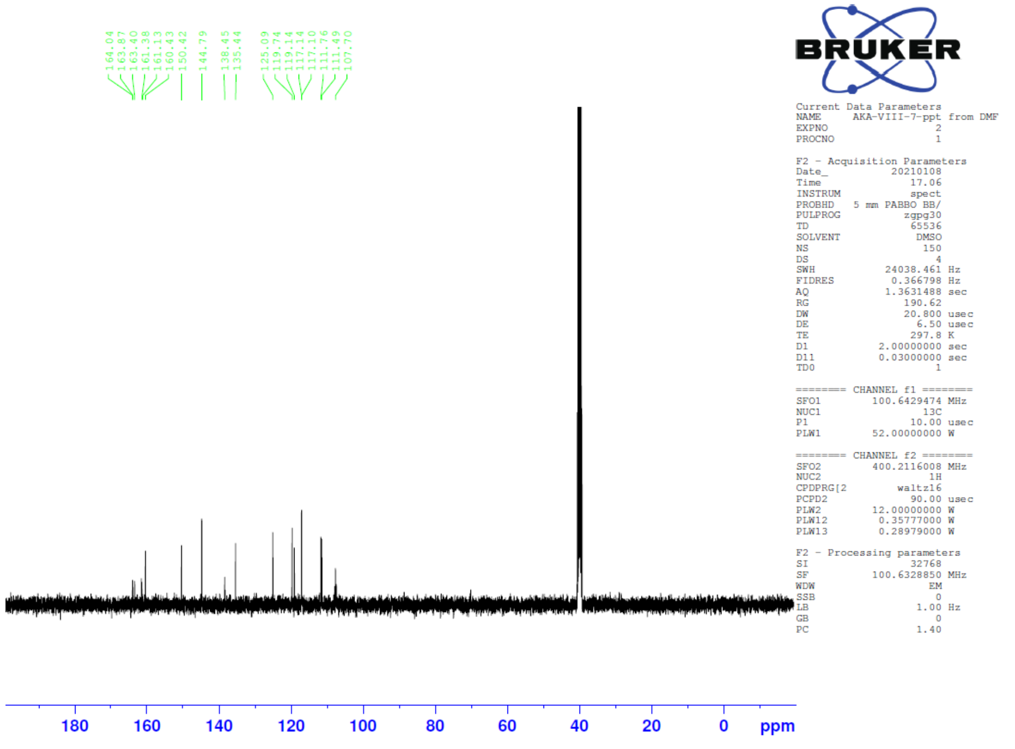

164.04
163.87
163.40
161.38
161.13
160.43
150.42
144.79
138.45
135.44
125.09
119.74
119.14
117.10
111.76
111.49
107.70
BRUKER
Current Data Parameters
NAME AKA-VIII-7-ppt from DMF
EXPNO 2
PROCNO 1
F2 - Acquisition Parameters
Date_ 20210108
Time 17.06
INSTRUM spect
PROBHD 5 mm PABBO BB/
PULPROG zgpg30
TD 65536
SOLVENT DMSO
NS 150
DS 4
SWH 24038.461 Hz
FIDRES 0.366798 Hz
AQ 1.3631488 sec
RG 190.62
DW 20.800 usec
DE 6.50 usec
TE 297.8 K
D1 2.00000000 sec
D11 0.03000000 sec
TD0 1
======= CHANNEL f1 =======
SFO1 100.6429474 MHz
NUC1 13C
P1 10.00 usec
PLW1 52.00000000 W
======= CHANNEL f2 =======
SFO2 400.2116008 MHz
NUC2 1H
CPDPRG[2 waltz16
PCPD2 90.00 usec
PLW2 12.00000000 W
PLW12 0.35777000 W
PLW13 0.28979000 W
F2 - Processing parameters
SI 32768
SF 100.6328850 MHz
WDW EM
SSB 0
LB 1.00 Hz
GB 0
PC 1.40
180
160
140
120
100
80
60
40
20
0
ppm

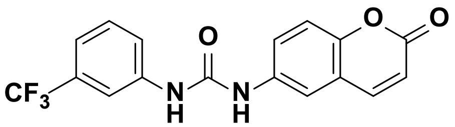

5a

[Mass Spectrum]
Data : KK9304 Date : 15-Mar-2021 16:24
Sample: -
Note : -
Inlet : Direct Ion Mode : EI+
Spectrum Type : Normal Ion [EF-Linear]
RT : 0.95 min Scan# : 20
BP : m/z 348.0719 Int. : 94.11
Output m/z range : 318.0000 to 378.0000 Cut Level : 0.00 %

[Elemental Composition]
Data : KK9304 Date : 15-Mar-2021 16:24
Sample: -
Note : -
Inlet : Direct Ion Mode : EI+
RT : 0.95 min Scan#: 20
Elements : C 17/0, H 11/0, F 3/0, N 2/0, O 3/0
Mass Tolerance : 1000ppm, 3mmu if m/z > 3
Unsaturation (U.S.) : -0.5 - 100.0

Observed m/z	Int%	Err[ppm / mmu]	U.S.	Composition
329.0737	13.4	-0.2 / -0.1	12.5	C 17 H 11 F 2 N 2 O 3
330.0620	60.9	+1.2 / +0.4	13.0	C 17 H 9 F 3 N 2 O 2
331.0658	12.6			
348.0719	100.0	-0.8 / -0.3	12.0	C 17 H 11 F 3 N 2 O 3
349.0750	21.5			

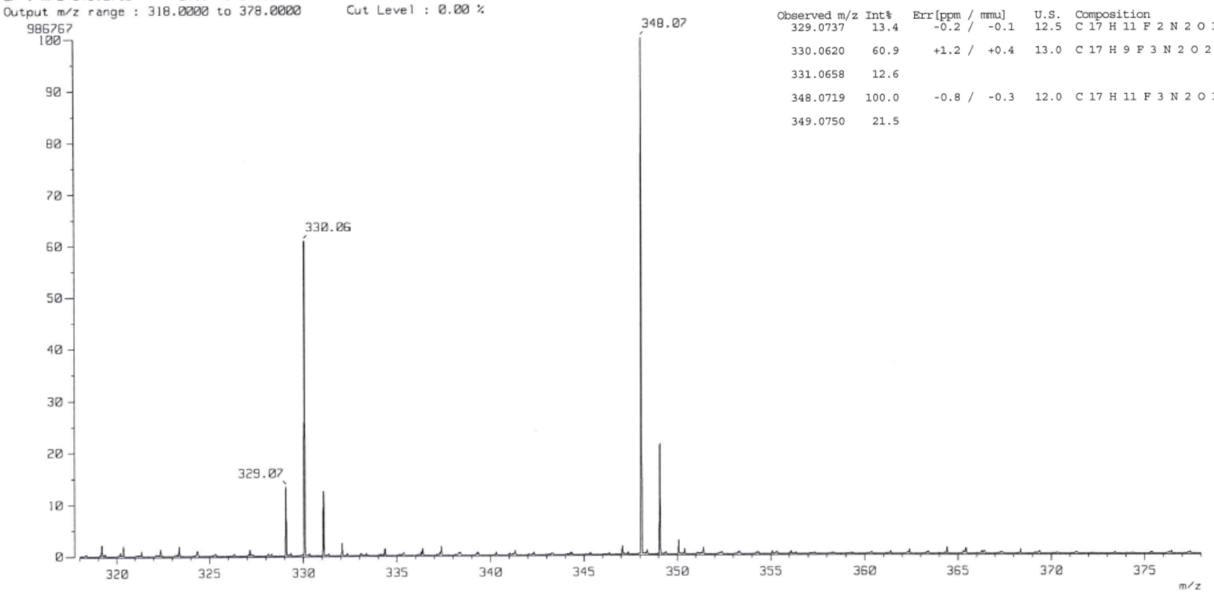

[Mass Spectrum]
Data : KK9304 Date : 15-Mar-2021 16:24
Sample: -
Note : -
Inlet : Direct Ion Mode : EI+
Spectrum Type : Normal Ion [EF-Linear]
RT : 0.95 min Scan# : 20
BP : m/z 348.0719 Int. : 94.11
Output m/z range : 347.8515 to 349.1897 Cut Level : 0.00 %

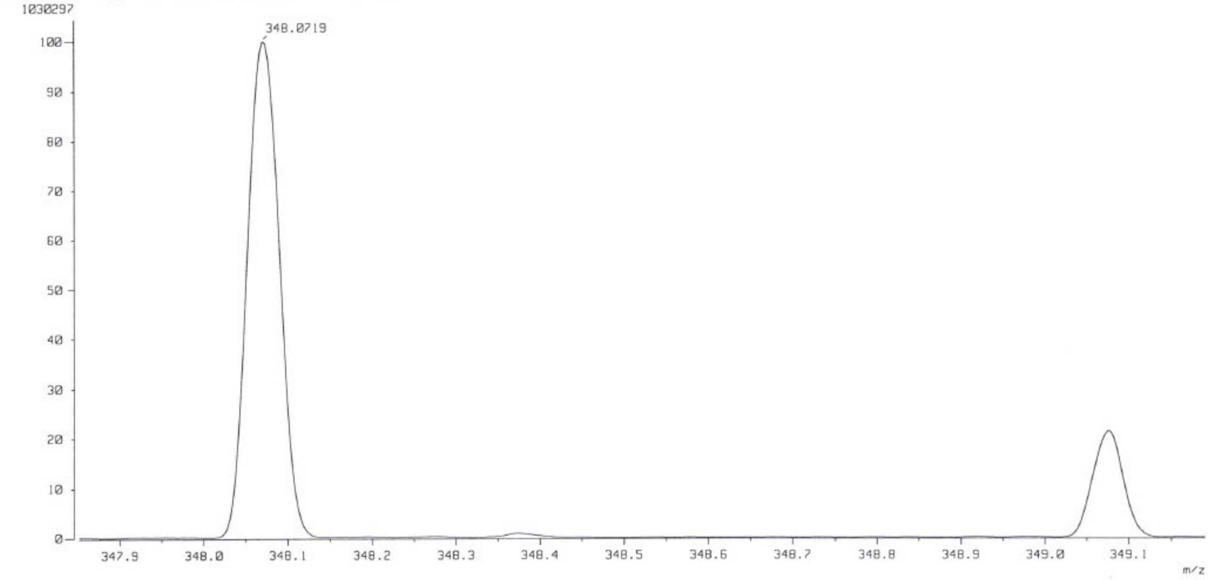

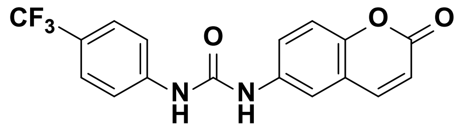

5b

[Mass Spectrum]
Data : KK9305　Date : 15-Mar-2021 16:33
Sample: -
Note : -
Inlet : Direct　Ion Mode : EI+
Spectrum Type : Normal Ion [EF-Linear]
RT : 1.20 min　Scan# : 25
BP : m/z 348.0719　Int. : 56.61
Output m/z range : 318.0000 to 378.0000　Cut Level : 0.00 %

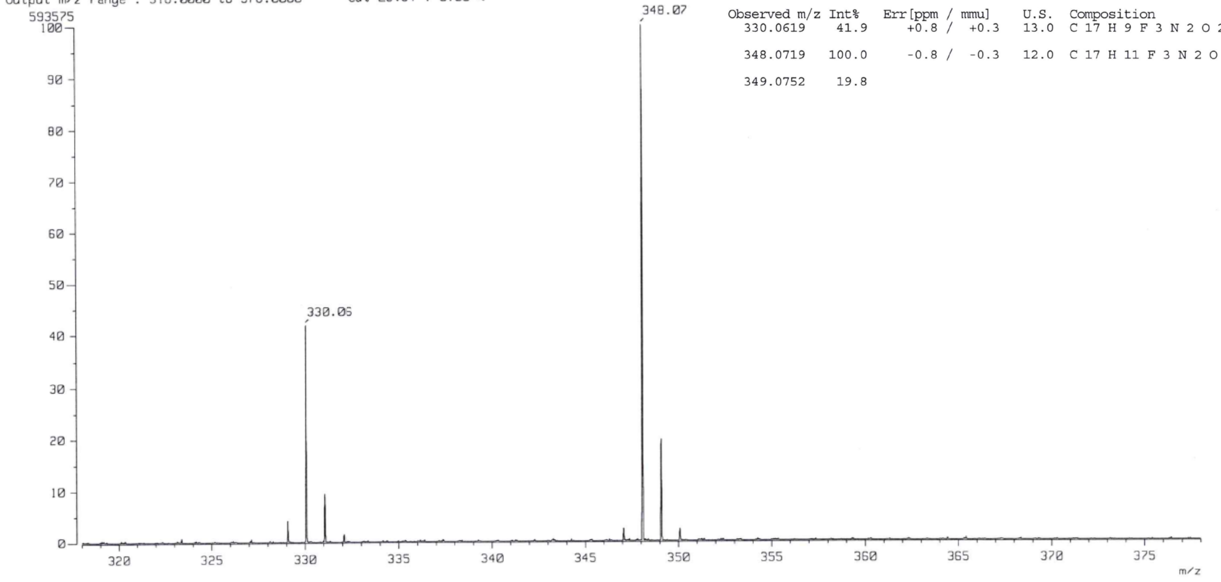

[Elemental Composition]
Data : KK9305　Date : 15-Mar-2021 16:33
Sample: -
Note : -
Inlet : Direct　Ion Mode : EI+
RT : 1.20 min　Scan#: 25
Elements : C 17/0, H 11/0, F 3/0, N 2/0, O 3/0
Mass Tolerance : 1000ppm, 3mmu if m/z > 3
Unsaturation (U.S.) : -0.5 - 100.0

Observed m/z	Int%	Err[ppm / mmu]	U.S.	Composition
330.0619	41.9	+0.8 / +0.3	13.0	C 17 H 9 F 3 N 2 O 2
348.0719	100.0	-0.8 / -0.3	12.0	C 17 H 11 F 3 N 2 O 3
349.0752	19.8			

[Mass Spectrum]
Data : KK9305　Date : 15-Mar-2021 16:33
Sample: -
Note : -
Inlet : Direct　Ion Mode : EI+
Spectrum Type : Normal Ion [EF-Linear]
RT : 1.20 min　Scan# : 25
BP : m/z 348.0719　Int. : 56.61
Output m/z range : 347.7417 to 349.1950　Cut Level : 0.00 %

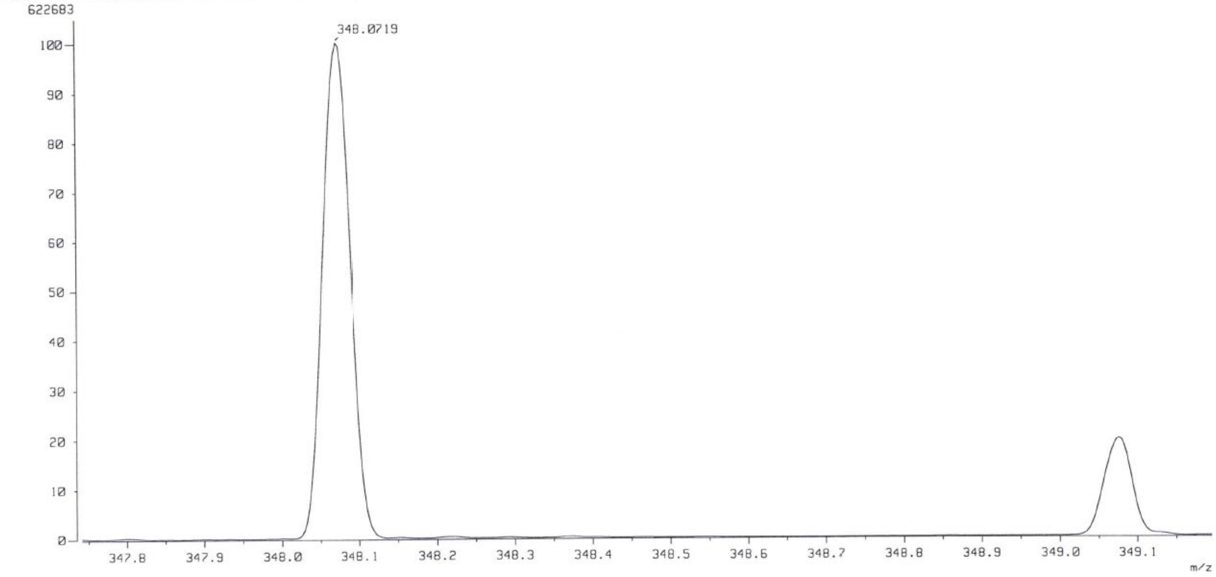

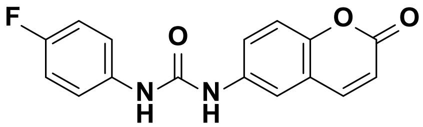

5c

[Mass Spectrum]
Data : KK9308 Date : 17-Mar-2021 15:06
Sample: -
Note : -
Inlet : Direct Ion Mode : EI+
Spectrum Type : Normal Ion [EF-Linear]
RT : 1.10 min Scan# : 34
BP : m/z 298.0753 Int. : 42.93
Output m/z range : 268.0000 to 328.0000 Cut Level : 0.00 %

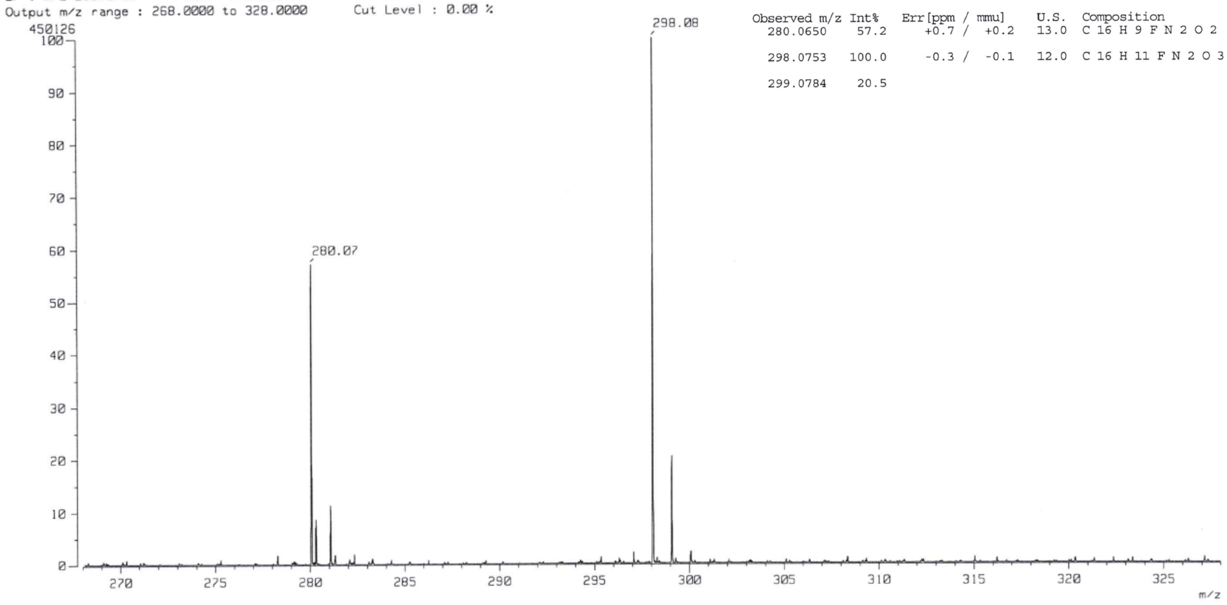

[Elemental Composition]
Data : KK9308 Date : 17-Mar-2021 15:06
Sample: -
Note : -
Inlet : Direct Ion Mode : EI+
RT : 1.10 min Scan#: 34
Elements : C 16/0, H 11/0, F 1/0, N 2/0, O 3/0
Mass Tolerance : 1000ppm, 3mmu if m/z > 3
Unsaturation (U.S.) : -0.5 - 100.0

Observed m/z	Int%	Err[ppm / mmu]	U.S.	Composition
280.0650	57.2	+0.7 / +0.2	13.0	C 16 H 9 F N 2 O 2
298.0753	100.0	-0.3 / -0.1	12.0	C 16 H 11 F N 2 O 3
299.0784	20.5			

[Mass Spectrum]
Data : KK9308 Date : 17-Mar-2021 15:06
Sample: -
Note : -
Inlet : Direct Ion Mode : EI+
Spectrum Type : Normal Ion [EF-Linear]
RT : 1.10 min Scan# : 34
BP : m/z 298.0753 Int. : 42.93
Output m/z range : 297.8135 to 299.2112 Cut Level : 0.00 %

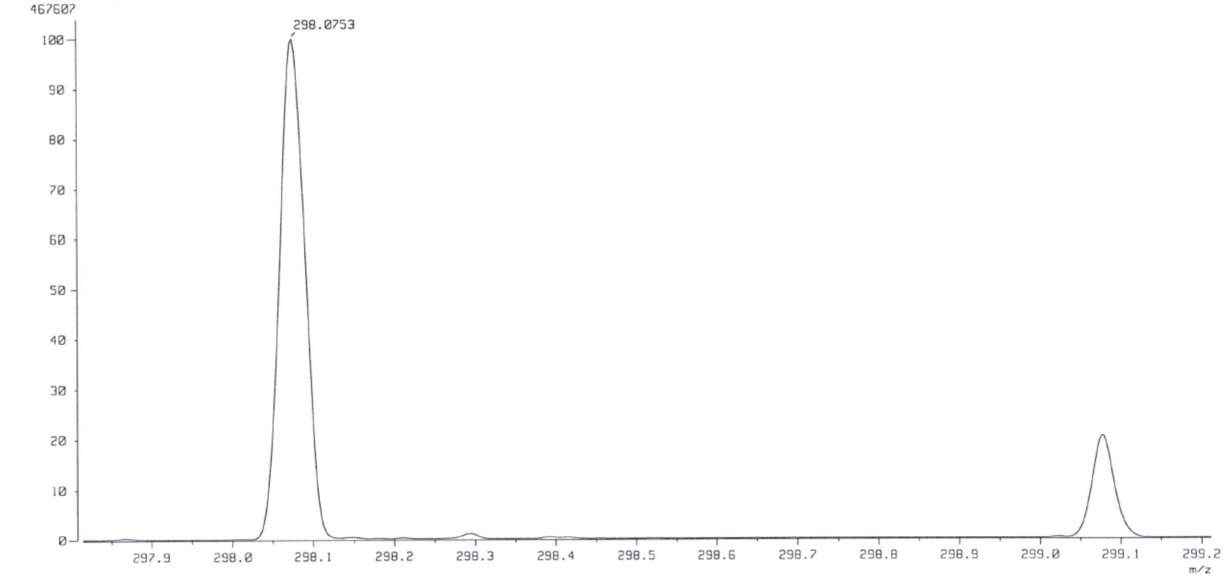

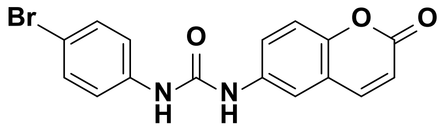

5d

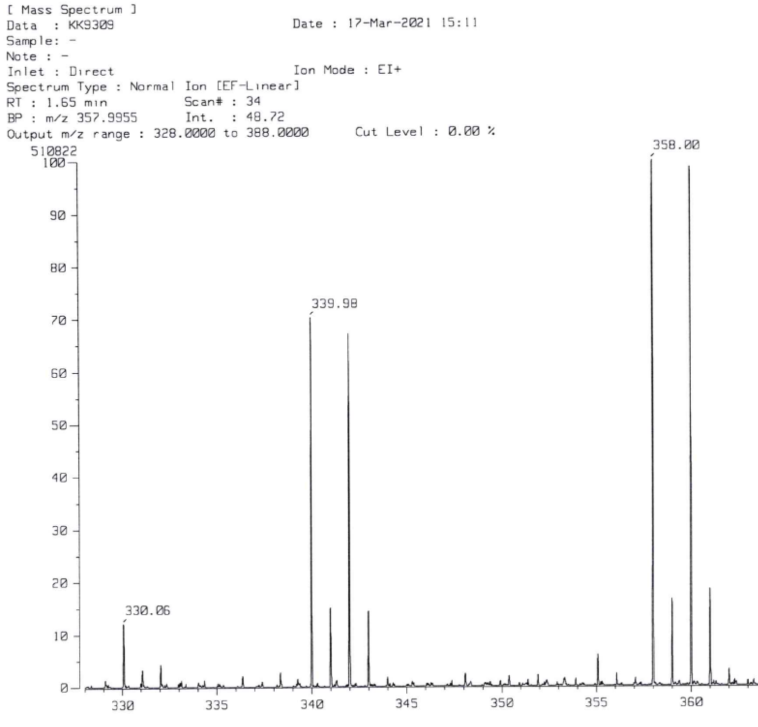

[Elemental Composition]
Data : KK9309 Date : 17-Mar-2021 15:11
Sample: -
Note : -
Inlet : Direct Ion Mode : EI+
RT : 1.65 min Scan#: 34
Elements : C 16/0, H 11/0, Br 1/0, N 2/0, O 3/0
Mass Tolerance : 1000ppm, 1mmu if m/z > 1
Unsaturation (U.S.) : -0.5 - 100.0

Observed m/z	Int%	Err[ppm / mmu]	U.S.	Composition
330.0640	12.1			
339.9845	70.3	-0.7 / -0.2	13.0	C 16 H 9 Br N 2 O 2
340.9906	15.1			
341.9835	67.2			
342.9874	14.4			
357.9955	100.0	+0.7 / +0.2	12.0	C 16 H 11 Br N 2 O 3
358.9987	16.6			
359.9938	98.7			
360.9965	18.5			
369.9162	12.4			

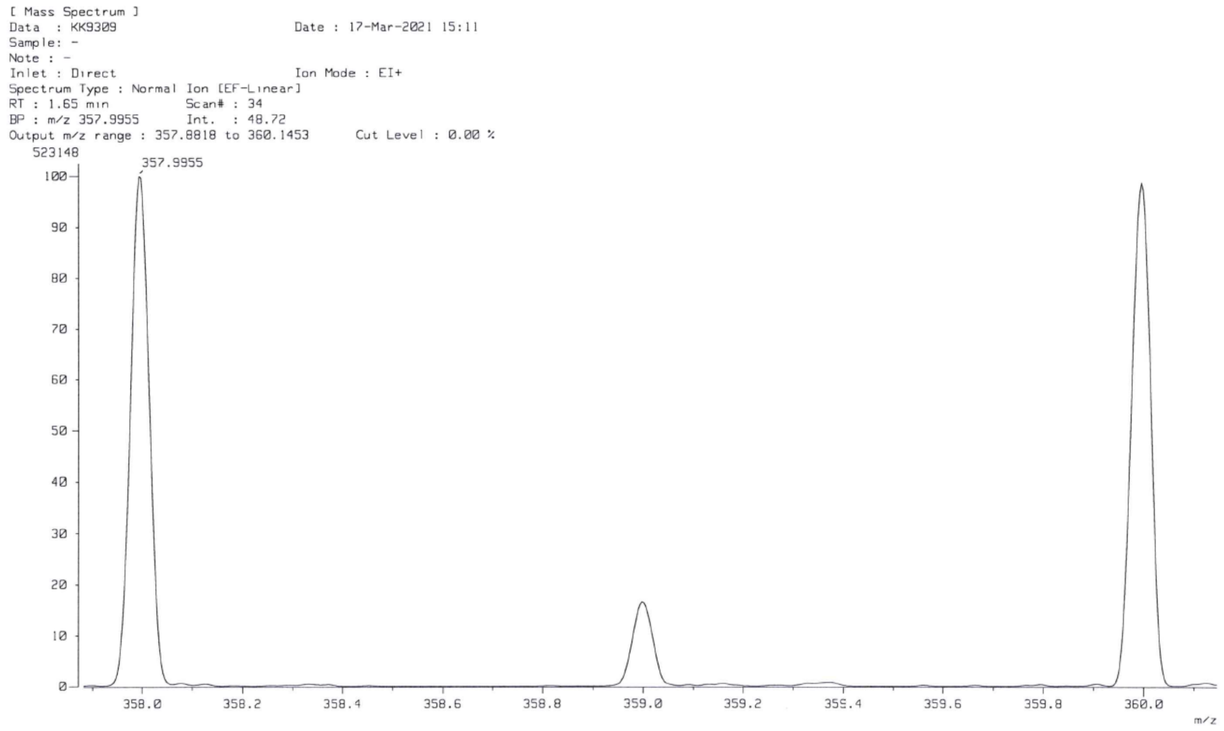

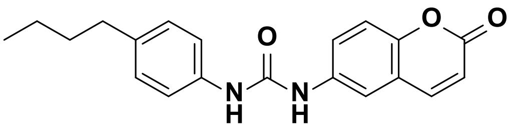

5e

[Mass Spectrum]
Data : KK9310 Date : 17-Mar-2021 15:16
Sample: -
Note : -
Inlet : Direct Ion Mode : EI+
Spectrum Type : Normal Ion [EF-Linear]
RT : 0.75 min Scan# : 16
BP : m/z 336.1472 Int. : 86.34
Output m/z range : 306.0000 to 366.0000 Cut Level : 0.00 %

[Elemental Composition]
Data : KK9310 Date : 17-Mar-2021 15:16
Sample: -
Note : -
Inlet : Direct Ion Mode : EI+
RT : 0.75 min Scan#: 16
Elements : C 20/0, H 20/0, N 2/0, O 3/0
Mass Tolerance : 1000ppm, 3mmu if m/z > 3
Unsaturation (U.S.) : -0.5 - 100.0

Observed m/z	Int%	Err[ppm / mmu]	U.S.	Composition
318.1371	36.5	+0.8 / +0.3	13.0	C 20 H 18 N 2 O 2
324.2213	15.2			
336.1472	100.0	-0.6 / -0.2	12.0	C 20 H 20 N 2 O 3
337.1508	23.8			

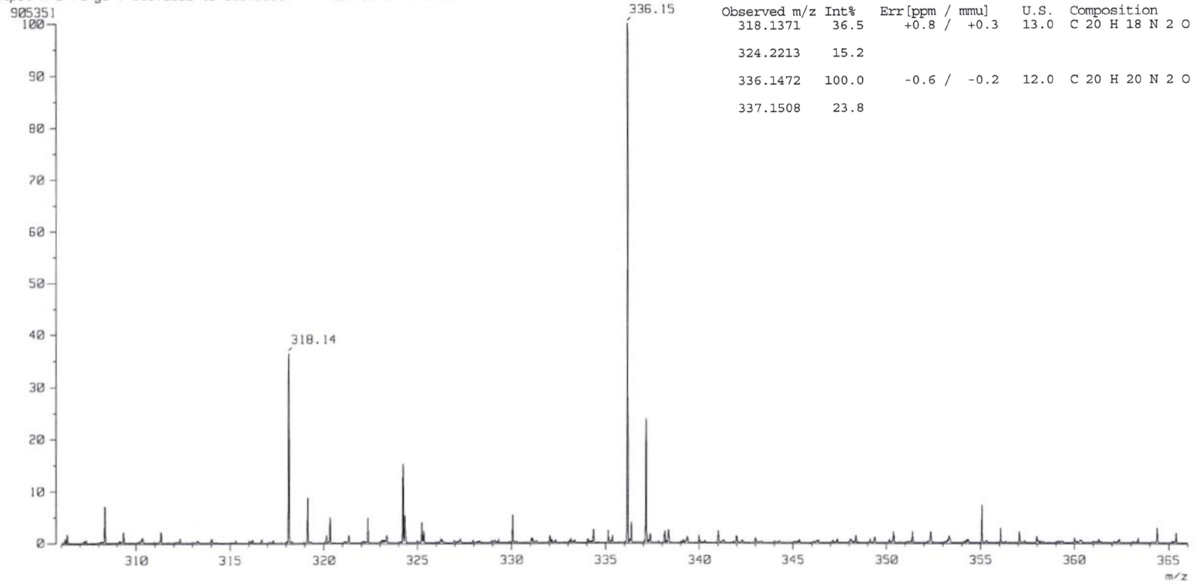

[Mass Spectrum]
Data : KK9310 Date : 17-Mar-2021 15:16
Sample: -
Note : -
Inlet : Direct Ion Mode : EI+
Spectrum Type : Normal Ion [EF-Linear]
RT : 0.75 min Scan# : 16
BP : m/z 336.1472 Int. : 86.34
Output m/z range : 335.8093 to 337.3261 Cut Level : 0.00 %

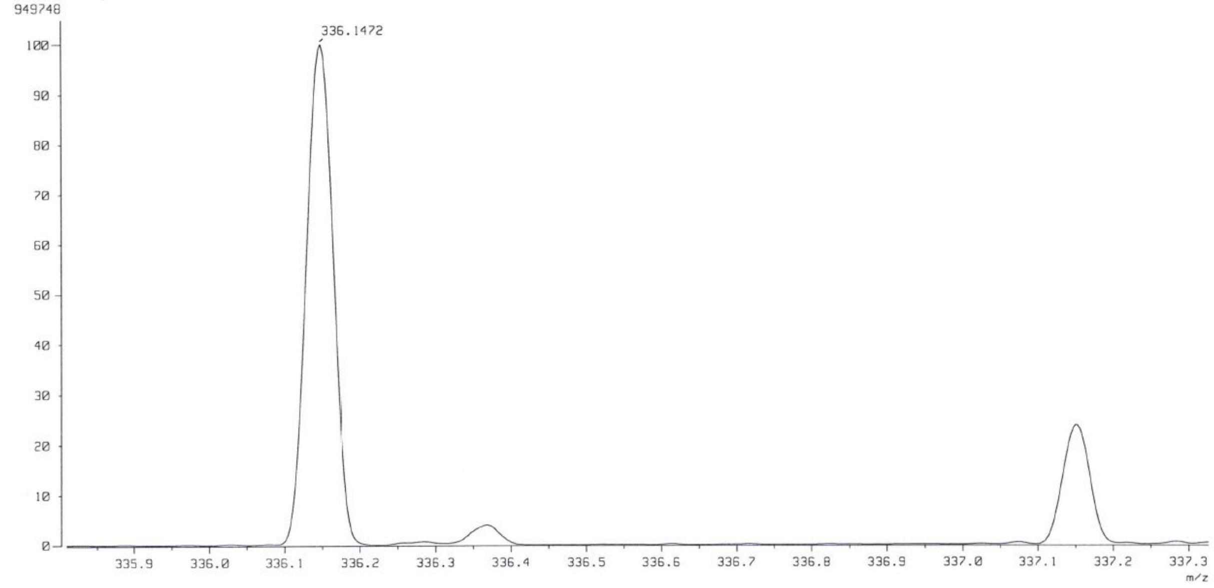

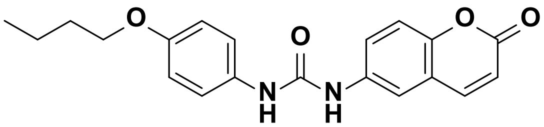

5f

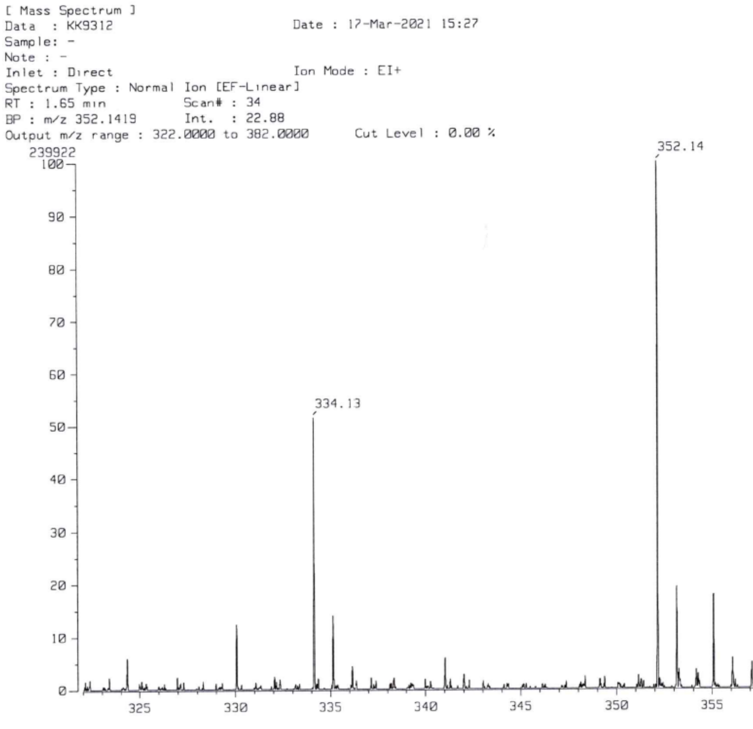

[Elemental Composition]
Data : KK9312 Date : 17-Mar-2021 15:27
Sample: -
Note : -
Inlet : Direct Ion Mode : EI+
RT : 1.65 min Scan#: 34
Elements : C 20/0, H 20/0, N 2/0, O 4/0
Mass Tolerance : 1000ppm, 3mmu if m/z > 3
Unsaturation (U.S.) : -0.5 - 100.0

Observed m/z	Int%	Err[ppm / mmu]	U.S.	Composition
334.1317	51.6	+0.0 / +0.0	13.0	C 20 H 18 N 2 O 3
352.1419	100.0	-1.1 / -0.4	12.0	C 20 H 20 N 2 O 4

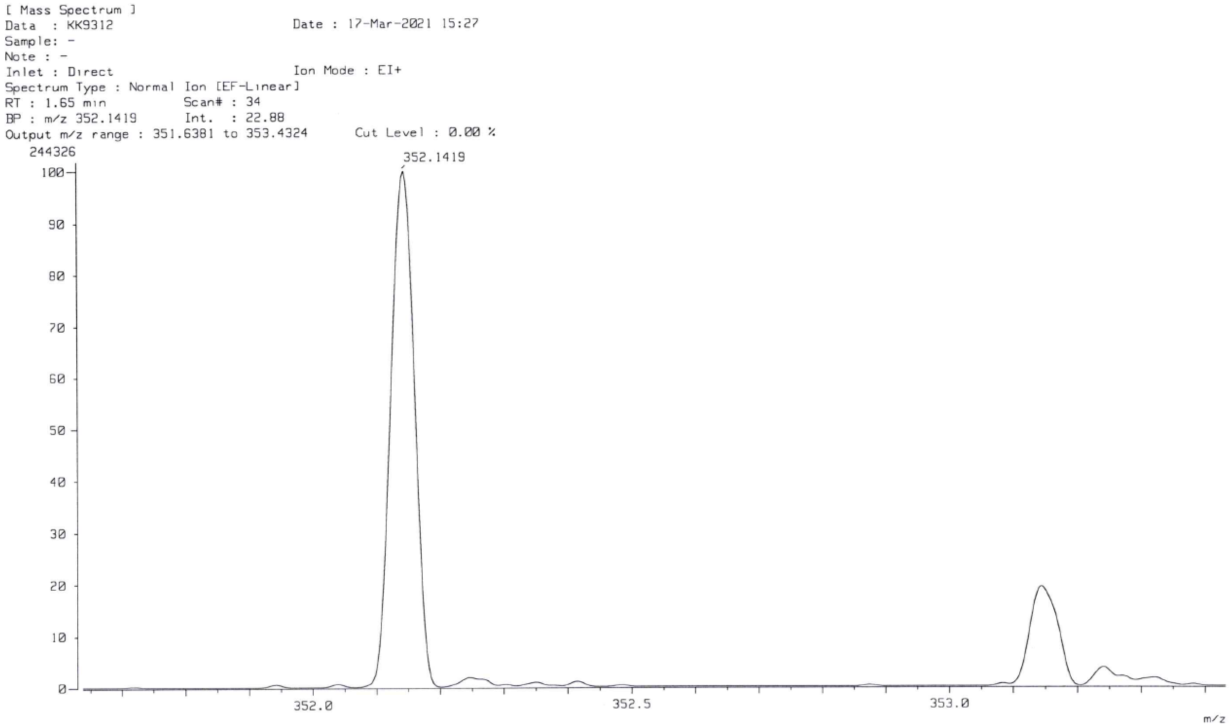

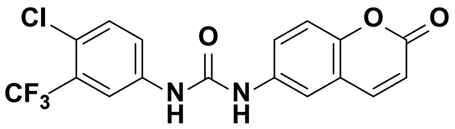

5g

[Mass Spectrum]
Data : KK9301 Date : 15-Mar-2021 15:54
Sample: -
Note : -
Inlet : Direct Ion Mode : EI+
Spectrum Type : Normal Ion [EF-Linear]
RT : 1.25 min Scan# : 26
BP : m/z 382.0329 Int. : 34.90
Output m/z range : 352.0000 to 412.0000 Cut Level : 0.00 %

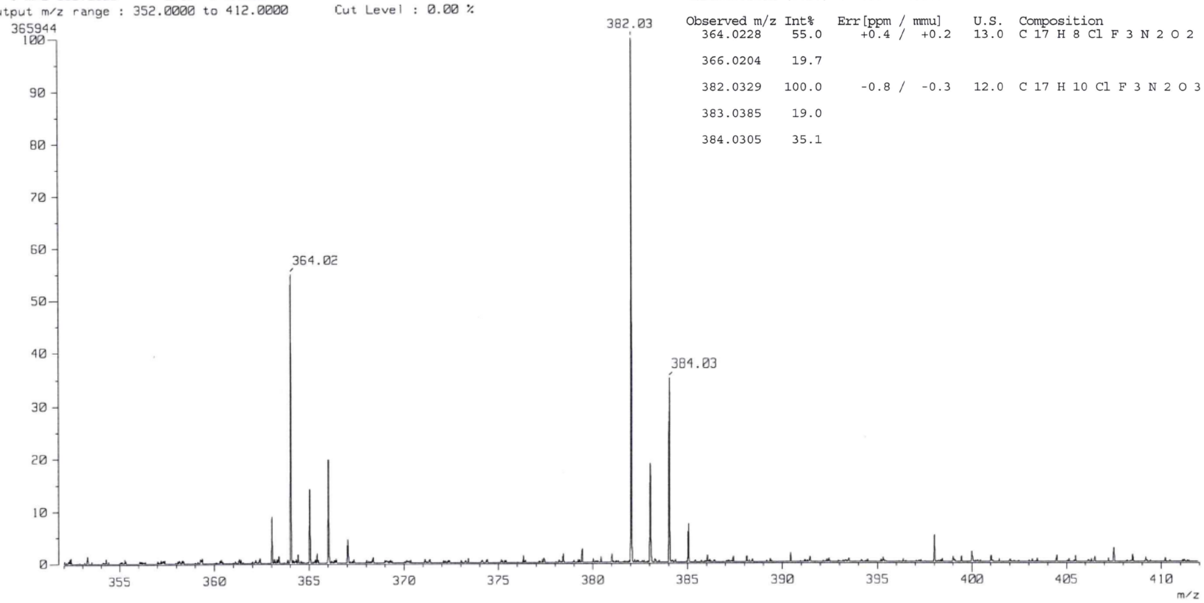

[Elemental Composition]
Data : KK9301 Date : 15-Mar-2021 15:54
Sample: -
Note : -
Inlet : Direct Ion Mode : EI+
RT : 1.25 min Scan#: 26
Elements : C 17/0, H 10/0, Cl 1/0, F 3/0, N 2/0, O 3/0
Mass Tolerance : 1000ppm, 3mmu if m/z > 3
Unsaturation (U.S.) : -0.5 - 100.0

Observed m/z	Int%	Err[ppm / mmu]	U.S.	Composition
364.0228	55.0	+0.4 / +0.2	13.0	C 17 H 8 Cl F 3 N 2 O 2
366.0204	19.7			
382.0329	100.0	-0.8 / -0.3	12.0	C 17 H 10 Cl F 3 N 2 O 3
383.0385	19.0			
384.0305	35.1			

[Mass Spectrum]
Data : KK9301 Date : 15-Mar-2021 15:54
Sample: -
Note : -
Inlet : Direct Ion Mode : EI+
Spectrum Type : Normal Ion [EF-Linear]
RT : 1.25 min Scan# : 26
BP : m/z 382.0329 Int. : 34.90
Output m/z range : 381.8807 to 384.0858 Cut Level : 0.00 %

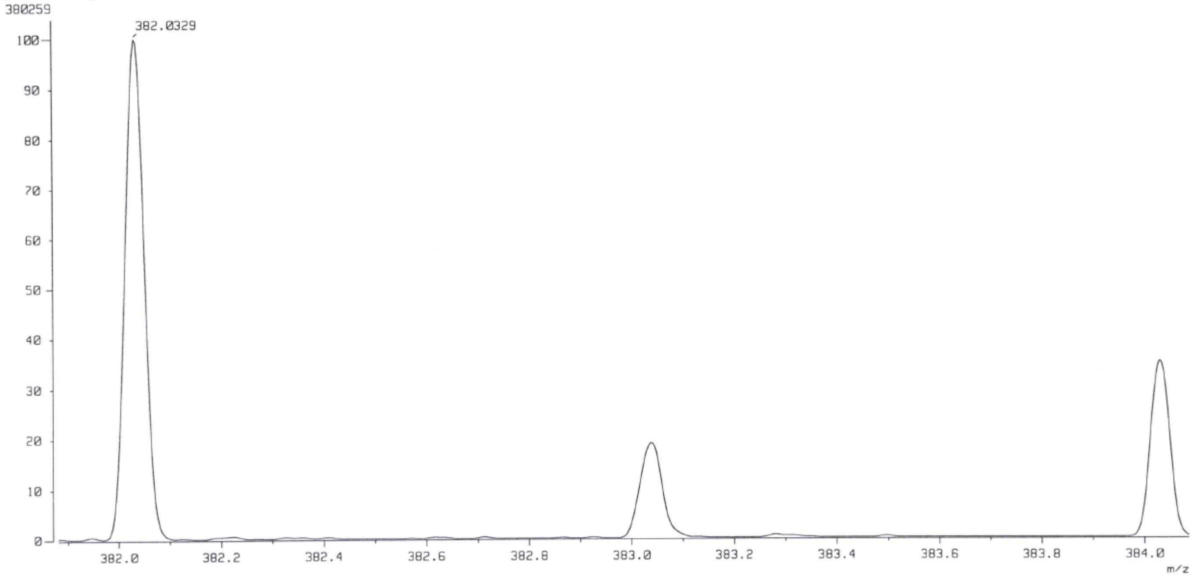

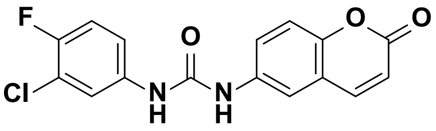

5h

[Mass Spectrum]
Data : KK9303 Date : 15-Mar-2021 16:11
Sample: -
Note : -
Inlet : Direct Ion Mode : EI+
Spectrum Type : Normal Ion [EF-Linear]
RT : 1.85 min Scan# : 38
BP : m/z 332.0361 Int. : 75.07
Output m/z range : 302.0000 to 362.0000 Cut Level : 0.00 %

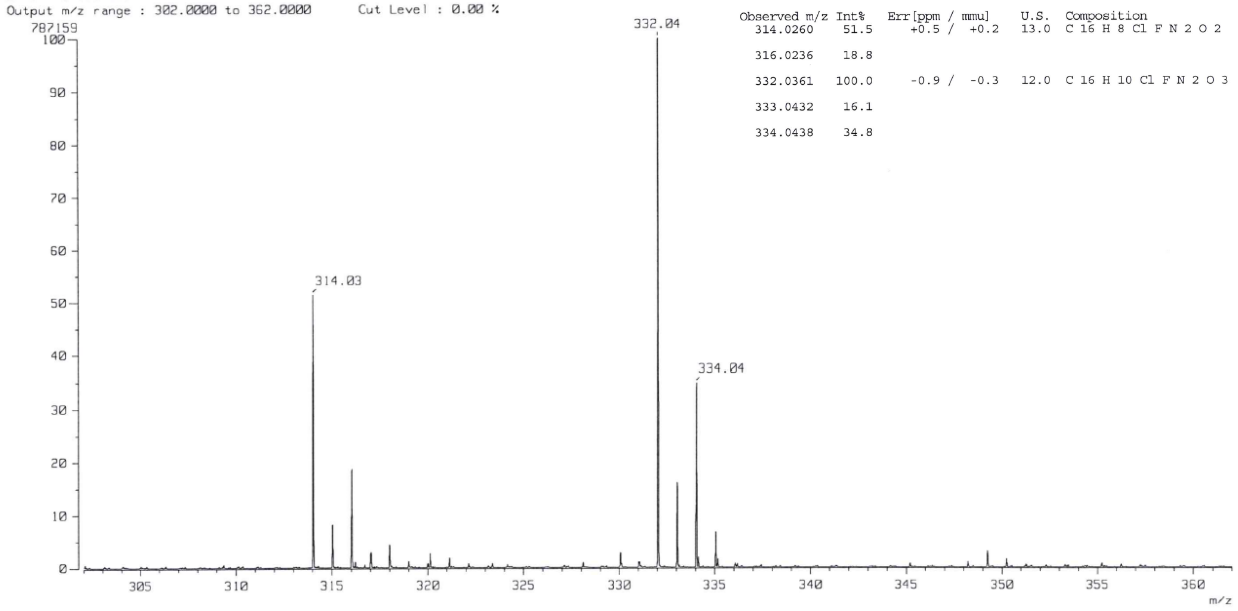

[Elemental Composition]
Data : KK9303 Date : 15-Mar-2021 16:11
Sample: -
Note : -
Inlet : Direct Ion Mode : EI+
RT : 1.85 min Scan#: 38
Elements : C 16/0, H 10/0, Cl 1/0, F 1/0, N 2/0, O 3/0
Mass Tolerance : 1000ppm, 3mmu if m/z > 3
Unsaturation (U.S.) : -0.5 - 100.0

Observed m/z	Int%	Err[ppm / mmu]	U.S.	Composition
314.0260	51.5	+0.5 / +0.2	13.0	C 16 H 8 Cl F N 2 O 2
316.0236	18.8			
332.0361	100.0	-0.9 / -0.3	12.0	C 16 H 10 Cl F N 2 O 3
333.0432	16.1			
334.0438	34.8			

[Mass Spectrum]
Data : KK9303 Date : 15-Mar-2021 16:11
Sample: -
Note : -
Inlet : Direct Ion Mode : EI+
Spectrum Type : Normal Ion [EF-Linear]
RT : 1.85 min Scan# : 38
BP : m/z 332.0361 Int. : 75.07
Output m/z range : 331.9152 to 334.1088 Cut Level : 0.00 %

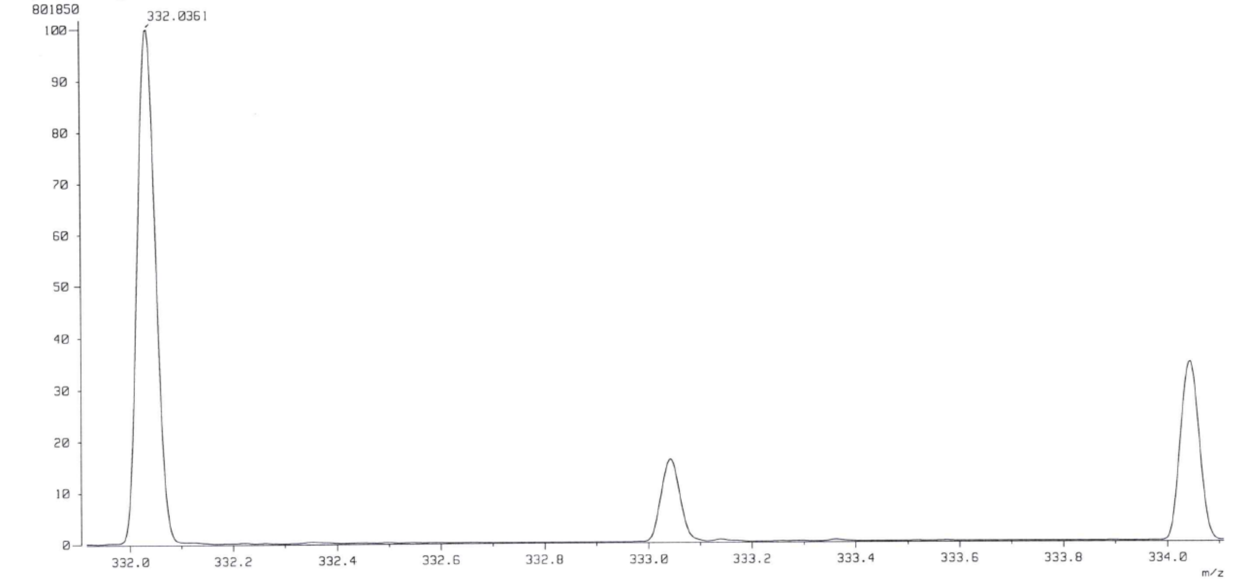

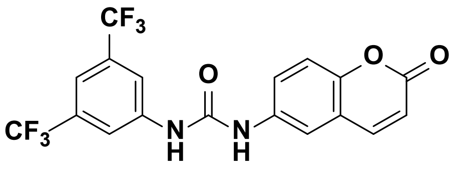

5i

[Mass Spectrum]
Data : KK9302
Date : 15-Mar-2021 16:03
Sample: -
Note : -
Inlet : Direct
Ion Mode : EI+
Spectrum Type : Normal Ion [EF-Linear]
RT : 0.95 min
Scan# : 20
BP : m/z 398.0492
Int. : 44.37
Output m/z range : 386.0000 to 446.0000
Cut Level : 0.00 %

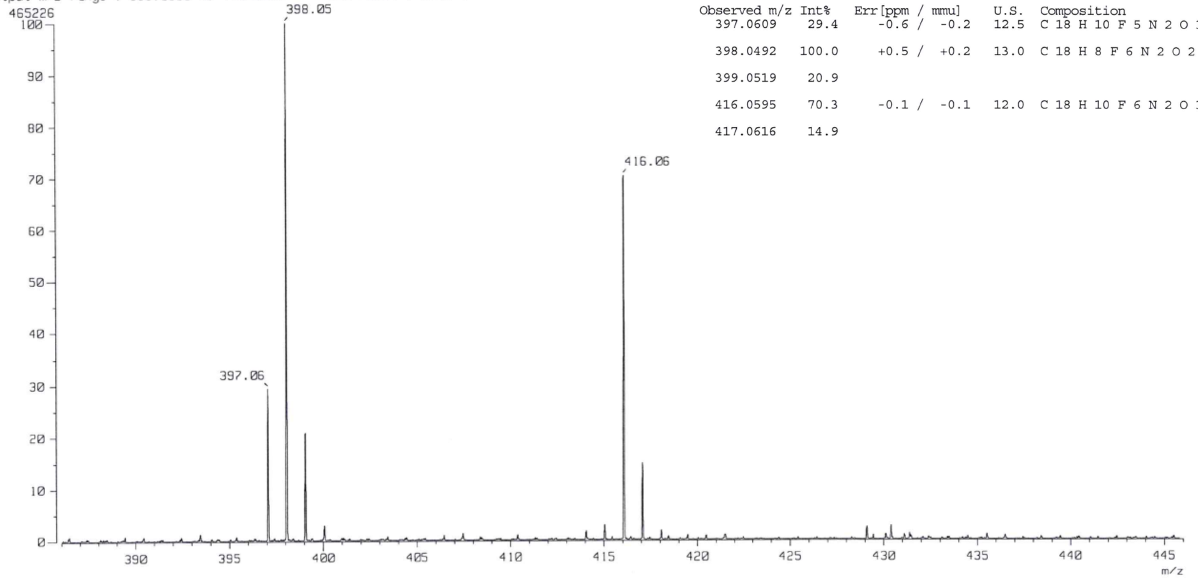

[Elemental Composition]
Data : KK9302
Date : 15-Mar-2021 16:03
Sample: -
Note : -
Inlet : Direct
Ion Mode : EI+
RT : 0.95 min
Scan#: 20
Elements : C 18/0, H 10/0, F 6/0, N 2/0, O 3/0
Mass Tolerance : 1000ppm, 3mmu if m/z > 3
Unsaturation (U.S.) : -0.5 - 100.0

Observed m/z	Int%	Err[ppm / mmu]	U.S.	Composition
397.0609	29.4	-0.6 / -0.2	12.5	C 18 H 10 F 5 N 2 O 3
398.0492	100.0	+0.5 / +0.2	13.0	C 18 H 8 F 6 N 2 O 2
399.0519	20.9			
416.0595	70.3	-0.1 / -0.1	12.0	C 18 H 10 F 6 N 2 O 3
417.0616	14.9			

[Mass Spectrum]
Data : KK9302
Date : 15-Mar-2021 16:03
Sample: -
Note : -
Inlet : Direct
Ion Mode : EI+
Spectrum Type : Normal Ion [EF-Linear]
RT : 0.95 min
Scan# : 20
BP : m/z 398.0492
Int. : 44.37
Output m/z range : 415.7197 to 417.2586
Cut Level : 0.00 %

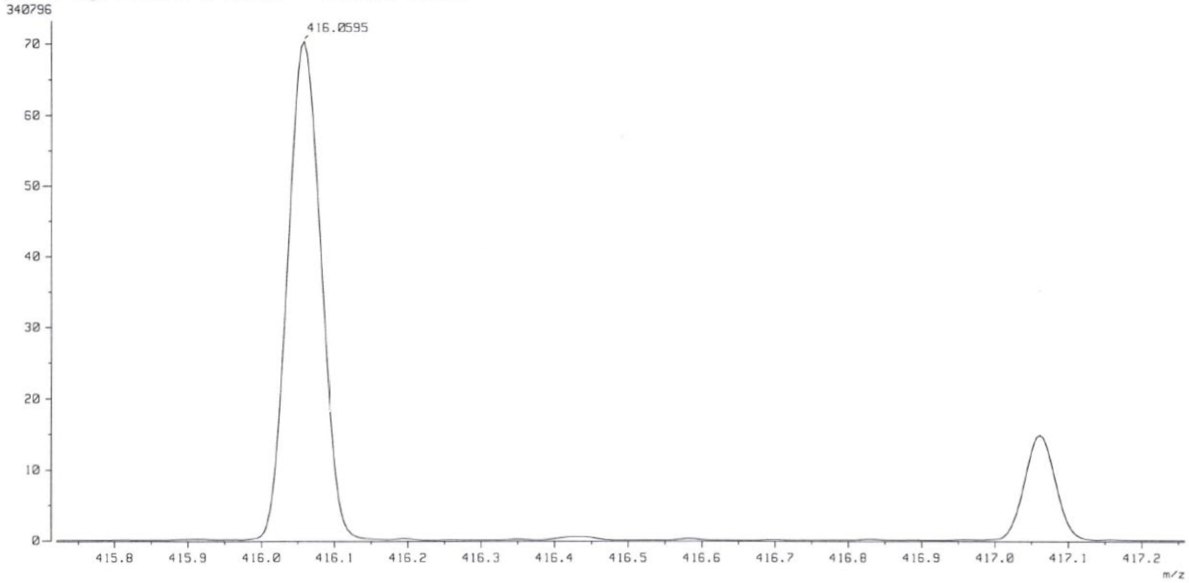

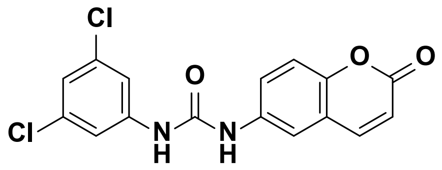

5j

[Mass Spectrum]
Data : KK9306 Date : 15-Mar-2021 16:39
Sample: -
Note : -
Inlet : Direct Ion Mode : EI+
Spectrum Type : Normal Ion [EF-Linear]
RT : 1.75 min Scan# : 36
BP : m/z 348.0064 Int. : 45.78
Output m/z range : 318.0000 to 378.0000 Cut Level : 0.00 %

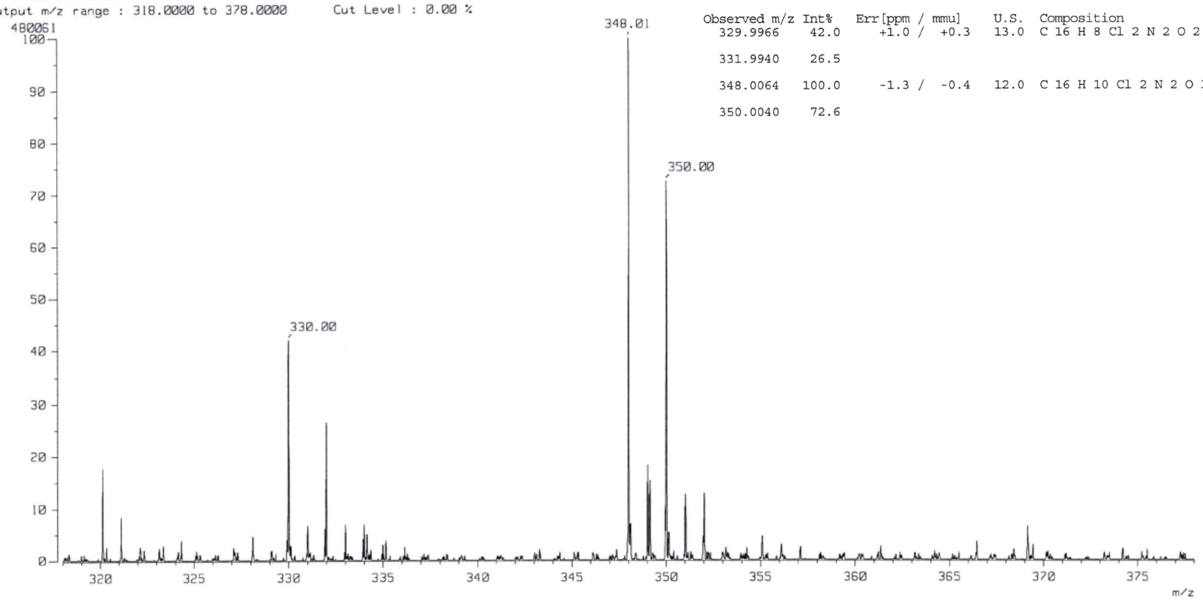

[Elemental Composition]
Data : KK9306 Date : 15-Mar-2021 16:39
Sample: -
Note : -
Inlet : Direct Ion Mode : EI+
RT : 1.75 min Scan#: 36
Elements : C 16/0, H 10/0, Cl 2/0, N 2/0, O 3/0
Mass Tolerance : 1000ppm, 3mmu if m/z > 3
Unsaturation (U.S.) : -0.5 - 100.0

Observed m/z	Int%	Err[ppm / mmu]	U.S.	Composition
329.9966	42.0	+1.0 / +0.3	13.0	C 16 H 8 Cl 2 N 2 O 2
331.9940	26.5			
348.0064	100.0	-1.3 / -0.4	12.0	C 16 H 10 Cl 2 N 2 O 3
350.0040	72.6			

[Mass Spectrum]
Data : KK9306 Date : 15-Mar-2021 16:39
Sample: -
Note : -
Inlet : Direct Ion Mode : EI+
Spectrum Type : Normal Ion [EF-Linear]
RT : 1.75 min Scan# : 36
BP : m/z 348.0064 Int. : 45.78
Output m/z range : 347.8197 to 350.0789 Cut Level : 0.00 %

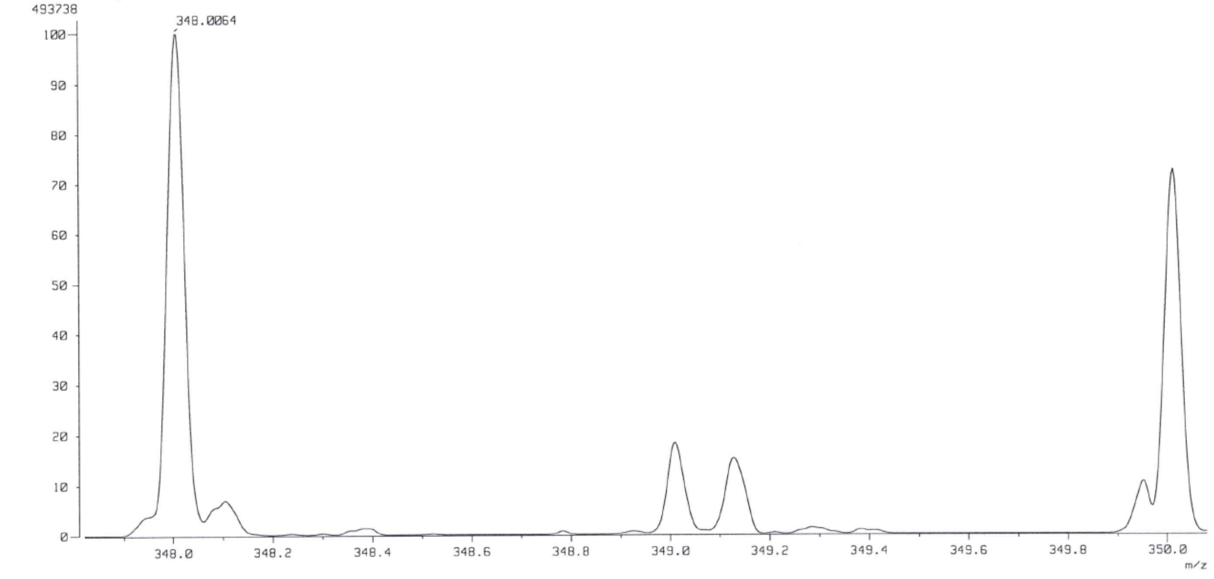

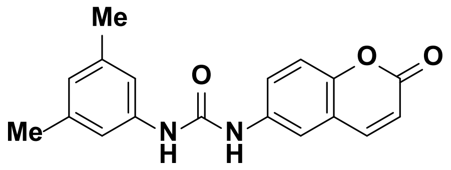

5k

[Mass Spectrum]
Data : KK9311 Date : 17-Mar-2021 15:22
Sample: -
Note : -
Inlet : Direct Ion Mode : EI+
Spectrum Type : Normal Ion [EF-Linear]
RT : 0.70 min Scan# : 15
BP : m/z 308.1158 Int. : 48.76
Output m/z range : 283.0742 to 336.3086 Cut Level : 0.00 %

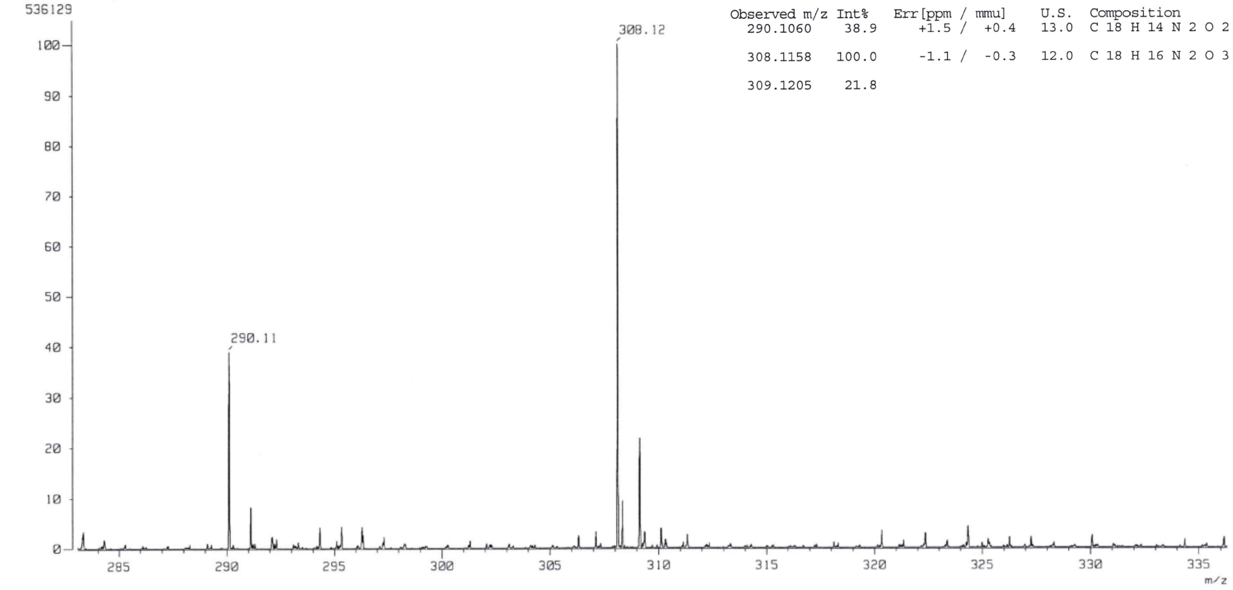

[Elemental Composition]
Data : KK9311 Date : 17-Mar-2021 15:22
Sample: -
Note : -
Inlet : Direct Ion Mode : EI+
RT : 0.70 min Scan#: 15
Elements : C 18/0, H 16/0, N 2/0, O 3/0
Mass Tolerance : 1000ppm, 3mmu if m/z > 3
Unsaturation (U.S.) : -0.5 - 100.0

Observed m/z	Int%	Err[ppm / mmu]	U.S.	Composition
290.1060	38.9	+1.5 / +0.4	13.0	C 18 H 14 N 2 O 2
308.1158	100.0	-1.1 / -0.3	12.0	C 18 H 16 N 2 O 3
309.1205	21.8			

[Mass Spectrum]
Data : KK9311 Date : 17-Mar-2021 15:22
Sample: -
Note : -
Inlet : Direct Ion Mode : EI+
Spectrum Type : Normal Ion [EF-Linear]
RT : 0.70 min Scan# : 15
BP : m/z 308.1158 Int. : 48.76
Output m/z range : 307.6836 to 309.4191 Cut Level : 0.00 %

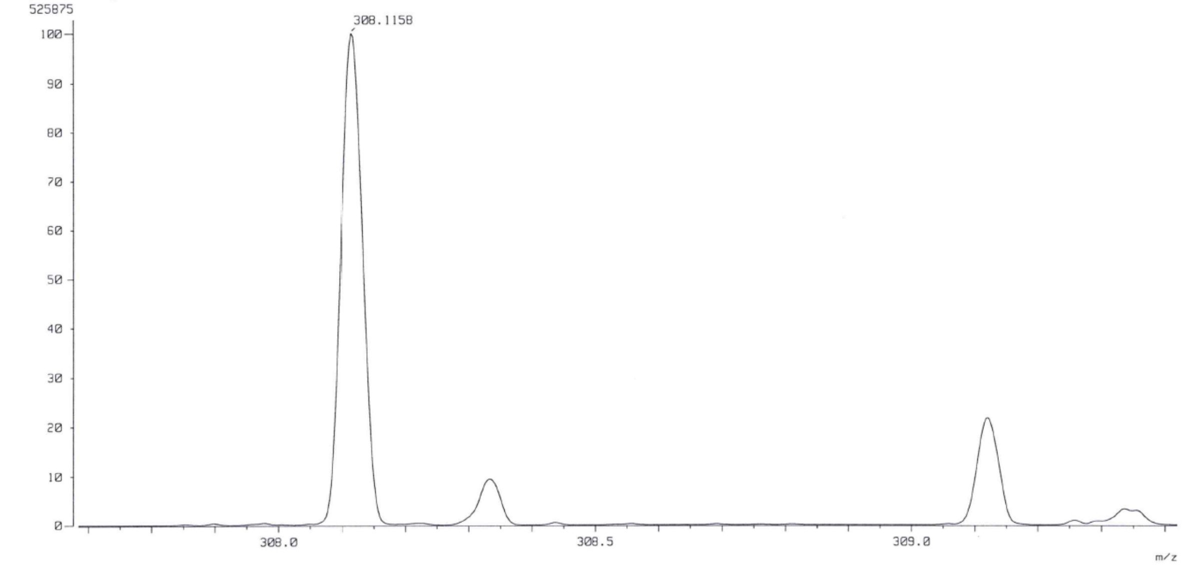

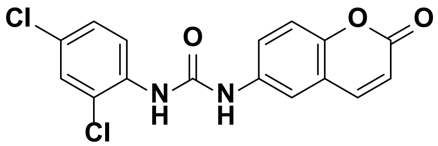

5l

[Mass Spectrum]
Data : KK9307 Date : 15-Mar-2021 16:47
Sample: -
Note : -
Inlet : Direct Ion Mode : EI+
Spectrum Type : Normal Ion [EF-Linear]
RT : 1.50 min Scan# : 31
BP : m/z 348.0065 Int. : 79.93
Output m/z range : 318.0000 to 378.0000 Cut Level : 0.00 %

[Elemental Composition]
Data : KK9307 Date : 15-Mar-2021 16:47
Sample: -
Note : -
Inlet : Direct Ion Mode : EI+
RT : 1.50 min Scan#: 31
Elements : C 16/0, H 10/0, Cl 2/0, N 2/0, O 3/0
Mass Tolerance : 1000ppm, 3mmu if m/z > 3
Unsaturation (U.S.) : -0.5 - 100.0

Observed m/z	Int%	Err[ppm / mmu]	U.S.	Composition
329.9967	30.5	+1.3 / +0.4	13.0	C 16 H 8 Cl 2 N 2 O 2
331.9935	20.2			
348.0065	100.0	-1.0 / -0.3	12.0	C 16 H 10 Cl 2 N 2 O 3
349.0108	18.6			
350.0038	62.4			

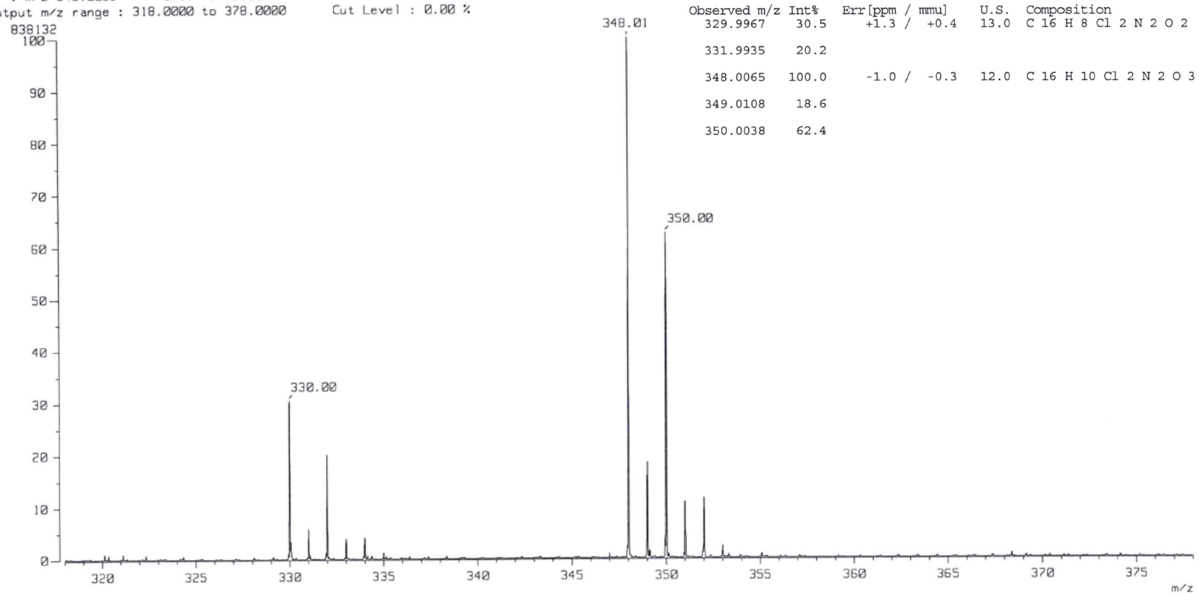

[Mass Spectrum]
Data : KK9307 Date : 15-Mar-2021 16:47
Sample: -
Note : -
Inlet : Direct Ion Mode : EI+
Spectrum Type : Normal Ion [EF-Linear]
RT : 1.50 min Scan# : 31
BP : m/z 348.0065 Int. : 79.93
Output m/z range : 347.8052 to 350.0637 Cut Level : 0.00 %

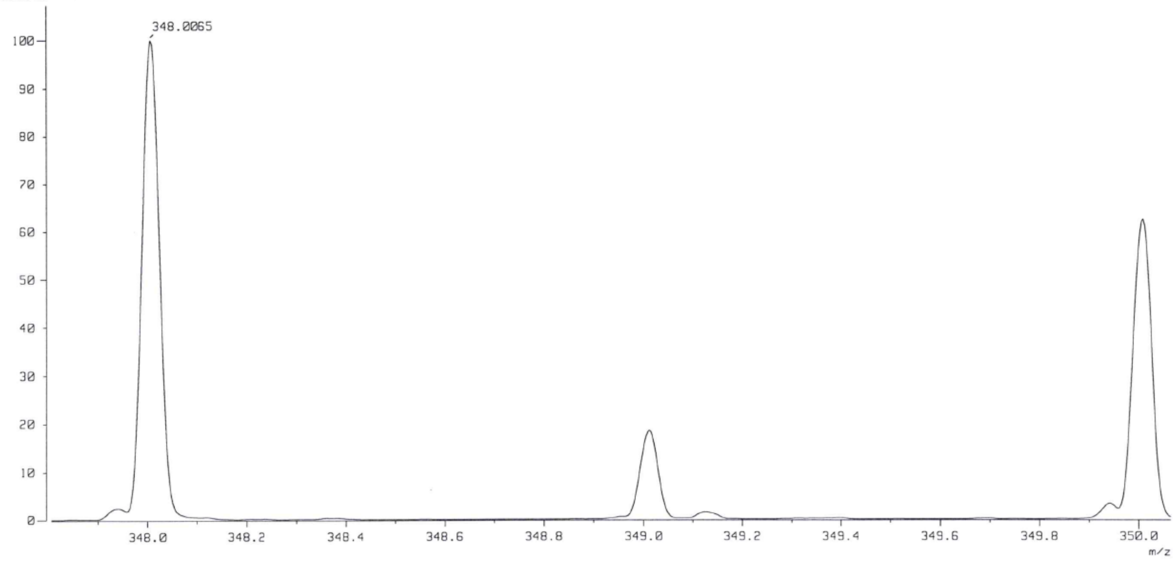

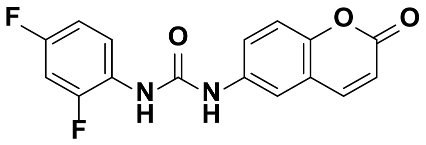

5m

[Mass Spectrum]
Data : KK9315 Date : 17-Mar-2021 15:42
Sample: -
Note : -
Inlet : Direct Ion Mode : EI+
Spectrum Type : Normal Ion [EF-Linear]
RT : 1.65 min Scan# : 34
BP : m/z 316.0660 Int. : 37.16
Output m/z range : 286.0000 to 346.0000 Cut Level : 0.00 %

[Elemental Composition]
Data : KK9315 Date : 17-Mar-2021 15:42
Sample: -
Note : -
Inlet : Direct Ion Mode : EI+
RT : 1.65 min Scan#: 34
Elements : C 16/0, H 10/0, F 2/0, N 2/0, O 3/0
Mass Tolerance : 1000ppm, 3mmu if m/z > 3
Unsaturation (U.S.) : -0.5 - 100.0

Observed m/z	Int%	Err[ppm / mmu]	U.S.	Composition
298.0551	33.5	-0.9 / -0.3	13.0	C 16 H 8 F 2 N 2 O 2
316.0660	100.0	+0.2 / +0.1	12.0	C 16 H 10 F 2 N 2 O 3

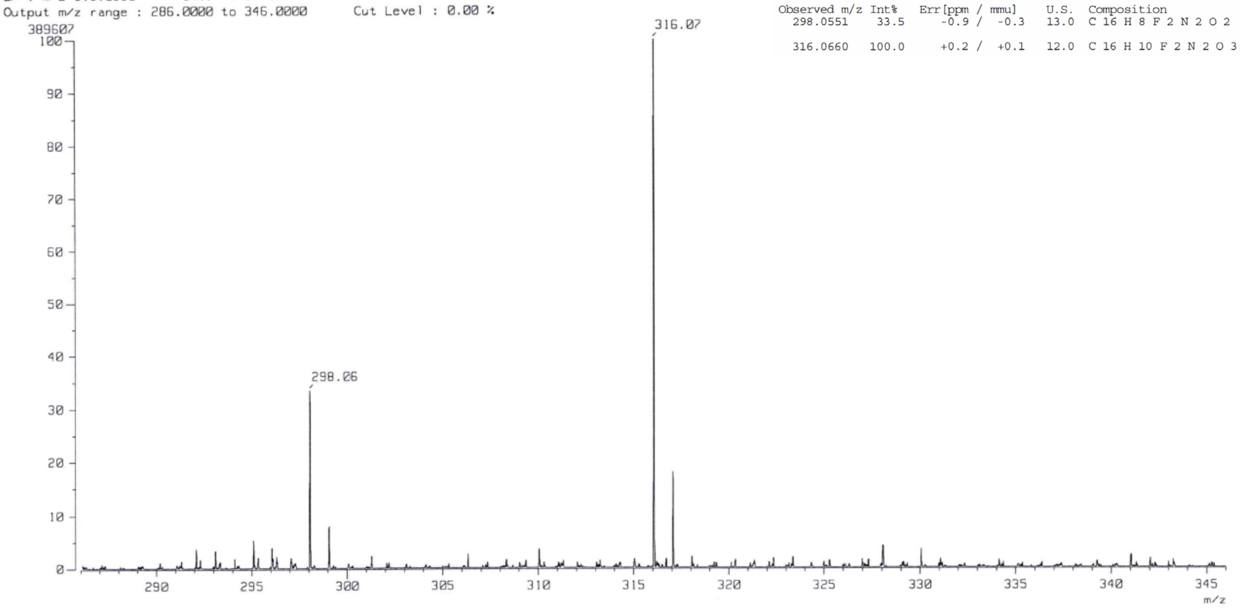

[Mass Spectrum]
Data : KK9315 Date : 17-Mar-2021 15:42
Sample: -
Note : -
Inlet : Direct Ion Mode : EI+
Spectrum Type : Normal Ion [EF-Linear]
RT : 1.65 min Scan# : 34
BP : m/z 316.0660 Int. : 37.16
Output m/z range : 315.6995 to 317.1466 Cut Level : 0.00 %

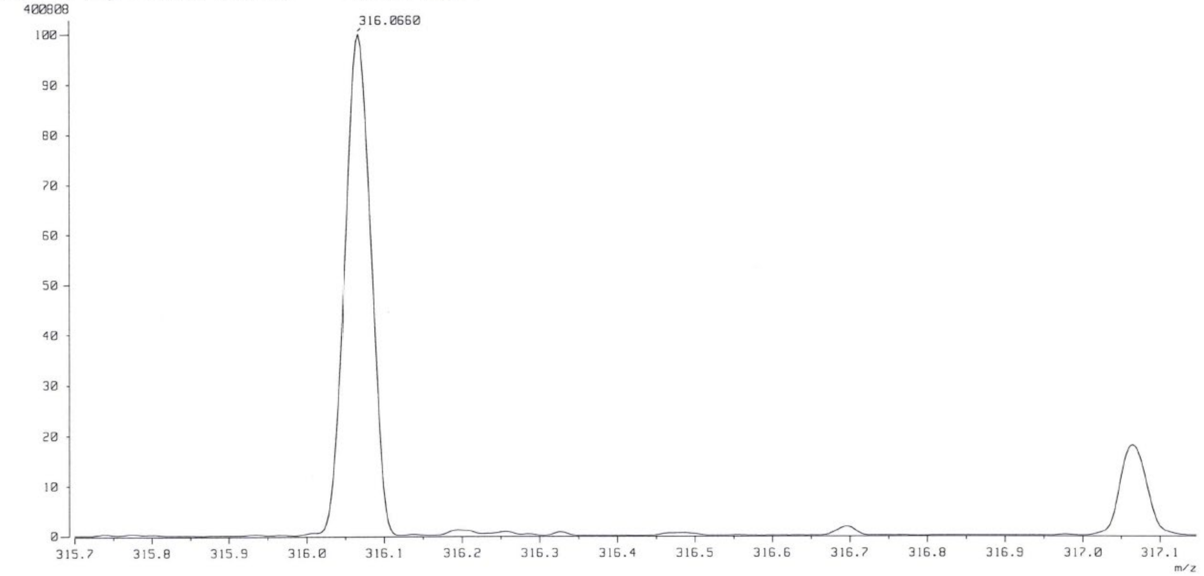

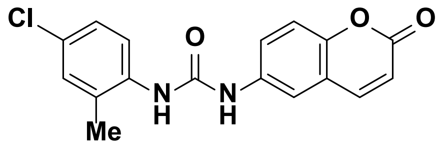
Cl
O
O
O
N
H
N
H
Me

5n

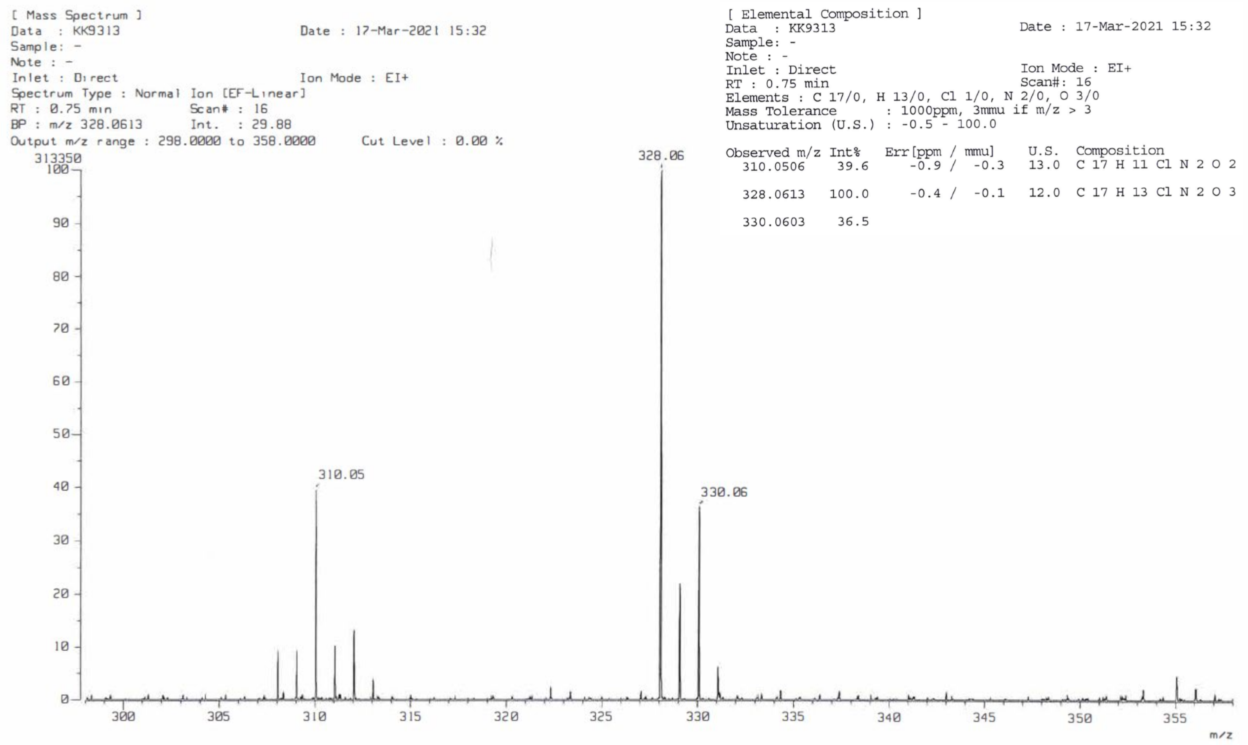
[Mass Spectrum]
Data : KK9313
Date : 17-Mar-2021 15:32
Sample: -
Note : -
Inlet : Direct
Ion Mode : EI+
Spectrum Type : Normal Ion [EF-Linear]
RT : 0.75 min
Scan# : 16
BP : m/z 328.0613
Int. : 29.88
Output m/z range : 298.0000 to 358.0000
Cut Level : 0.00 %
313350
328.06
310.05
330.06
m/z
[Elemental Composition]
Data : KK9313
Date : 17-Mar-2021 15:32
Sample: -
Note : -
Inlet : Direct
Ion Mode : EI+
RT : 0.75 min
Scan#: 16
Elements : C 17/0, H 13/0, Cl 1/0, N 2/0, O 3/0
Mass Tolerance : 1000ppm, 3mmu if m/z > 3
Unsaturation (U.S.) : -0.5 - 100.0
Observed m/z Int% Err[ppm / mmu] U.S. Composition
310.0506 39.6 -0.9 / -0.3 13.0 C 17 H 11 Cl N 2 O 2
328.0613 100.0 -0.4 / -0.1 12.0 C 17 H 13 Cl N 2 O 3
330.0603 36.5

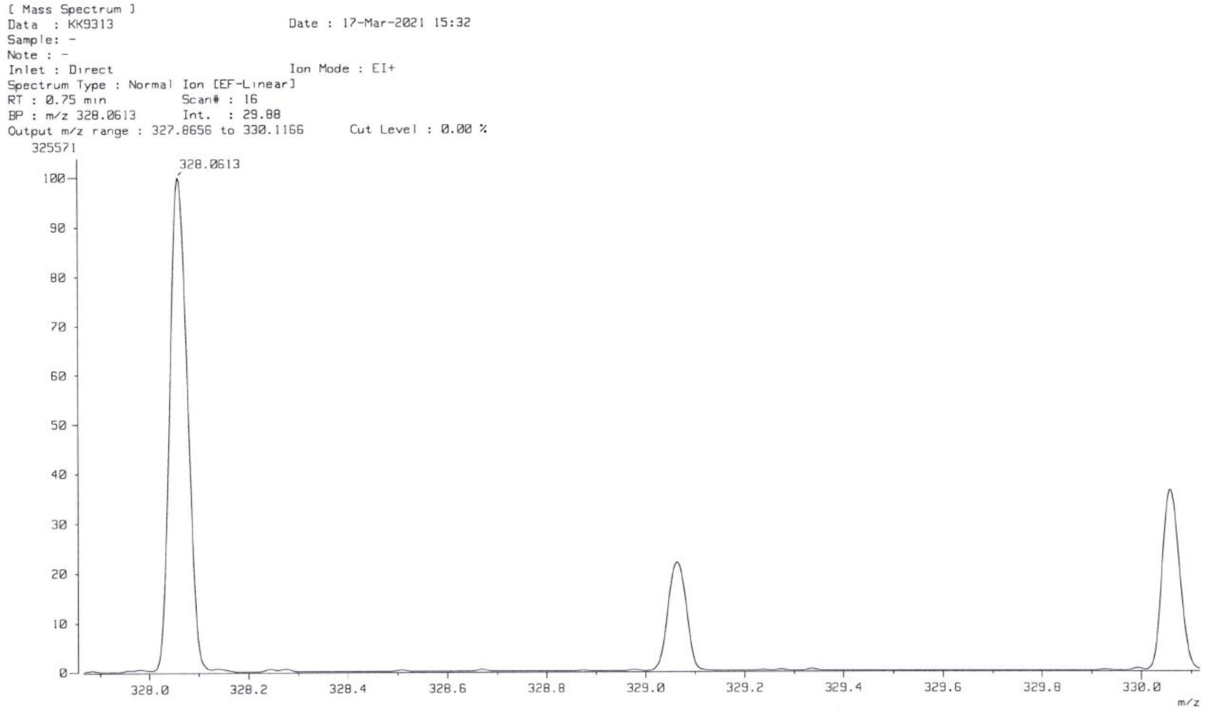
[Mass Spectrum]
Data : KK9313
Date : 17-Mar-2021 15:32
Sample: -
Note : -
Inlet : Direct
Ion Mode : EI+
Spectrum Type : Normal Ion [EF-Linear]
RT : 0.75 min
Scan# : 16
BP : m/z 328.0613
Int. : 29.88
Output m/z range : 327.8656 to 330.1166
Cut Level : 0.00 %
325571
328.0613
m/z

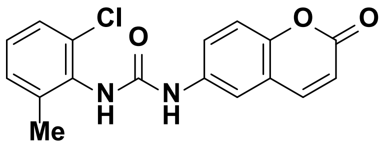

5o

[Mass Spectrum]
Data : KK9314 Date : 17-Mar-2021 15:37
Sample: -
Note : -
Inlet : Direct Ion Mode : EI+
Spectrum Type : Normal Ion [EF-Linear]
RT : 0.55 min Scan# : 12
BP : m/z 328.0616 Int. : 74.07
Output m/z range : 298.0000 to 358.0000 Cut Level : 0.00 %

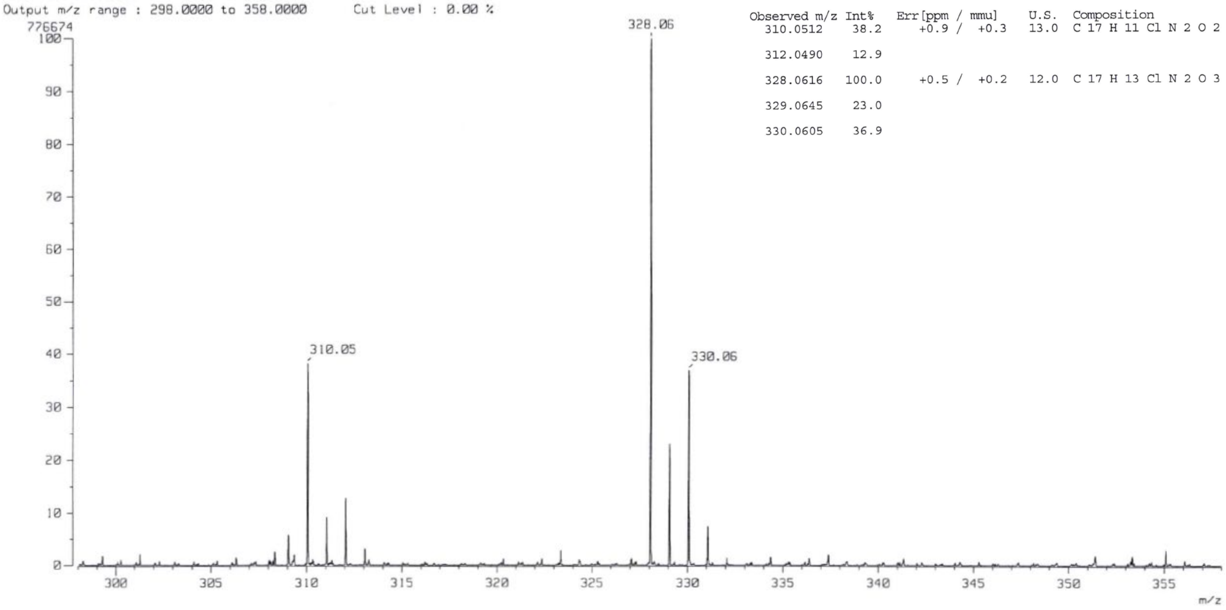

[Elemental Composition]
Data : KK9314 Date : 17-Mar-2021 15:37
Sample: -
Note : -
Inlet : Direct Ion Mode : EI+
RT : 0.55 min Scan#: 12
Elements : C 17/0, H 13/0, Cl 1/0, N 2/0, O 3/0
Mass Tolerance : 1000ppm, 3mmu if m/z > 3
Unsaturation (U.S.) : -0.5 - 100.0

Observed m/z	Int%	Err[ppm / mmu]	U.S.	Composition
310.0512	38.2	+0.9 / +0.3	13.0	C 17 H 11 Cl N 2 O 2
312.0490	12.9			
328.0616	100.0	+0.5 / +0.2	12.0	C 17 H 13 Cl N 2 O 3
329.0645	23.0			
330.0605	36.9			

[Mass Spectrum]
Data : KK9314 Date : 17-Mar-2021 15:37
Sample: -
Note : -
Inlet : Direct Ion Mode : EI+
Spectrum Type : Normal Ion [EF-Linear]
RT : 0.55 min Scan# : 12
BP : m/z 328.0616 Int. : 74.07
Output m/z range : 327.9023 to 330.1880 Cut Level : 0.00 %

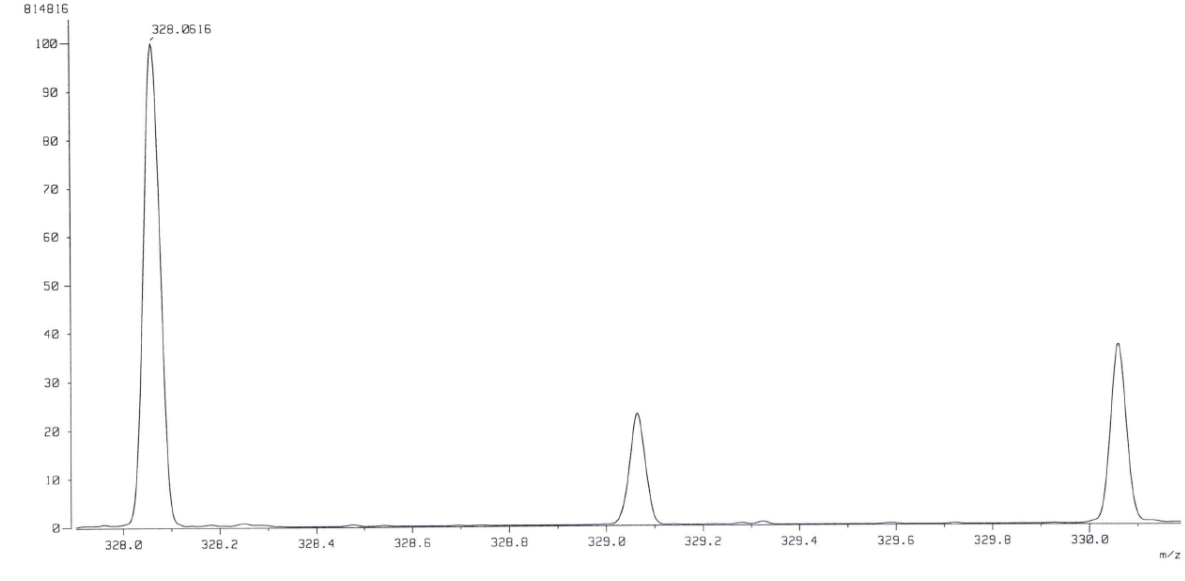

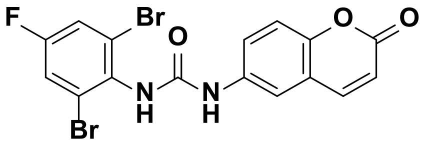
F
Br
O
O
O
N
H
N
H
Br

5p

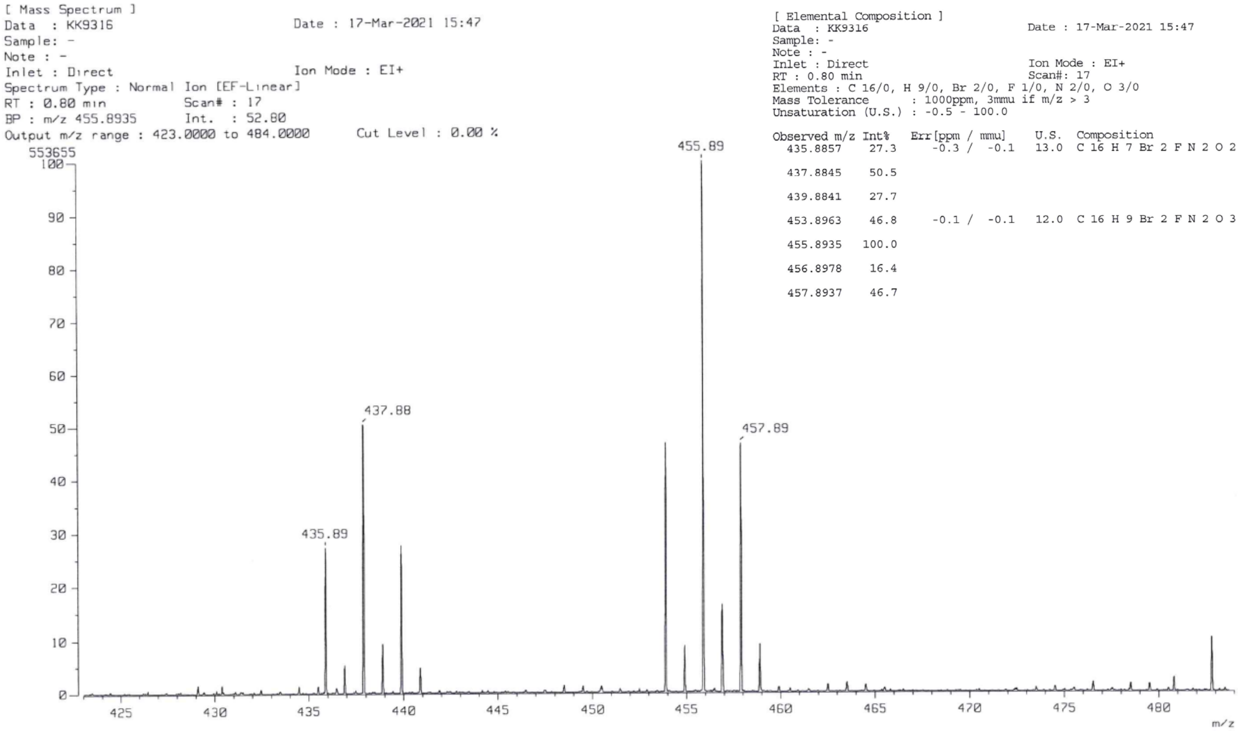
[Mass Spectrum]
Data : KK9316
Date : 17-Mar-2021 15:47
Sample: -
Note : -
Inlet : Direct
Ion Mode : EI+
Spectrum Type : Normal Ion [EF-Linear]
RT : 0.80 min
Scan# : 17
BP : m/z 455.8935
Int. : 52.80
Output m/z range : 423.0000 to 484.0000
Cut Level : 0.00 %
553655
[Elemental Composition]
Data : KK9316
Date : 17-Mar-2021 15:47
Sample: -
Note : -
Inlet : Direct
Ion Mode : EI+
RT : 0.80 min
Scan#: 17
Elements : C 16/0, H 9/0, Br 2/0, F 1/0, N 2/0, O 3/0
Mass Tolerance : 1000ppm, 3mmu if m/z > 3
Unsaturation (U.S.) : -0.5 - 100.0
Observed m/z Int% Err[ppm / mmu] U.S. Composition
435.8857 27.3 -0.3 / -0.1 13.0 C 16 H 7 Br 2 F N 2 O 2
437.8845 50.5
439.8841 27.7
453.8963 46.8 -0.1 / -0.1 12.0 C 16 H 9 Br 2 F N 2 O 3
455.8935 100.0
456.8978 16.4
457.8937 46.7
455.89
437.88
457.89
435.89
m/z

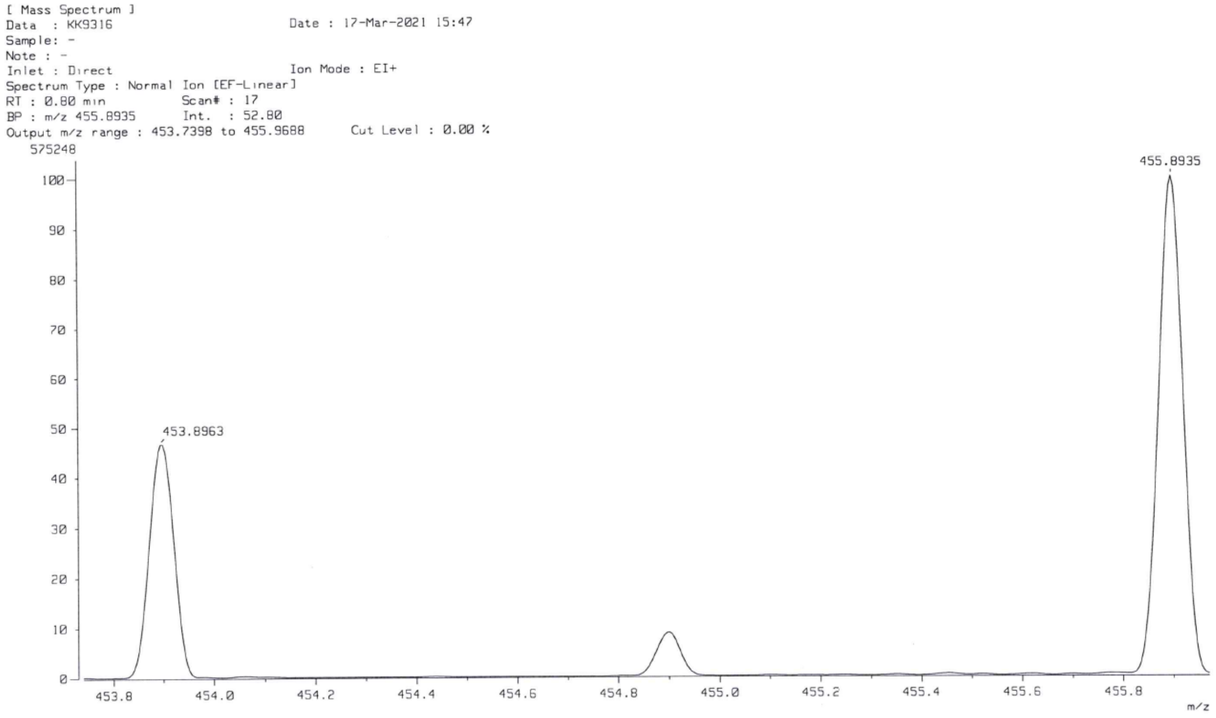
[Mass Spectrum]
Data : KK9316
Date : 17-Mar-2021 15:47
Sample: -
Note : -
Inlet : Direct
Ion Mode : EI+
Spectrum Type : Normal Ion [EF-Linear]
RT : 0.80 min
Scan# : 17
BP : m/z 455.8935
Int. : 52.80
Output m/z range : 453.7398 to 455.9688
Cut Level : 0.00 %
575248
455.8935
453.8963
m/z

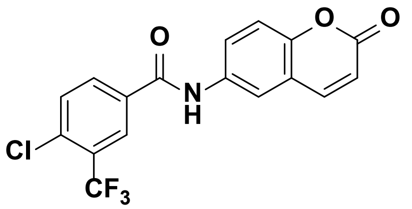

7a

[Mass Spectrum]
Data : KK9317 Date : 17-Mar-2021 15:53
Sample: -
Note : -
Inlet : Direct Ion Mode : EI+
Spectrum Type : Normal Ion [EF-Linear]
RT : 0.50 min Scan# : 11
BP : m/z 367.0225 Int. : 53.88
Output m/z range : 336.0000 to 397.0000 Cut Level : 0.00 %

[Elemental Composition]
Data : KK9317 Date : 17-Mar-2021 15:53
Sample: -
Note : -
Inlet : Direct Ion Mode : EI+
RT : 0.50 min Scan#: 11
Elements : C 17/0, H 9/0, Cl 1/0, F 3/0, N 1/0, O 3/0
Mass Tolerance : 1000ppm, 5mmu if m/z > 5
Unsaturation (U.S.) : -0.5 - 100.0

Observed m/z	Int%	Err[ppm / mmu]	U.S.	Composition
367.0225	100.0	+0.6 / +0.2	12.0	C 17 H 9 Cl F 3 N O 3
368.0253	18.1			
369.0202	35.7			

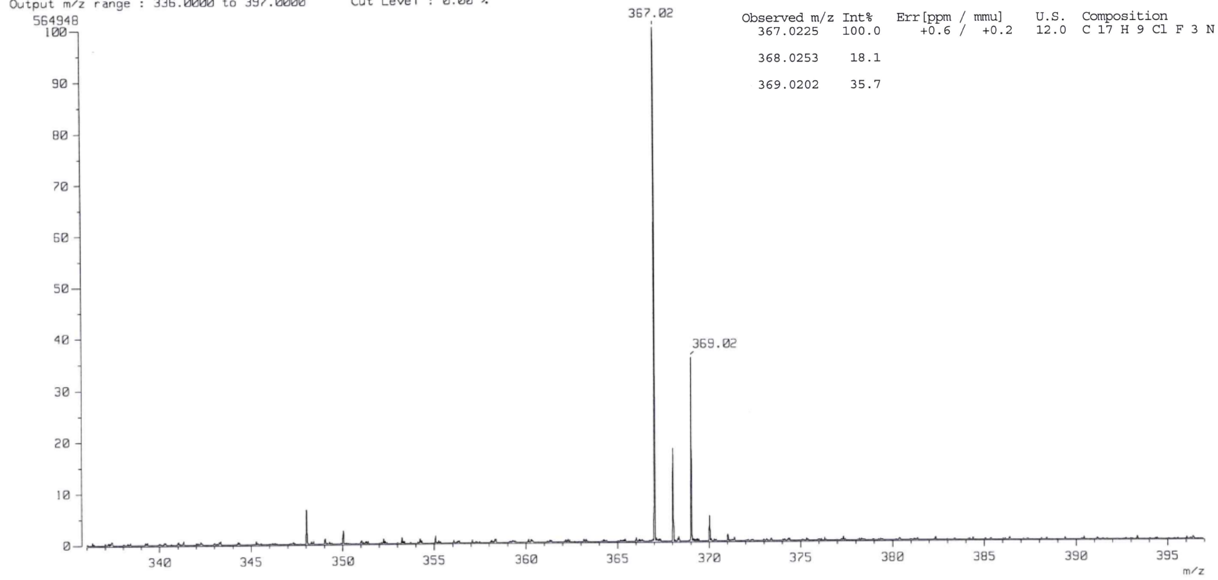

[Mass Spectrum]
Data : KK9317 Date : 17-Mar-2021 15:53
Sample: -
Note : -
Inlet : Direct Ion Mode : EI+
Spectrum Type : Normal Ion [EF-Linear]
RT : 0.50 min Scan# : 11
BP : m/z 367.0225 Int. : 53.88
Output m/z range : 366.7409 to 369.1606 Cut Level : 0.00 %

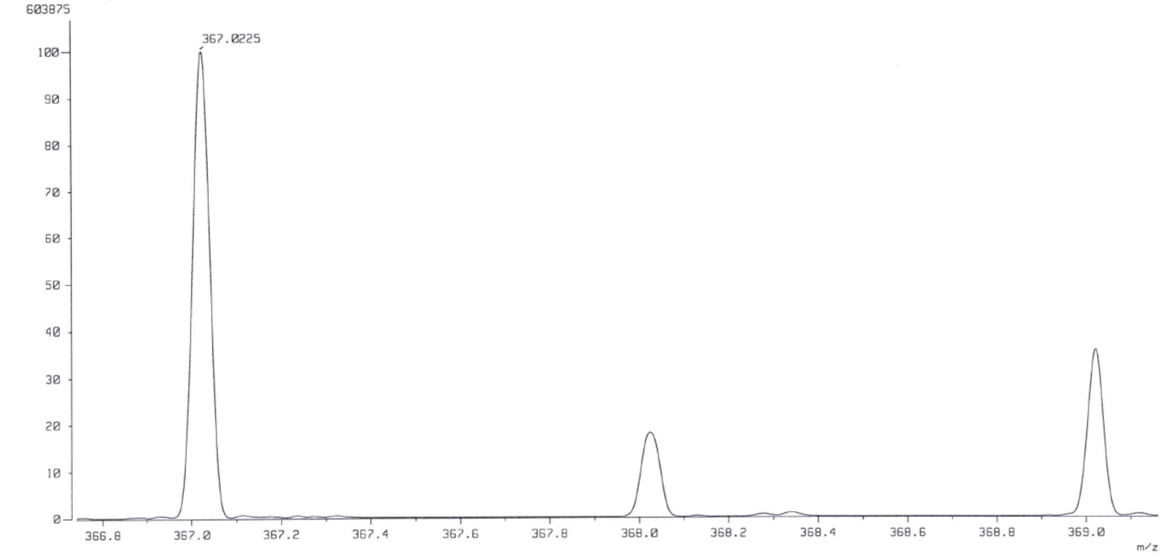

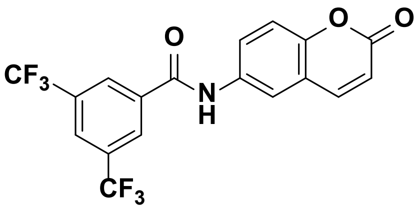

7b

[Mass Spectrum]
Data : KK9318 Date : 17-Mar-2021 15:59
Sample: -
Note : -
Inlet : Direct Ion Mode : EI+
Spectrum Type : Normal Ion [EF-Linear]
RT : 0.50 min Scan# : 11
BP : m/z 401.0490 Int. : 60.92
Output m/z range : 370.0000 to 431.0000 Cut Level : 0.00 %

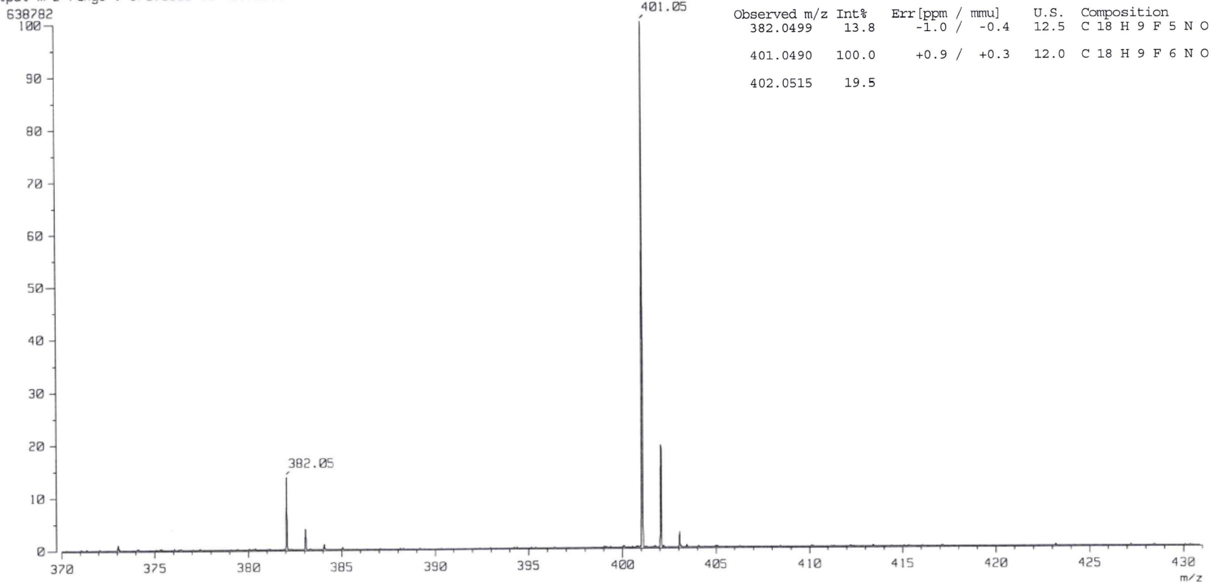

[Elemental Composition]
Data : KK9318 Date : 17-Mar-2021 15:59
Sample: -
Note : -
Inlet : Direct Ion Mode : EI+
RT : 0.50 min Scan#: 11
Elements : C 18/0, H 9/0, F 6/0, N 1/0, O 3/0
Mass Tolerance : 1000ppm, 3mmu if m/z > 3
Unsaturation (U.S.) : -0.5 - 100.0

Observed m/z	Int%	Err[ppm / mmu]	U.S.	Composition
382.0499	13.8	-1.0 / -0.4	12.5	C 18 H 9 F 5 N O 3
401.0490	100.0	+0.9 / +0.3	12.0	C 18 H 9 F 6 N O 3
402.0515	19.5			

[Mass Spectrum]
Data : KK9318 Date : 17-Mar-2021 15:59
Sample: -
Note : -
Inlet : Direct Ion Mode : EI+
Spectrum Type : Normal Ion [EF-Linear]
RT : 0.50 min Scan# : 11
BP : m/z 401.0490 Int. : 60.92
Output m/z range : 400.4972 to 402.3415 Cut Level : 0.00 %

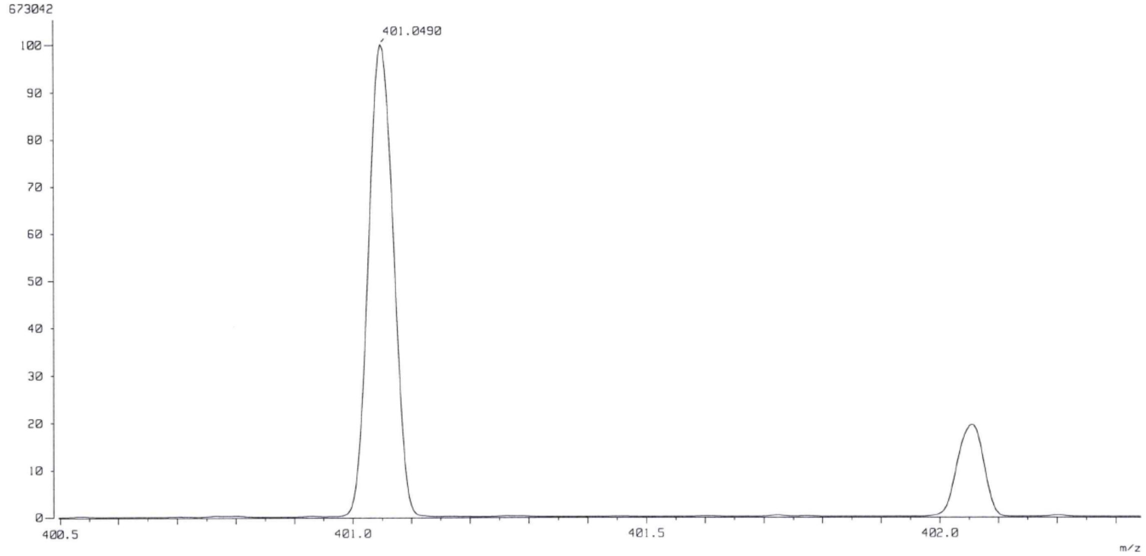

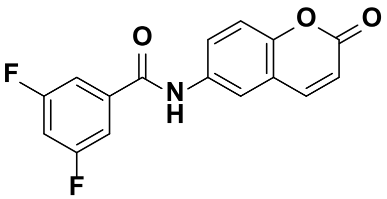

7c

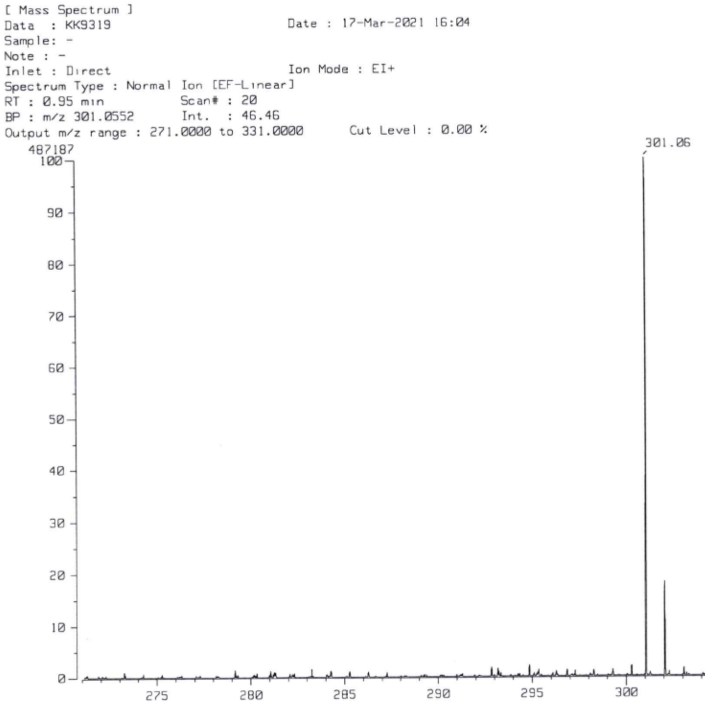

[Elemental Composition]
Data : KK9319 Date : 17-Mar-2021 16:04
Sample: -
Note : -
Inlet : Direct Ion Mode : EI+
RT : 0.95 min Scan#: 20
Elements : C 16/0, H 9/0, F 2/0, N 1/0, O 3/0
Mass Tolerance : 1000ppm, 3mmu if m/z > 3
Unsaturation (U.S.) : -0.5 - 100.0

Observed m/z	Int%	Err[ppm / mmu]	U.S.	Composition
301.0552	100.0	+0.5 / +0.2	12.0	C 16 H 9 F 2 N O 3
302.0580	18.3			

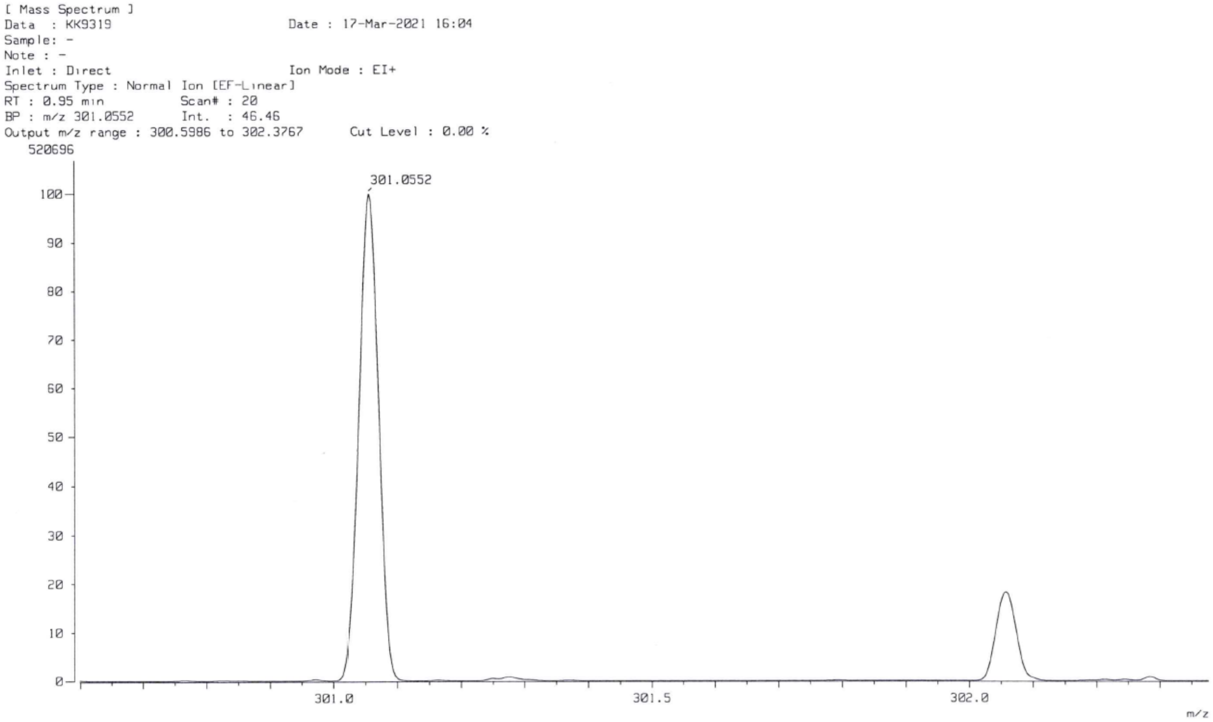

Supplement: Supplemental Material [file IENZ_A_2154603_SM4477.pdf]
